# Protection and Delivery of Phytochemicals from Passive Encapsulation to Guaranteed Self‐Assembly Induced by Amyloid Template for Chronic Disease Prevention via Modulating Microbial‐Host Crosstalk

**DOI:** 10.1002/advs.202516566

**Published:** 2026-03-13

**Authors:** Shiqi Bai, Siying Cheng, Fengguang Ma, Xiaorong Zhang, Jianan Huang, Zhonghua Liu, Bing Hu

**Affiliations:** ^1^ College of Food Science and Technology Nanjing Agricultural University Nanjing China; ^2^ National Research Center of Engineering Technology for Utilization of Botanical Functional Ingredients Changsha China; ^3^ Key Laboratory of Ministry of Education for Tea Science Hunan Agricultural University Changsha China; ^4^ College of Life Sciences Hunan Normal University Changsha China

**Keywords:** Amyloid fibrils, Encapsulation, Gut microbiota, Phytochemicals, Self‐Assembly

## Abstract

The rising incidence of chronic diseases globally has drawn widespread attention to phytochemicals, which exert targeted preventive and alleviating effect by modulating gut microbiota; thus, a potential strategy for precision nutritional interventions is offered. However, many phytochemicals may exhibit relative instability during processing and gastrointestinal tract environments, resulting in low stability, bioaccessibility, and bioavailability. Although conventional encapsulation strategies showed certain potentials in encapsulation, protection, and delivery, they still face issues such as restricted loading capacity and fragile stability, hindering the achievement of high‐dose delivery. In this context, inspired by the principle of self‐assembly in nature, a transformative was approached: to induce the realization of self‐assembly of small molecule phytochemicals from occasional to guaranteed via food protein amyloid‐like fibrils as macromolecular templates. The key point of difference from conventional encapsulation methods is that the phytochemicals self‐assembled through hierarchical structures, thereby increasing their loading and guaranteeing bioactive effects. These systems exhibit unique mechanical and stability advantages, such as shear‐thinning behavior, reversibility, and high thermal stability, which distinguish them from conventional systems. This innovation not only enhances the delivery of phytochemicals but also holds significant potential for the development of advanced biomaterials and food systems in precision nutritional interventions.

## Graphical abstract

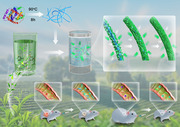

## 1 Introduction

Precision nutrition, which tailors dietary interventions to individual genetic and microbial profiles, is urgently needed to combat the global rise of chronic diseases such as obesity, type 2 diabetes (T2D), inflammation, and neurodegenerative diseases (NDs) [1, 2, 3, 4]. Emerging evidence reveals that these conditions are closely linked to dysbiosis of the gut microbiota—specific microbial communities interact with host intestinal genes to exacerbate disease progression [5, 6]. This mechanistic understanding provides a scientific basis for precision targeting. Notably, small‐molecule phytochemicals derived from edible and medicinal plants exhibit unique advantages in modulating microbial‐host crosstalk. The approaches of microbiomics, nutrigenomics, and metabolomics have been utilized with the intent to clarify the degradation, absorption, and metabolism of these phytochemical bioactives in vivo as well as their interplays with the genes and proteins playing crucial roles in the progressions of these diseases [7, 8]. Consequently, precise and personalized nutrition appeared as an emerging area in the context of diet–gene interactions, initially with human gene and recently more potentially to be realized with the exogenous genes of gut microbiota inhabiting in colon [9, 10]. Certain diseases have been demonstrated to have typical gut microbial ecosystem and structure, in particular, with specific microorganism species contributing to the development of the diseases via interacting with the genes in host intestinal tissue [5, 6, 11], which provides new targets for the bioactive phytochemicals to exert precise intervening.

The phytochemicals can be generally classified into water‐soluble and water‐insoluble ones depending on the hydrophobic and hydrophilic properties of the molecules. The hydrophobic compounds are very difficult to be dissolved in aqueous food systems and the hydrophilic phytochemicals are inclined to associate disorderly with other compounds such as proteins, starch, polysaccharides to form amorphous aggregates which cause the sharply increased turbidity and even precipitate at high concentrations. High contents of the phytochemicals and appreciable appearance of the products are difficult to be realized at the same time. This always requires the additive of large amounts of stabilizers such as surfactants, however, with high risk to have deleterious impacts on health [12]. Encapsulation of the phytochemicals with nanoparticles, microparticles, emulsions, gels, and so on has been widely investigated with the intent of protecting the phytochemicals and enhancing their bioaccessibility and bioavailability [13, 14, 15]. Still, the issue of insufficient loading content of the phytochemicals results in only a few in vivo studies with successful outcomes via the oral administration [13]. Repeated injections have to be conducted to attain suitable efficacy in the in vivo animal studies [16]. The phytochemicals were generally taken as the target substance for encapsulation with the wall materials, which is similar to embedding of drug molecules in pharmaceutics. Therefore, the low loading content in food products limits the bioaccessibility, bioavailability, and finally the bioactive functions of phytochemicals in vivo. From a precision nutrition perspective, effective dietary intervention relies on the targeted modulation of key gut microbial features associated with specific chronic disease phenotypes, which are increasingly characterized through microbiome‐ and omics‐based profiling. Enhancing the loading capacity of phytochemicals enables sufficient and controllable dosing, thereby providing a practical technical foundation for personalized dietary customization. This allows nutritional formulations to be adjusted more precisely according to patients’ specific microbiome profiles and metabolic requirements, facilitating more effective regulation of these disease‐related microbial pathways.

To address this, nature‐inspired self‐assembly strategies offer a paradigm shift: phytochemicals themselves can act as structural building blocks to actively construct ordered delivery systems by the interaction forces [17]. However, the self‐assembly of natural phytochemicals is largely determined and affected by chance [18, 19], which relies on the molecular configuration, intermolecular force, and spatial molecular arrangement. Furthermore, it is difficult for them to self‐assemble spontaneously forming the well‐defined macroscopic soft matter products, with only a few successful examples until now. In fact, proteins (peptides), nucleic acids, polysaccharides or their mimics, have been demonstrated to self‐assemble to ordered fibrils [20, 21]. Therefore, hierarchical structures are expected to be constructed through the self‐assembly of food macromolecules and small molecular phytochemicals, during which the biomacromolecule fibrils could act as the scaffolding to support the adhesion and further self‐assembly of the small molecules. The key point distinct from the approach of traditional “encapsulation” is that small molecular phytochemicals act as not only the bioactive ingredients but also the building blocks for the food hierarchical structures made thereof. These systems exhibit shear‐thinning, reversibility, and high thermal stability, making them more resilient and effective than conventional systems [22, 23, 24]. This approach has significant implications for advancing precision nutrition, offering both a stable delivery system and an effective means to integrate functional ingredients into food formats.

In this review, we focus on the mechanisms of action and application progress of phytochemicals in chronic diseases, common encapsulation methods for phytochemicals, and the formation and application of edible soft materials through self‐assembly. However, existing phytochemical self‐assembled materials suffer from the issues such as sporadic occurrence and poor universality. Therefore, we have emphasized the template role of amyloid fibrils in the self‐assembly process and their application progress, aiming to provide synthetic insights for future plant chemical self‐assembly, thereby better meeting the demand for edible food‐based active substances in precision nutritional interventions.

## 2 Phytochemicals for Health Promotion Associating With Regulation of Gut Microbiota

**FIGURE 1:**
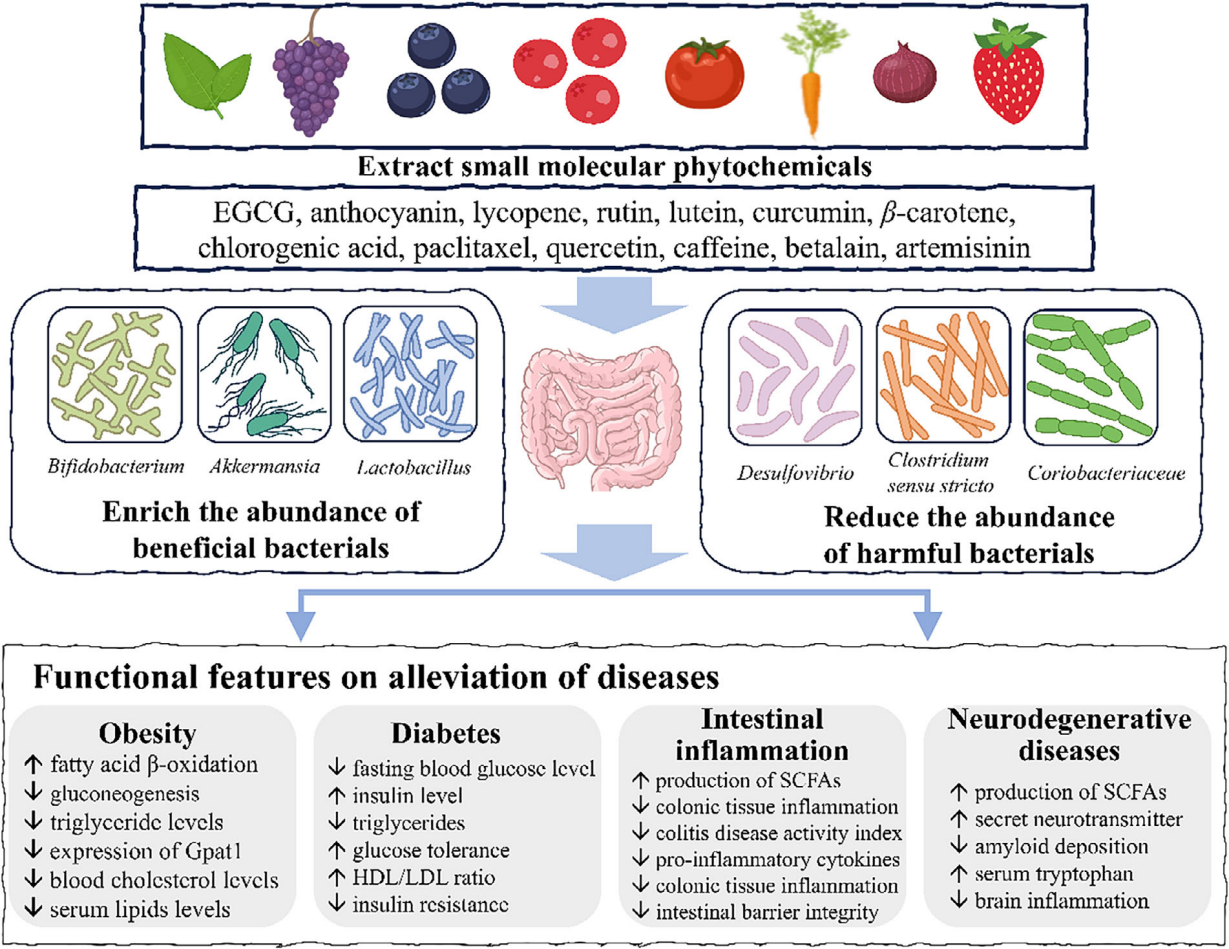
Phytochemicals alleviate diet‐related chronic diseases by regulating gut microbiota.

Phytochemicals with bioactivities of health promotion can be recognized as the gifts from nature to human beings. The plant kingdom produces hundreds of thousands of low‐molecular‐weight organic compounds. Among them, the phytochemicals, such as polyphenols, carotenoids, saponins, phytosterols, and glucosinolates, belong to the plant secondary metabolites which mediate plant–environment interactions. Phytochemicals are also usually well‐known for their health‐promotion functions, such as antioxidant, anti‐inflammation, anticancer, anti‐obesity as well as immunoregulatory activities, which have been attributed in recent studies to the regulation of gut microbiota. In this section, we summarized the effects of different types of phytochemicals on promoting health such as prevention of obesity, diabetes, intestinal inflammation, and NDs, not only in animal studies but also in the human volunteer investigations. It could be found that the effects of the polyphenols on prevention of these chronic diseases were always accompanied with the regulation of gut microbiota structure, changing the relative abundance of certain bacterial (Figure 1) and the metabolism products of gut microbiota such as short chain fatty acids, bile acids, and so on. Although its mechanism of action through the gut microbiota provides new targets for future translational research, its application in clinical treatment still requires extensive rigorous human clinical trials for validation.

In the context of food science and nutrition, precision nutrition is widely defined as an individualized, omics‐informed approach that tailors dietary interventions based on inter‐individual differences in genetic background, gut microbiota composition, and metabolic phenotype [25]. This review focuses on mechanism‐guided nutritional strategies that may support its implementation by targeting biological pathways commonly involved in diet‐related chronic diseases. In particular, we summarize evidence showing that phytochemicals can exert predictable biological effects on host–microbiome interactions and phenotype‐associated gene expression. Recent studies indicate that phytochemicals improve metabolic health by modulating gut microbial metabolism and downstream signaling pathways, thereby contributing to the prevention and mitigation of diet‐related chronic diseases [26].

### 2.1 Anti‐obesity and Attenuating Diabetes by Phytochemicals Accompanied With Regulating Gut Microbiota

Growing evidence highlights the pivotal role of gut microbiota dysbiosis in metabolic disorders [27], including obesity and T2D. Phytochemicals, particularly polyphenols from dietary plants, emerge as potent modulators of microbial ecology. These compounds reshape gut microbial composition to enhance beneficial metabolites and suppress pathogenic pathways, thereby ameliorating metabolic dysregulation. Tea polyphenols in Pu‐erh and Fuzhuan brick tea, notably catechins, alleviate obesity by modulating gut microbiota—enriching beneficial taxa like *Akkermansia muciniphila* and *Faecalibacterium prausnitzii* while suppressing harmful *Ruminococcaceae* and *Peptococcaceae*, thereby improving metabolic markers in high‐fat diet models [28, 29, 30]. Similarly, proanthocyanidins from grapes, cranberries, blueberries, cranberries, and apples reduce adiposity and enhance insulin sensitivity in obese mice, correlating with increased *Akkermansia* and *Bacteroides* alongside decreased *Lactobacillus* [31, 32, 33, 34, 35]. Anthocyanins were further demonstrated to have anti‐obesity effects via gut microbiota remodeling, notably elevating *Akkermansia muciniphila* abundance [33, 34, 35, 36, 37, 38, 39]. In T2D, tea polyphenols (green tea EGCG, Liupao tea extracts) lower blood glucose and LDL cholesterol while restoring microbial balance—increasing *Lactobacillus*, *Bifidobacteriaceae*, and short‐chain fatty acid (SCFA) producers (such as *Prevotella*, *Bacteroides*) and reducing *Enterobacteriaceae* [40, 41, 42, 43, 44]. These findings collectively highlight the capacity of phytochemicals to target gut microbiota dysbiosis, linking specific microbial shifts, such as decreasing Firmicutes/Bacteroidetes (F/B) ratio to metabolic improvements in obesity and diabetes.

### 2.2 Alleviating Intestinal Inflammation by Phytochemicals Accompanied With Regulating Gut Microbiota

Most current therapies for inflammatory bowel disease (IBD) are aimed to suppress the immune response in the host including applications of steroids and biologicals such as anti‐TNF or anti‐integrin therapies, which do not directly target the microorganisms that are involved in the pathogenesis of IBD [45]. Current therapy for IBD can be considered as generally supportive rather than curative and has long‐term side effects [46, 47]. More attentions have been paid to the phytochemicals originating from edible plants. The polyphenolic compounds extracted from apple [48], pomegranate [49, 50], propolis [51], holly [52], and mango [53] were found to alleviate intestinal inflammation induced by DSS or lipopolysaccharide, including the inhibition of the proinflammatory factors in blood and the expression of pro‐inflammatory genes in intestinal tissue, the promotion of intestinal barrier integrity, the alleviation of the disease index, and so on. At the same time, the modulation of gut microbiota could be observed, including an increase in the abundance of beneficial bacteria such as *Prevotella*, *Bacteroides*, *Akkermansia*, and *Lactobacillus* (*Lactobacillus plantarum*, *Lactobacillus reuteri*, and *Lactobacillus lactis*), as well as the phylum Verrucomicrobia. Conversely, a decrease was observed in the abundance of the phylum Firmicutes and certain Bacteroides populations. The enhancement of SCFA production was also found [54]. The anti‐inflammation activity of these polyphenolic compounds was partly ascribed to their effect on preventing the increase of reactive oxygen species (ROS) levels [50, 55].

Consumption of phytochemicals were further demonstrated to promote health with combination of regulating gut microbiota in human studies. The in vivo metabolism of *trans*‐resveratrol, the major polyphenol in wine, by human gut microbiota was investigated to produce dihydroresveratrol, 3,4′‐dihydroxy‐*trans*‐stilbene and 3,4′‐dihydroxybibenzyl [56]. Prebiotic potentials were found for juçara berry and wild blueberry, promoting the relative abundance of *Bifidobacterium spp*. and *Akkermansia muciniphila* and increasing the level of excreted acetate in volunteers’ gut microbiota [57, 58]. The overweight‐obese subjects consuming pomegranate extract revealed the increase of microorganisms important for maintaining normal balance of gut microbiota and gut barrier function, particularly *Bacteroides*, *Faecalibacterium*, *Butyricicoccus*, *Odoribacter*, and *Butyricimonas*, as well as decreased pro‐inflammatory microorganisms including *Parvimonas*, *Methanobrevibacter*, and *Methanosphaera* [59]. Remarkably, reduction of endotoxemia marker lipopolysaccharide‐binding protein was found to be significantly associated with both *Faecalibacterium* and *Odoribacter* increase and *Parvimonas* decrease [59]. Intake of mango alleviated IBD symptoms in the participants and beneficially altered the fecal microbial composition. Specifically, it significantly increased the abundances of *Lactobacillus* spp, including *Lactobacillus plantarum*, *Lactobacillus reuteri*, and *Lactobacillus lactis*, which was accompanied by an increase in fecal butyric acid production [53].

### 2.3 Alleviation on Symptom Burden of NDs With Regulating Gut Microbiota

There is growing evidence that the gut microbiota makes essential contributions to brain development, and as a result, the gut microbiota can be used as the hub for the alleviation on symptom of NDs. Moreover, phytochemicals have shown to interact with the gut microbiota, thereby influencing brain function. Polyphenols play a vital role in protecting NDs via affecting the composition and relative abundance of the gut microbiota. Intake of anthocyanin‐rich supplements can increase the abundance of the main genera like *Bifidobacterium, Blautia*, and *Faecalibacterium*, as well as minor genera like *Prevotella* and *Akkermansia* [60]. The increase of such gut microbiota can promote the production of SCFAs, particularly butyric acid, and they serve as a protector of the central nervous system by metabolically regulating immune cells [61]. In addition, SCFAs also act as signaling molecules to play an essential role in gut‐brain communication [62], among which butyrate is one of the key factors in maintaining the integrity of the blood‐brain barrier [63]. The increased *Lactobacillus* and *Bifidobacterium* can also secrete neurotransmitter gamma‐aminobutyric acid (GABA), which was reported to be beneficial in alleviating NDs [64]. Tea polyphenols also have the function of slowing down or protecting the occurrence of NDs. The intake of tea polyphenols can significantly affect the abundance and diversity of intestinal flora, that is, increasing the relative abundance of beneficial and probiotic bacteria, including *Lactobacillus, Bifidobacterium* spp, *Akkermansia muciniphila*, *Adlercreutzia*, *Allobaculum*, and *Firmicutes* [65]. The proliferation of *Akkermansia muciniphila* favors the secretion of serum tryptophan, which promotes the secretion of neurotransmitter 5‐hydroxytryptamine (5‐HT), thus playing a role in protecting brain function [66]. Curcumin and Resveratrol can also have a similar effect. The ingested curcumin is modified by various microorganisms, producing different metabolites that are more active than itself [67]. On the other hand, curcumin administration can alter the composition of bacterial taxa, including *Lactobacillaceae*, *Rikenellaceae*, *Bacteroidaceae*, *Bacteroides*, *Prevotellaceae*, *Parabacteroides*, and *Prevotella*, all of which are related to Alzheimer's disease [68]. Resveratrol can impact the gut‐brain axis in the way of affecting gut microbiota diversity, and contributing to the balance between gut and brain function through the 5‐HT system [69].

## 3 Encapsulation of Phytochemicals

**FIGURE 2:**
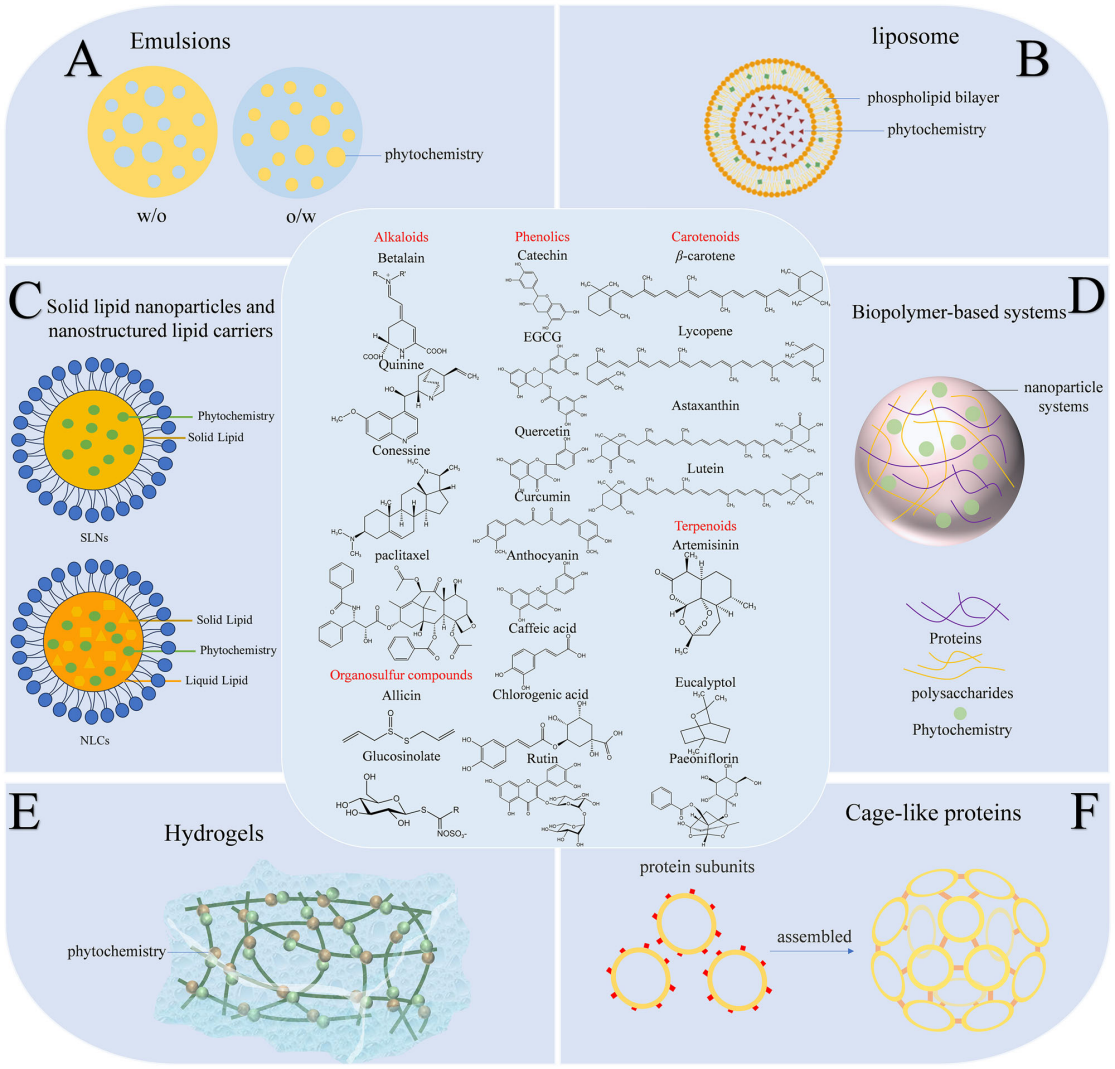
Different encapsulation technologies in the food industry. A) Structure of two types of emulsions: oil‐in‐water (O/W) and water‐in‐oil (W/O); B) structure of liposome; C) structure of solid lipid nanoparticles (SLNs) and nanostructured lipid carriers (NLCs); D) structure of biopolymer‐based systems: Proteins and polysaccharides form a verity of micro or nanoparticle systems to encapsulate phytochemicals; E) structure of hydrogels: 3D cross‐linked networks assembled by polymer chains; F) structure of cage‐like proteins: individual subunits spontaneously arrange into highly ordered patterns via noncovalent but specific interactions. All chemical structures are obtained in KingDraw.

Phytochemicals have great potential to reduce the risk of a series of diseases such as obesity, diabetes, cardiovascular disease, high blood pressure, and certain types of cancer. However, the poor bioavailability restrains their application in preventing diseases [70]. Encapsulation system is becoming more and more popular in protecting bioactive compound and enhancing the functional activity of phytochemicals. None of the encapsulation systems can be considered as a universally applicable technique to encapsulate phytochemicals due to the fact that each compound has different characteristics [15]. The most widely used encapsulation technologies in the food industry are emulsion‐, liposome‐, biopolymer‐, and hydrogel‐based systems, which are discussed in more detail in the remainder of this section (Figure 2).

### 3.1 Emulsions

**FIGURE 3:**
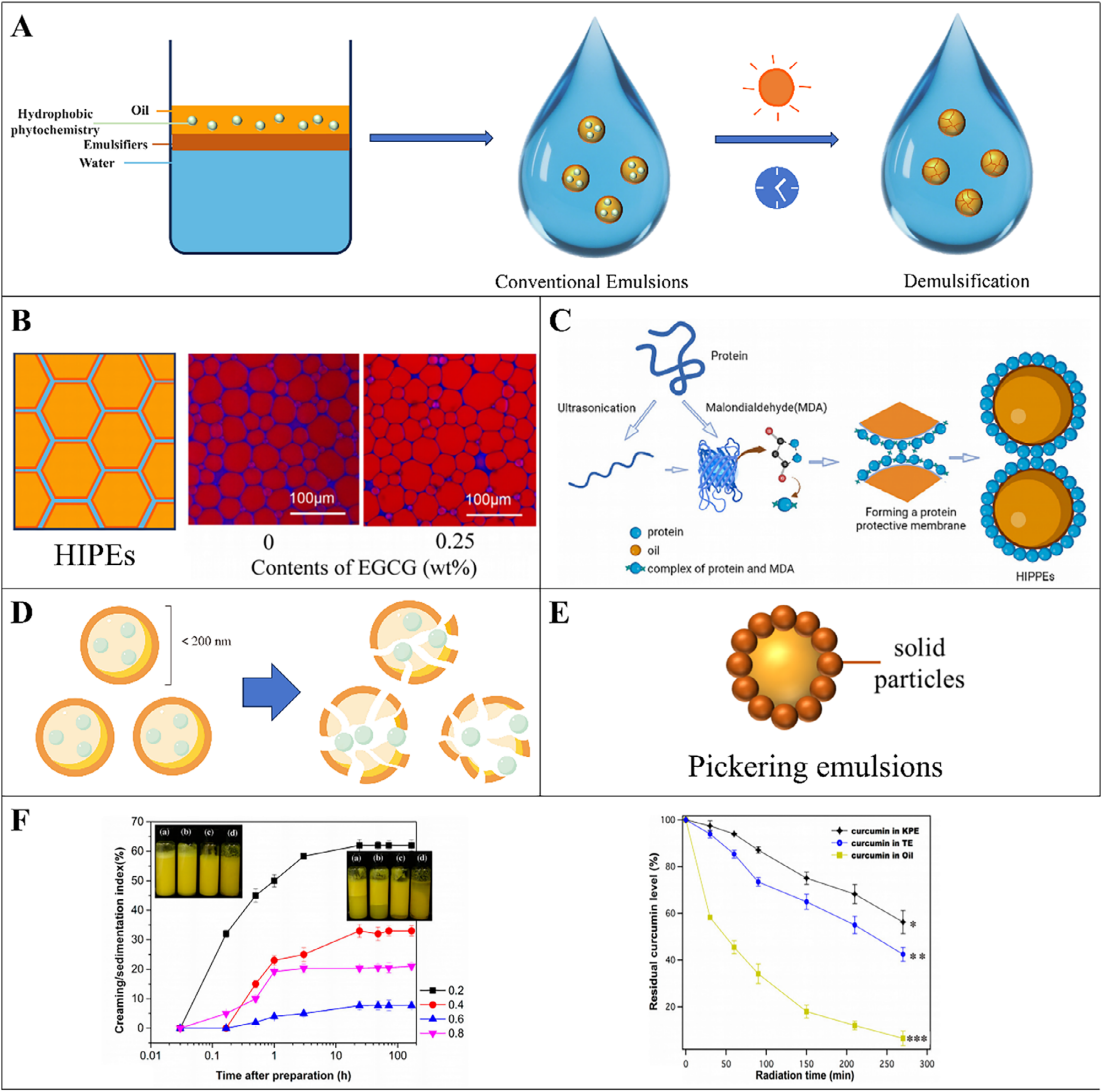
A) Schematic diagram of conventional emulsions; B) Left: Schematic diagram of high internal phase emulsions (HIPEs); right: confocal laser scanning microscopy (CLSM) images of the HIPEs (oil volume fraction of 80%) stabilized by the polyphenol‐amyloid fibril supramolecules with 0 and 0.25 wt% EGCG; Reproducted with permisson [79]. Copyright 2022, American Chemical Society; C) Schematic diagram of the formation of the high internal phase Pickering emulsion by modified sturgeon myofibrillar protein for the encapsulation of quercetin; Reproducted with permisson [89]. Copyright 2023, Elsevier; D) Schematic diagrams of the structure of nanoemulsions and the release procession; E) Schematic diagram of Pickering emulsions; F) Left: Creaming/sedimentation index of Kafirin nanoparticle‐stabilized Pickering emulsion (KPE) at various oil fractions along with storage time; Right: Residual curcumin level in KPE, Tween 80‐stabilized emulsion (TE), and oil; Reproducted with permisson [102]. Copyright 2023, American Chemical Society.

Emulsions are colloidal dispersions that contain at least two immiscible fluids phases (normally water and oil), with one of them being dispersed in the other in the form of small droplets [71]. Proteins, peptides, lipids, and low‐molecular‐weight surfactants are commonly applied as emulsifiers that help in achieving stabilization of oil and water phases by lowering the interfacial tension at the oil/water interface [72]. Emulsions can be classified into two types: oil‐in‐water (O/W) and water‐in‐oil (W/O) emulsion (Figure 2A). Generally, oil‐in‐water emulsions, whose continuous phase is aqueous, are used to encapsulate hydrophobic substances, which consist of oil droplets dispersed in a continuous aqueous phase, with emulsifiers stabilizing the oil‐water interface (Figure 3A) [73]. Hydrophobic phytochemicals are distributed in the oil droplets, which increases their water‐dispersibility and stability. For instance, encapsulation of β‐carotene in emulsion protected it from chemical degradation in the process of storage and increased the bioaccessibility in simulated GIT conditions [74]. Encapsulating resveratrol using emulsions showed strong storage stability and high bioavailability [75]. However, conventional emulsions are susceptible to breakdown over time or when exposed to certain environmental stresses during their production and storage (Figure 3A). In addition, they have a limited ability to control the release profile of encapsulated components. These shortcomings limit their certain applications in the real product processing in food and medicine industry. Advanced emulsion technologies have been developed in recent years, which show better performances on account of optimized stability and increased bioaccessibility of encapsulated ingredients.

In general, a higher lipid content leads to a higher bioaccessibility of phytochemicals, despite the limited solubility of some phytochemicals in lipids. A high content of lipid in emulsion can promote the formation of more mixed micelles, which is available to solubilize the phytochemicals in intestine after lipid digestion [76]. High internal phase emulsions (HIPEs) refer to emulsions typically having a disperse phase volume fraction that exceeds the close‐packing limit (around 74%) [77], thereby carrying more phytochemicals [78]. At such high concentrations, the droplets are so tightly packed together and may adopt nonspherical shapes (Figure 3B) [79], thus the HIPEs typically have semi‐solid textures [71]. HIPEs have attracted considerable interest in recent years for some food applications because of their semi‐solid textures and encapsulating high contents of hydrophobic bioactive substances. HIPEs stabilized by the (−)‐epigallocatechin gallate (EGCG)‐amyloid fibril supramolecules were used to encapsulate lutein, a high loading content of lutein of up to 10 mg/mL in the prepared HIPEs was achieved, and the stability of lutein against ultraviolet irradiation, heat, iron, and hydrogen peroxide was also significantly increased [79]. HIPEs stabilized by pea protein fibrils with internal phase volume fraction up to 90% were applied to encapsulate lutein, high loading contents and high stability against ultraviolet irradiation, heat and iron of lutein were realized at the same time [80]. HIPEs stabilized with genipin crosslinked chitosan (CS) microgels, were found to dramatically increase the loading contents of β‐carotene, up to 2 wt% with only 0.1 wt% microgel as the emulsifiers, furthermore, encapsulation of β‐carotene in the HIPE systems could resist ultraviolet irradiation, thermal treatment, iron ions and hydrogen peroxide in aqueous phase [81]. In addition, Encapsulation of β‐carotene within a various protein‐stabilized HIPEs showed increased heat stability and bioaccessibility [82, 83, 84]. Moreover, a series of other hydrophobic phytochemicals, including curcumin [85, 86] and polymethoxyflavones (PMFs) [87, 88], encapsulating in HIPEs have also been shown to appreciably enhance the stability and bioaccessibility. High internal phase Pickering emulsions stabilized by modified sturgeon myofibrillar were applied to encapsulate quercetin and the in vitro digestion result showed that the combined modified protein‐stabilized high internal phase Pickering emulsions significantly improved the rate of lipid digestion and the increased bioavailability of quercetin while reducing the degradation (Figure 3C) [89].

Nanoemulsions are similar to the conventional emulsions but contain very small droplets with the diameter size < 200 nm (Figure 3D) [90]. Nanoemulsions possess a range of properties such as tiny droplet size, large surface area, enhanced dispersion of active hydrophobic components, and increased absorption [91]. The small size of nanoemulsions can help promoting their extension, and the wide surface area may facilitate the disintegration of lipid droplets and releasing of the encapsulated components (Figure 3D) [91]. The loading of poorly adsorbed hydrophobic phytochemicals into the oil phase of nanoemulsion helps in protecting them from degradation and increasing their bioavailability, thus promoting their effective delivery to target sites [92]. Encapsulation of a series of hydrophobic phytochemicals, such as β‐carotene [93], lycopene [94], curcumin [95], and resveratrol [96], within nanoemulsions can increase their bioaccessibility in the systems of in vitro simulated GI tract models. Moreover, in vivo studies using nanoemulsions to encapsulate certain bioactive compounds, including vitamin D_3_ [97], vitamin E [98], and coenzyme Q_10_ [99], have also shown increased bioavailability with oral administration in animals.

Emulsions stabilized by solid particles are referred as Pickering emulsions [100]. Unlike conventional emulsions stabilized by molecular emulsifiers (like surfactants, phospholipids, proteins, or polysaccharides), Pickering emulsions are stabilized by colloidal particles and the particles may be organic (proteins, polysaccharides, polyphenols, fat crystals, etc.) or inorganic (silica, calcium carbonate, and hydroxyapatite) in nature (Figure 3E) [100]. Because of the tight attachment of the colloidal particles to the droplet surfaces, Pickering emulsions are typically much more resistant to Ostwald ripening and coalescence than conventional emulsions [71]. Food‐grade particles are often sustainably sourced and entirely nontoxic and thereby safer than molecular surfactants. Increasing amounts of studies have focused on the development of surfactant‐free Pickering emulsions to avoid potential safety concerns associated with high levels of synthetic surfactants used in food products. Fucoxanthin encapsulated with phycocyanin/lysozyme nanocomplexes stabilized Pickering emulsions showed enhanced thermal stability and bioavailability in the process of in vitro digestion [101]. Pickering emulsions stabilized by kafirin nanoparticles exhibited less lipid oxidation and higher curcumin stability under UV radiation than those stabilized by Tween 80, which was linked to the formation of kafirin particles‐constructed physical barrier around the oil droplets, as well as the interfacial antioxidant effect (Figure 3F) [102].

Emulsion science and technology have gained great advances in decades and create a diverse range of products in the food industrial [103]. Although they possess improved functional properties, such as controlled digestion behavior and increased bioavailability of bioactive compounds, advanced emulsions are relatively expensive and difficult to prepare. The conventional oil‐in‐water emulsions are still the most widely used emulsions in the food industrial [71]. The urgent need for commercially viable large‐scale preparation procedures and materials for the production of advanced emulsions is an emerging challenge. Moreover, there is still a great potential to improve the delivery of bioactive compounds, and more detailed information on the biological fate of advanced emulsions also required much more in‐depth research.

### 3.2 Liposomes

**FIGURE 4:**
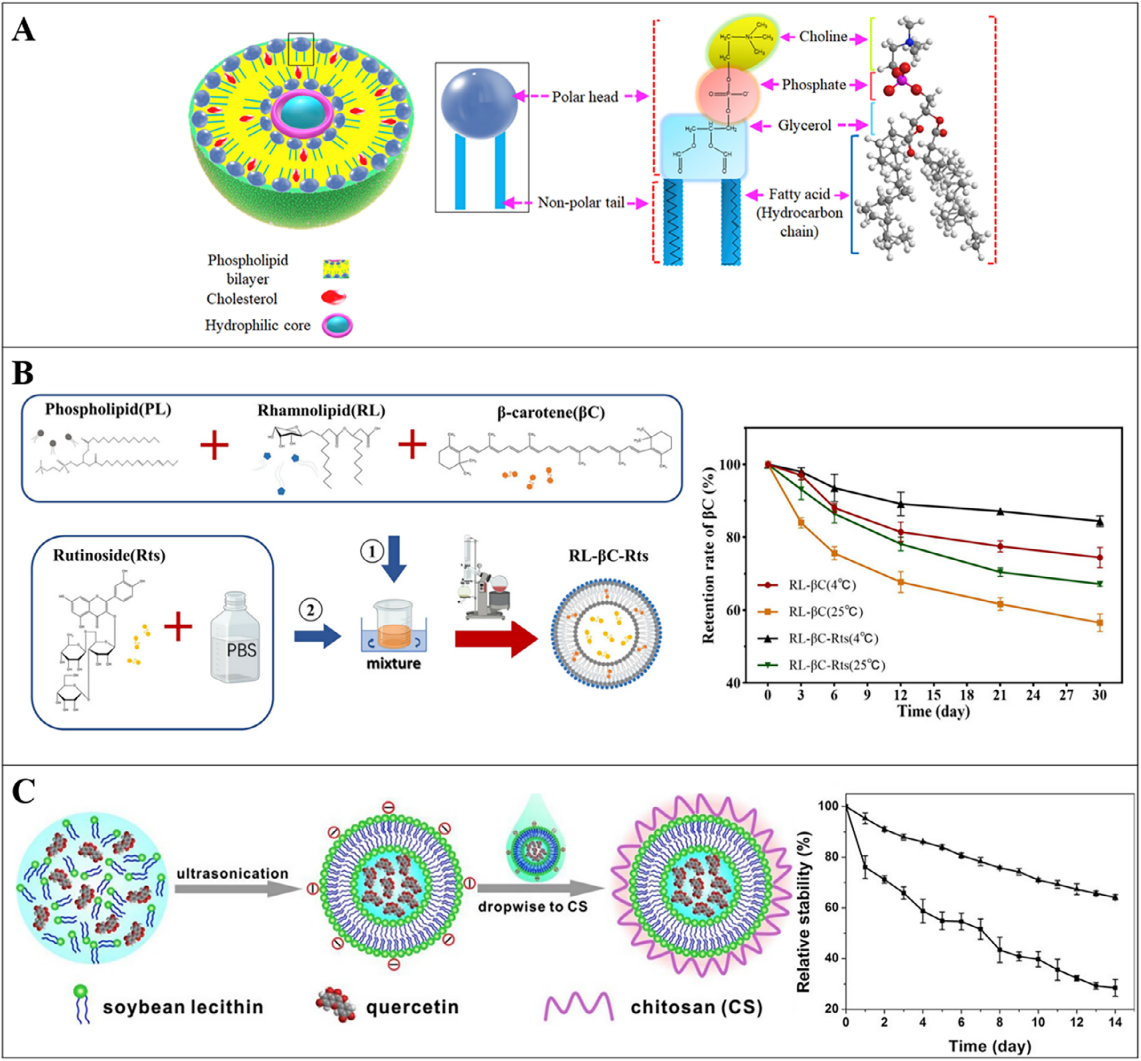
A) Cross‐section structure of liposome, made of phospholipid and cholesterol showing the magnified molecular structure of a phospholipid that consists of a polar head and a nonpolar tail. Phospholipid head is hydrophobic and comprises choline, phosphate, and glycerol, while the tail is a hydrocarbon chain that shows lipophilicity; Reproducted with permisson [106]. Copyright 2019, Elsevier; B) Left: Preparation mechanism of rhamnolipid (RL) and β‐carotene (βC) co‐encapsulated liposomes; Right: Observation of the retention of β‐carotene (βC) in RL‐βC, RL‐Rts, and RL‐βC‐Rts stored at 4 and 25 °C for 30 days; Reproducted with permisson [108]. Copyright 2023, Wiley; C) Left: Scheme of the preparation process of quercetin loaded polymeric nanocapsules (Q‐NPs) by electrostatic deposition; Right: Relative stability of native quercetin (■) and Q‐NPs (▼) stored at 37 °C under natural light; Reproducted with permisson [111]. Copyright 2017, Elsevier.

Liposomes are microscopic artificial lipid vesicles composed of phospholipid bilayers [104], making it amphiphilic in nature. Apart from phospholipids as their primary part, cholesterol, various polymers, and sterols can also be used for the formulation of liposomes [105]. Liposomes can encapsulate hydrophilic, amphiphilic, or hydrophobic components within their structures (Figure 2B). For instance, hydrophobic compounds can be embedded into the phospholipid bilayer, hydrophilic compounds can be wrapped in the aqueous center, and amphiphilic compounds can reside near the bilayer surface (Figure 4A) [106]. Various methods for the preparation of liposomes have been developed. Conventional methods such as thin‐film hydration, reverse‐phase evaporation, and ethanol injection have been widely applied. Moreover, supercritical fluid and microfluidic methods have gained considerable attention due to their advantages in terms of preparation efficiency and structural controllability of liposome [104].

Liposome can be applied to encapsulate bioactive components and protect them from environmental factors [107]. Due to their compositional and structural similarity to cell membranes, liposomes can also enhance the stability and bioavailability of encapsulated functional components. Co‐encapsulation of rutinoside and β‐carotene in rhamnolipid modified liposomes through an ethanol injection method fabricated a novel cholesterol‐free composite delivery system. This liposome system showed dependable storage stability, antioxidant activities, and antibacterial ability, and it also improved the bioavailability of β‐carotene during gastrointestinal digestion (Figure 4B) [108]. The antioxidant of capsaicin was markedly increased after the encapsulation with nanoliposomes, which further significantly alleviated the oxidative stress by reducing the hepatic lipid peroxidation and ROS production in livers of rat [109]. The encapsulation in a multilamellar liposome system effectively protected Vitamins C and E from degradation under thermal treatment and their antioxidative activities were also maintained for 7 days at 4 °C. In addition, adding liposome‐loaded vitamins C and E to chocolate milk hardly altered its organoleptic properties [110]. Encapsulation of the quercetin with CS‐coated nano‐liposomes showed a high encapsulation efficiency of 71.14%; in addition, improved storage stability and antioxidant activity were also observed (Figure 4C) [111].

Being a kind of versatile delivery systems [14], liposomes is potential to be widely used in the food, pharmaceutical, and cosmetic industries [112, 113]. Currently, various types of liposome‐based foods and dietary supplements have been clinically approved and commercially available [114]. However, due to relatively high cost and their sensitivity to light, high temperatures, salts, and pH, liposomes are not commonly used in the food and beverage industry [106]. The application and marketability of liposomes in the food industry rely on systematic and comprehensive research. More basic and applied research that focus on enhancing liposome structure, stability, and safety as well as developing more cost‐effective and rapid methods of liposome preparation are all required [104].

### 3.3 Solid Lipid Nanoparticles and Nanostructured Lipid Carriers

**FIGURE 5:**
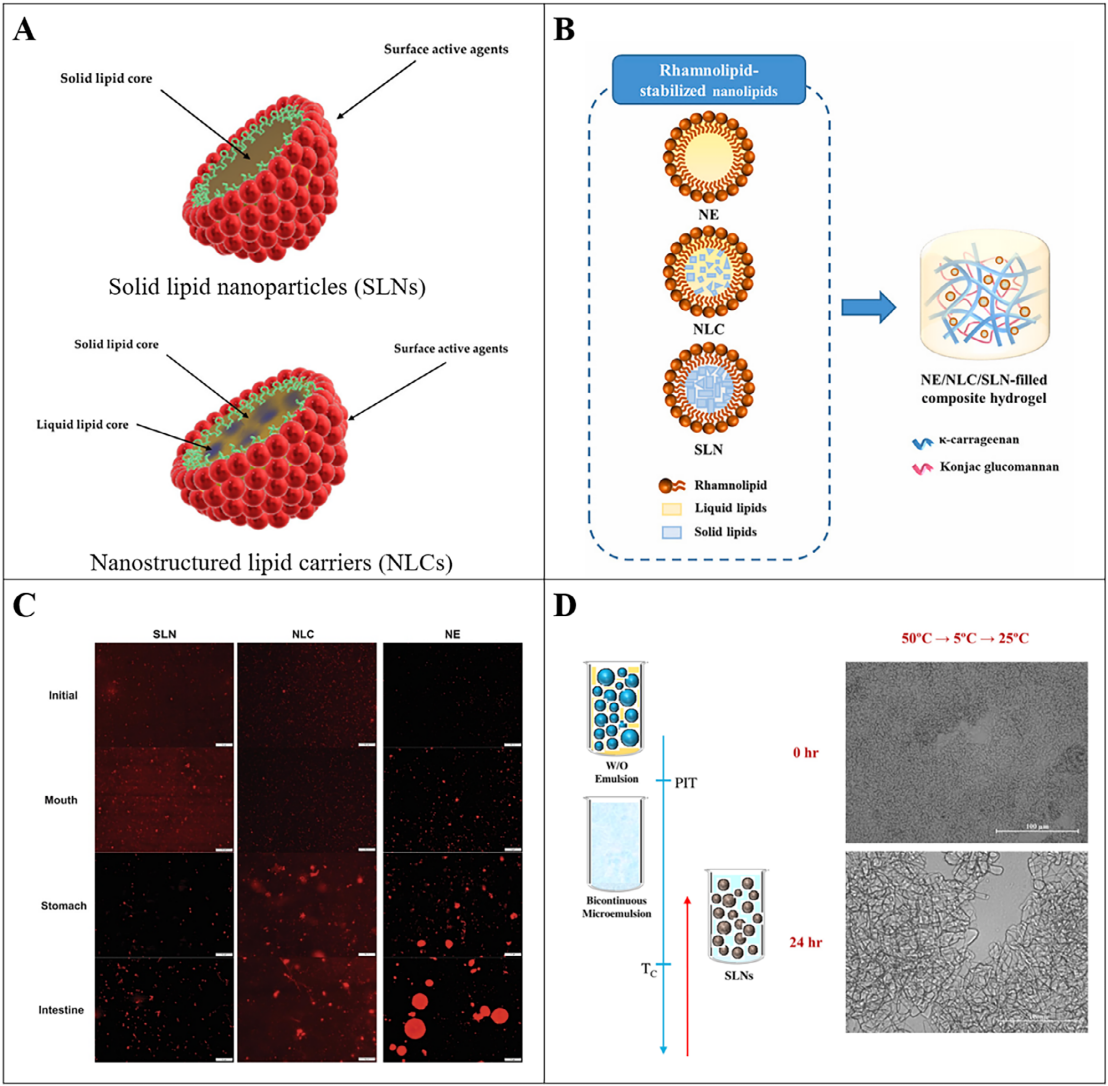
A) Schematic diagram of the structure of solid lipid nanoparticles (SLN) and the structure of nanostructured lipid carriers (NLC); Reproducted with permisson [116]. Copyright 2021, MDPI; B) Schematic diagram of improving lutein delivery efficiency by introducing stable rhamnose lipid nanoemulsion (NE)/NLC/SLN into κ‐carrageenan/konjac glucomannan (KC‐KGM) composite hydrogel; Reproducted with permisson [123]. Copyright 2024, Elsevier; C) Fluorescence microscopy images of curcumin‐loaded nanostructures (SLN, NLC, and NE) after each in vitro gastrointestinal digestion phase; Reproducted with permisson [124]. Copyright 2021, Elsevier; D) Left: Schematic diagram of the formation of solid lipid particles by phase transition temperature (PIT) methods; Right: Optical microscopy images of SLNs (C12E4/octadecane/water systems) taken at 0 h or after 24 h storage, which indicated that the system consisted of large irregularly shaped structures; Reproducted with permisson [128]. Copyright 2016, Elsevier.

Solid lipid nanoparticles (SLNs) and nanostructured lipid carriers (NLCs) are lipid‐based colloidal delivery systems used for the encapsulation of phytochemicals (Figure 2C). SLNs are the type of nanosphere that consists of solid lipids particle sizes ranging from approximately 50 to 500 nm (Figure 5A) [115, 116]. SLNs combine the advantages of liposomes, emulsions, and polymeric nanoparticles, which provide the stability of a solid matrix and the biological compatibility of a lipid carrier, avoiding the limitations associated with these delivery systems [117]. NLCs have the colloidal structure composed of a mixture of solid and liquid lipids, forming an amorphous lipid matrix surrounded by a solid lipid coating (Figure 5A) [116]. The combination of solid and liquid lipids provides structural integrity to NLC, with less tissue structure, thereby having more stable encapsulation in the lipid matrix [118].

In SLNs and NLCs systems, small lipid particles are either fully or partially solidified [119]. Due to the inhibited migration of bioactive substances in oil droplets fully or partially solidify, SLNs and NLCs tend to have high potentials to improve the stability and bioaccessibility of phytochemicals. SLNs and NLCs have been widely used to encapsulate hydrophobic phytochemicals such as β‐carotene [120, 121], lycopene [122], lutein (Figure 5B) [123], curcumin [124], and so on. These delivery systems also showed various advantages including increased loading capacity, enhancement of stability, and improved bioavailability [125]. For instance, NLCs encapsulated with curcumin were demonstrated to have better particle's stability during in vitro digestion and stronger intestinal permeability when compared with nanoemulsions, and higher curcumin's bioaccessibility when compared with SLNs. (Figure 5C) [124]. Zeaxanthin was loaded to produce SLNs and NLCs using high shear force and ultrasound technique and NLCs were shown to have a higher loading efficiency for zeaxanthin than SLNs [126].

SLNs have some potential drawbacks related to the crystallization of lipid phase which can lead to bioactive compounds expulsion during storage and the polymorphic transition of the lipid phase that may cause the lipid particles to become non‐spherical, thereby promoting their aggregation (Figure 5D) [125, 127, 128]. The combination of saturated and unsaturated fatty acids in NLCs results in a lower degree of crystallization and greater fatty acid chain spacing, which facilitates the incorporation of bioactive compounds and reduces expulsion during polymorphic transitions, effectively overcoming the possible limitations of SLNs [129]. It should be noted that the selection of the lipids should be very careful in view of maintaining good storage stability of the solid forms of the nanoparticles. For food applications involving thermal processing, proper formulated process of SLNs and NLCs should be adopted to ensure their desirable functional and textural properties at the temperatures used.

### 3.4 Biopolymer‐Based Systems

Many biomacromolecules, such as proteins and polysaccharides, can assemble into various colloidal delivery systems, such as biopolymer‐based particles, microgels, and inclusion complexes (Figure 2D). The encapsulation and retention of phytochemicals in these systems typically depend on various physical interactions, such as hydrogen bonding, hydrophobic attraction, van der Waals, and so on.

**FIGURE 6:**
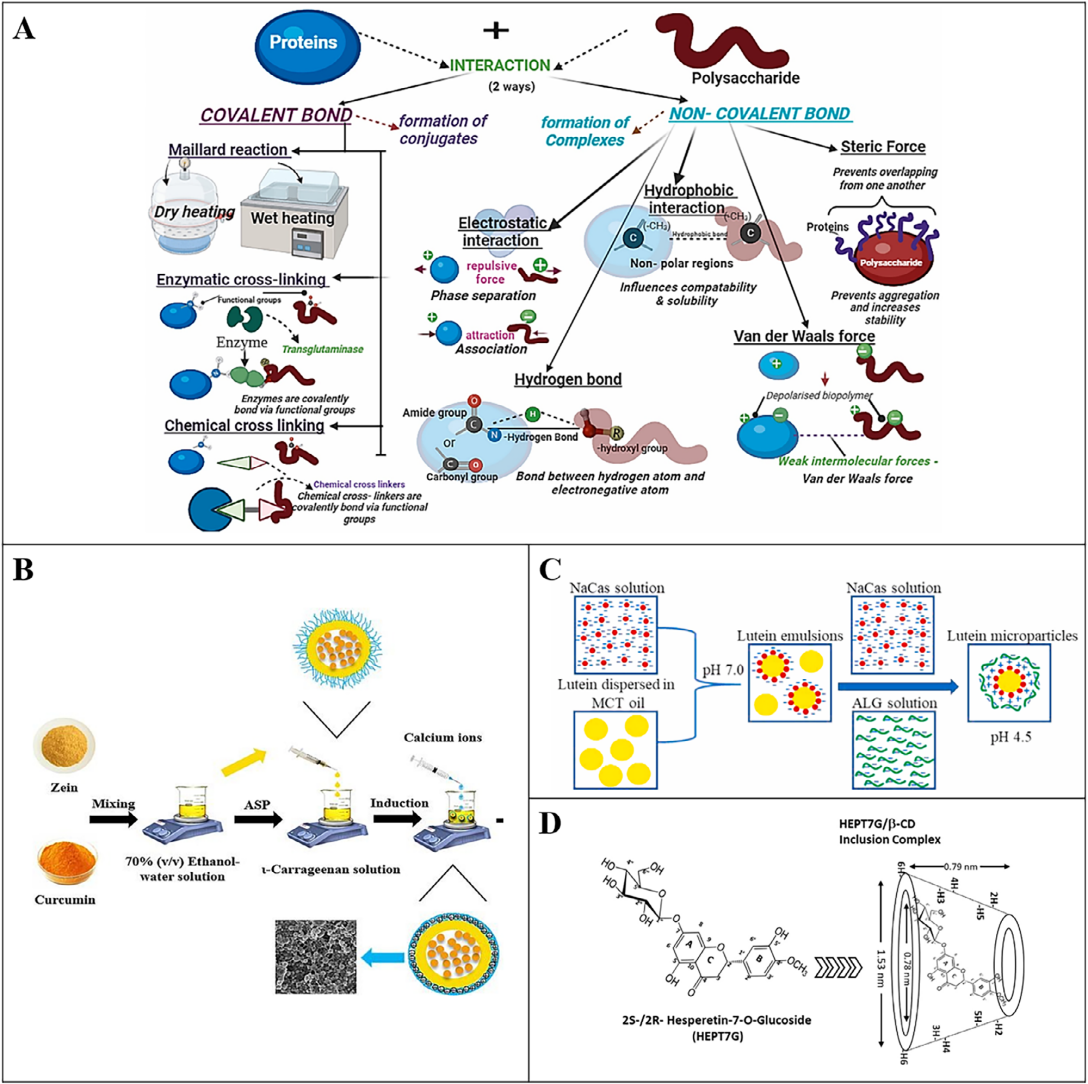
A) Interaction of protein polysaccharide through covalent and noncovalent interaction; Reproducted with permisson [130]. Copyright 2024, Elsevier; B) Calcium ions induced ι‐carrageenan‐based gel‐coating deposited on zein nanoparticles for encapsulating the curcumin; Reproducted with permisson [132]. Copyright 2024, Elsevier; C) Schematic diagram of the fabrication of lutein emulsions and lutein microparticles: lutein emulsions prepared by sodium caseinate (NaCas) and 2.0 wt% of lutein dispersed in medium‐chain triglyceride (MCT) oil, and lutein microparticles prepared by mixing emulsions with sodium caseinate (NaCas) solution and sodium alginate (ALG) solution; Reproducted with permisson [133]. Copyright 2022, Elsevier; D) Schematic illustration describing the conceptualization of the enantiomeric hesperetin‐7‐*O*‐glucoside (HEPT7G) inclusion into truncated cone structured hydrophobic cavity of β‐cyclodextrin (β‐CD) for the proprietary HEPT7G/βCD inclusion complex formulation; Reproducted with permisson [140]. Copyright 2023, Elsevier.

Proteins and/or polysaccharides can form a verity of micro or nanoparticle systems through the agglomeration effects, which can be induced through nucleation and crystal growth mechanisms or by protein denaturation and hydrophobic interaction (Figure 6A) [17, 130]. These biopolymer‐based particles have been widely used to encapsulate phytochemicals to improve their functional performance. Encapsulation of EGCG in a nanocomplex composed with caseinophosphopeptides (CPP) and chitosan (CS) showed no damage to the antioxidant activity of EGCG. Furthermore, the CS‐EGCG‐CPP nanocomplexes exhibited stronger protective effects against H_2_O_2_‐induced oxidative damage and more significant anti‐inflammatory activities in RAW264.7 cells compared with the free EGCG [131]. A carrageenan‐coated zein nanoparticle system was applied to encapsulate curcumin, which enhanced its heat and light stability. In addition, the encapsulation improved the stability of curcumin in GI tract, which may be beneficial for its bioactivity and health‐promoting effects (Figure 6B) [132]. Encapsulating lutein in composite microparticles fabricated from sodium caseinate and sodium alginate enhanced its bioaccessibility in a simulated GIT model and its chemical stability during long‐term storage (Figure 6C) [133].

Biopolymer microgels consist of small particles (typically 100 nm to 1000 µm) containing a three‐dimensional (3D) porous network of crosslinked proteins and/or polysaccharides contain relatively large amounts of water compared with the nanoparticles discussed above. This type of delivery system has considerable potential for their ability to encapsulate, protect, and release bioactive components [134]. Cold‐renneted milk protein concentrate was applied as composite materials for alginate gels, which showed increased encapsulation efficiency and decreased release rate of polyphenols in simulated gastric fluid [135]. The particle‐filled microgels based on zein and carboxymethyl starch were prepared to deliver quercetin, in which the anti‐solvent precipitation and chemical cross‐linking methods were applied. The microgels showed a high encapsulation rate of quercetin. In addition, the storge and light stability of quercetin was enhanced in this delivery system. Furthermore, the transport efficiency of quercetin in Caco‐2 cell monolayers and sustained release of quercetin in simulated intestinal fluids were all improved [136]. It is worth noting that it is important to design the appropriate delivery vehicle for the specific applications, which should try to avoid the loss of bioactivity of phytochemicals and mask the unpleasant tastes or odors [73]. Moreover, the relatively low loading contents of bioactive components may be the obstacle to the further in vivo applications of the delivery systems.

The inclusion complexes can be formed by “host” molecules entrapping “guest” phytochemical molecules through noncovalent interactions, such as hydrophobic attraction, hydrogen bonding, or van der Waals [14]. Cyclodextrins [137] and debranched starch [138] are all food‐grade “host” molecules. There are three principal cyclodextrins: α‐cyclodextrin (α‐CD), β‐cyclodextrin (β‐CD), and γ‐cyclodextrin (γ‐CD), among which the β‐CD is the most common host molecule. Due to the medium cavity size, β‐CD can complex a wide range of hydrophobic compounds with a high entrapment efficiency [139]. β‐CD inclusion complexes have been widely utilized to improve the oral bioavailability of hydrophobic phytochemicals without the use of lipids, co‐solvents, or surfactants. For instance, an inclusion complex of hesperetin‐7‐*O*‐glucoside with β‐CD (HEPT7G/βCD) has been prepared, in which the stability of hesperetin glucoside moiety was significantly enhanced during digestion. The bioavailability of HEPT7G/βCD was 12.1‐fold higher compared with α‐monoglucosyl hesperidin in SD rats and 104‐fold higher compared with hesperidin in humans (Figure 6D) [140]. Tea polyphenols were encapsulated in a sulfobutyl ether cyclodextrin‐based inclusion complex, in which the antioxidant activity, α‐glucosidase scavenging ability, and thermal stability of tea polyphenols were all improved [141]. In a word, cyclodextrin is a kind of useful “wall material” to encapsulate multiple phytochemicals and improve the solubility, antioxidant activity, and release of guest molecules. However, chemical derivations are common for cyclodextrins which may bring about the concerns of food safety, and the relative high cost of cyclodextrin preparation may also reduce its commercial feasibility in food industrial.

### 3.5 Hydrogels

**FIGURE 7:**
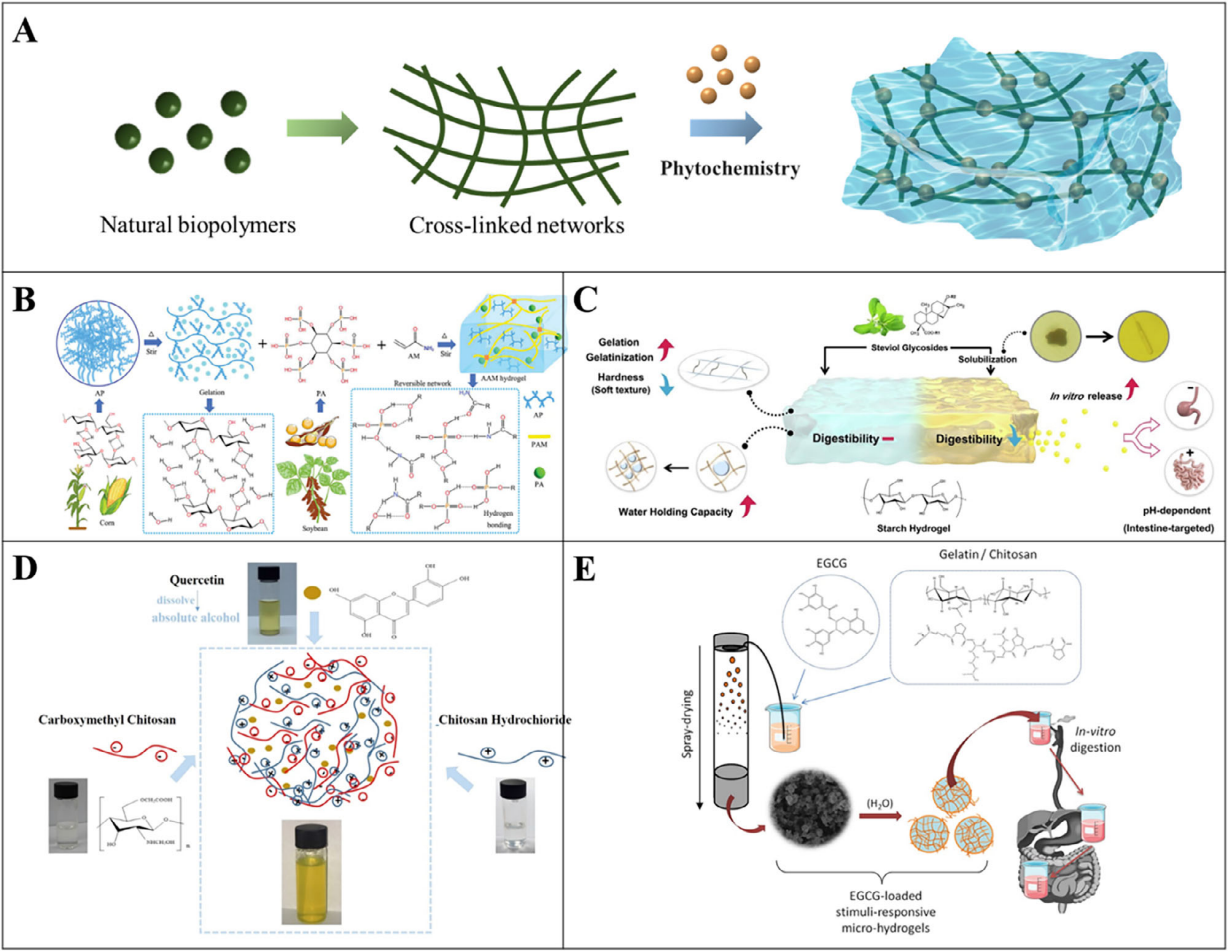
A) Schematic illustration of the formation of food‐grade hydrogels by natural biopolymers; B) Schematic illustration of the preparation process of amylopectin (AP) /polyacrylamide (PAM) hydrogels with different amounts of acrylamide (AM); Reproducted with permisson [144]. Copyright 2025, Wiley; C) Schematic diagram showing that adding stevioside promotes starch gelatinization and gelation and increases the release rate of curcumin; Reproducted with permisson [146]. Copyright 2024, Elsevier; D) Quercetin‐loaded chitosan hydrochloride (CHC) and carboxymethyl chitosan (CMCN) were prepared into nanoparticles through electrostatic interactions to improve the bioavailability of quercetin in functional foods and dietary supplements; Reproducted with permisson [149]. Copyright 2018, Elsevier; E) Two different hydrogel‐forming biopolymers (gelatin and chitosan) were compared as wall materials for the microencapsulation of a model flavonoid, (−)‐epigallocatechin gallate (EGCG); Reproducted with permisson [151]. Copyright 2016, Elsevier.

Hydrogels are materials that contain 3D cross‐linked networks of polymer chains capable of absorbing and retaining a considerable amount of water in the substitutional spaces between chains (Figure 2E). Hydrogel technologies have been widely applicated in various regions including hygienic products, drug delivery systems, sealing, diagnostics, and food additive [142]. In recent years, applying hydrogel as a carrier of Phytochemistry delivery system has gained increased attentions (Figure 7A). Natural biopolymers such as proteins and polysaccharides are ideal materials for the production of food‐grade hydrogels. These natural hydrogels are highly biocompatible and biodegradable [143].

Starch is a kind of abundant polysaccharides obtained from renewable sources such as cereals and tubers. A physical cross‐linking network was constructed using phytate derived from soybeans as a hydrogen bond bridge, which improved the tensile strength of hydrogels based on linear starch from corn (Figure 7B) [144]. Starch‐based hydrogels have been extensively studied as encapsulation systems for phytochemicals. Bioactive molecules can be uniformly encapsulated in starch‐based hydrogel systems, in which the release behavior of molecules is easily controlled by the external environment [145]. For instance, the addition of stevioside promotes the gelatinization and gelation of starch, enhancing the water retention capacity of the final product, which also promotes the release rate of curcumin (Figure 7C) [146]. A curcumin emulsion was placed in a 4‐α‐glucanotransferase‐treated rice starch‐based hydrogel, after which the UV stability and retention of curcumin after in vitro digestion of the filled hydrogels samples were improved [147]. CS is a natural cationic polysaccharide that presents a lot of interest in hydrogels. CS can form hydrogels through covalent bonds, hydrogen bonds, electrostatic and hydrophobic interactions alone or together with other ingredients, thereby serving as an effective phytochemicals carrier to improve its bioavailability [148]. Quercetin was encapsulated in a CS hydrochloride and carboxymethyl CS‐based composite hydrogel system, in which the chemical stability, solubility, and bioavailable property of quercetin were improved (Figure 7D) [149]. In addition, pH‐responsive CS‐based hydrogels have become a popular delivery vehicle due to their ability to release under different pH conditions, thereby achieving targeted release in GIT [150]. Gelatin was applied as a wall material to encapsulate EGCG in a protein‐based hydrogel system. Compared with a CS‐based hydrogel system, the gelatin‐hydrogel exhibited a higher bioaccessibility of EGCG after in‐vitro GI digestion (Figure 7E) [151]. Moreover, encapsulation of EGCG in hydrogels also hindered its dimer formation during in‐vitro digestion, thus suggesting greater bioavailability when compared with free EGCG [151]. Other phytochemicals, such as resveratrol [152], anthocyanin (Blueberry anthocyanins) [153], lutein [154], and apigenin [155], were also encapsulated in hydrogel‐based systems, which enhanced their stability and functional properties.

Food‐grade hydrogels have been widely used and studied as delivery systems with great potential to enhance the stability and bioavailability of bioactive phytochemicals. However, a couple of important questions are still unclear yet, including the intrinsic interaction mechanisms between food hydrogels and other food components, the interaction between food hydrogel and body, the development of functional foods based on food hydrogel that meet the requirements of specific groups (such as the patients with obesity, diabetes, and cardiovascular diseases), and the safety of food hydrogel [156].

### 3.6 Cage‐Like Proteins

**FIGURE 8:**
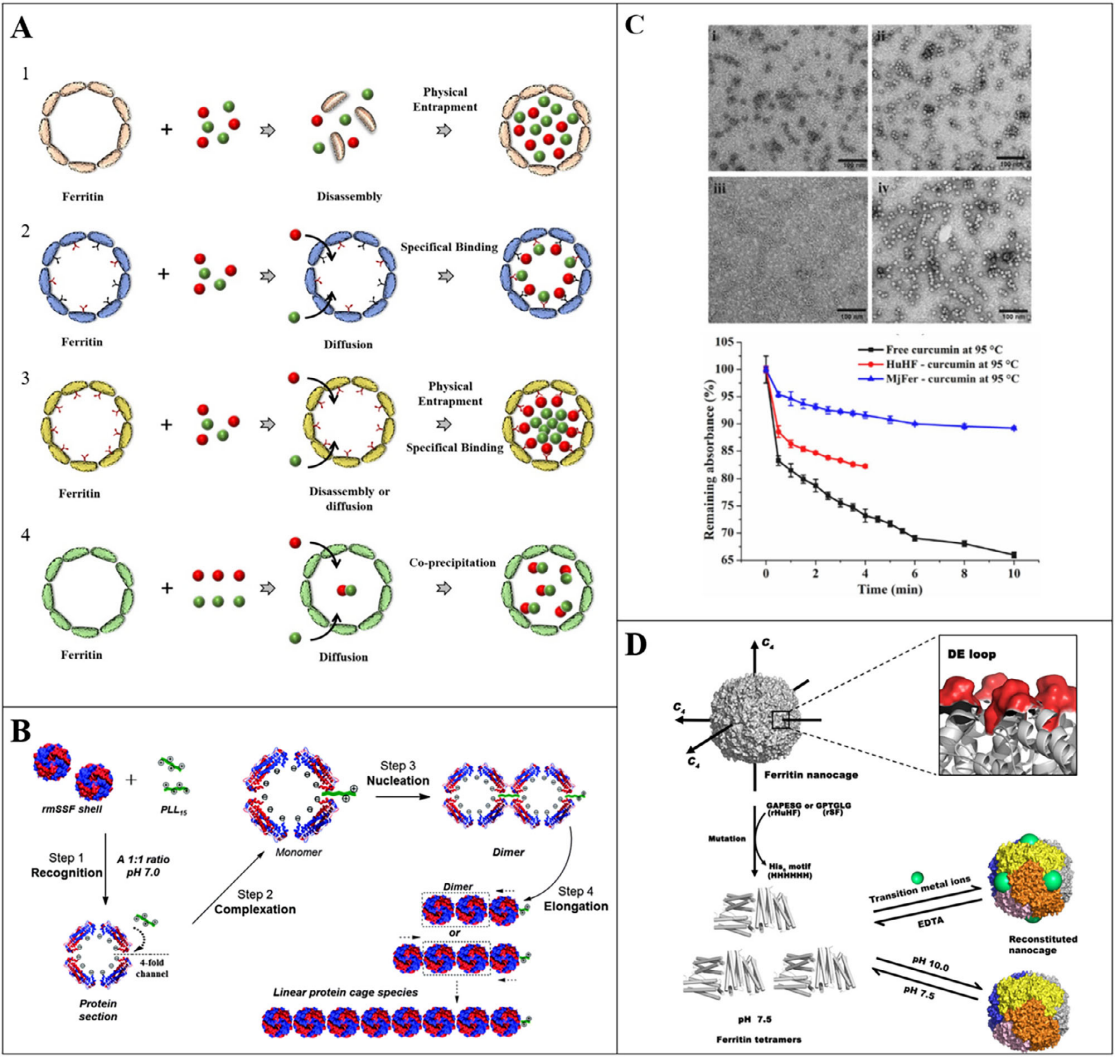
A) The illustration of co‐encapsulation of different types of cargos within ferritin nanocages. (1) Physical entrapment of different cargos within ferritin cavities by using the reversible disassembly and reassembly properties; (2) Specific binding of different types of cargos over the inner surface of ferritin nanocages; (3) Physical entrapment combined specific binding for co‐encapsulation of different types of cargos. (4) Co‐precipitation‐mediated co‐encapsulation of different cargos; Reproducted with permisson [158]. Copyright 2022, Elsevier; B) A schematic representation of the formation of a reconstructed mature soybean seed ferritin (rmSSF) linear assembly induced by poly(α, l‐lysine) (PLL15); Reproducted with permisson [159]. Copyright 2022, Royal Society of Chemistry; C) TEM images of curcumin encapsulated by different ferritins: (i) Apo human H‐chain ferritin (HuHF) without curcumin, (ii) Curcumin‐loaded HuHF, (iii) Apo *Marsupenaeus japonicus* ferritin (MjFer) without curcumin, (iv) Curcumin‐loaded MjFer. Below: Kinetic curves of MjFer‐curcumin, HuHF‐curcumin, and free curcumin heated at 95 °C for 10 min; Reproducted with permisson [164]. Copyright 2021, Elsevier; E) Schematic description of the design and His‐mediated self‐assembly property of a type of non‐native protein by using recombinant human H‐chain ferritin (rHuHF) and recombinant shrimp (*Marsupenaeus japonicus*) ferritin (rSF) as starting materials, which was created by mutating six amino acid residues (GAPESG for rHuHF and GPTGLG for rSF) located at the DE‐loop of each ferritin subunit nearby the *C*_4_ interfaces into HHHHHH (His_6_). The created non‐native protein naturally occurs as tetramers in solution at pH 7.5, which are able to reassemble into protein nanocages in response to transition metal ions or alkaline pH. The reconstituted nanocages can be dissociated into their tetramer analogues likewise in response to multiple external stimuli; Reproducted with permisson [166]. Copyright 2020, American Chemical Society.

Cage‐like proteins are native state proteins that self‐assemble into a 3D structure characterized by a hollow cavity (Figure 1F), thereby exhibiting the ability to encapsulate phytochemicals within its native cavity [157]. In addition, the easy modification of the outer surface provides an opportunity to enhance the stability of nanocages in the GI environment and achieve targeted delivery, resulting in better performance as nanocarriers (Figure 8A) [157, 158]. Coupled with the biocompatibility, monodispersity, and high thermal stability of the proteins, there is no doubt that cage‐like proteins are a class of superior nanocarriers.

Examples of proteins exhibiting nanocage‐like structures include ferritin (Figure 8B) [159, 160], heat shock proteins [161], and DNA‐binding proteins [162]. Among them, ferritin is the most popular and widely used nanocarrier for encapsulating and delivering phytochemicals. Based on the pH‐regulated reversible assembly properties, the apo‐form ferritin nanocage from various plant and animal matrices have been used to encapsulate water‐soluble and water‐insoluble phytochemicals, such as anthocyanin (cyanidin‐3‐*O*‐glucoside) [163], lycopene, rutin, lutein, curcumin, *β*‐carotene, EGCG, chlorogenic acid, paclitaxel, quercetin, and so on [158]. By isolating them from the outer environment using the cage‐like proteins, the stability, solubility, and bioavailable property of phytochemicals were greatly improved. For instance, *Marsupenaeus japonicus* ferritin can protect curcumin molecules from damage caused by thermal treatment at high temperatures up to 95 °C (Figure 8C) [164]. In the ongoing research on ferritin encapsulation, a significant amount of effort has been invested in changing the structure of ferritin to make it disrupted in mild environment rather than the unextreme pH conditions currently. A series of methods including chemical, physical, genetic modification, and channel expansion have been developed to realize encapsulation under relatively benign conditions [165]. With the use of pH‐sensitive amino acids, histidine, the higher encapsulation efficiency of curcumin was achieved under mild conditions, which was 3.6 times higher than that in the conventional acid denatured encapsulation (Figure 8D) [166]. However, from the perspective of encapsulation, compared with emulsions and hydrogels, the loading efficiency of phytochemicals in cage‐like proteins needs to be greatly improved.

## 4 Tradeoff Between Effective Health Promoting Functions and the Appreciate Appearance of the Final Food Products

To certain extent, one of the most complex examples of SCM with which we interact daily is foods [167]. Food products are generally presented in the forms of soft condensed matter, such as emulsions, gels, coacervates, solid blocks, and so on, with well‐defined shape, endowing them with the texture, touchness, tasty, mouth‐filling those satisfy the enjoy of eating. Shaping of food products is usually relied on the biomacromolecules including proteins, polysaccharides, and lipids, as well as the interactions among them, resulting in the food matrix. Integrating high enough content of bioactive phytochemicals to food matrix homogenously is a big challenge. On one hand, as known from Table 1, substantially large amounts of phytochemicals are exactly required for them to exert the corresponding health promotion functions including regulation of gut microbiota. However, on the other hand, phytochemicals, even at low concentrations, are inclined to induce the disordered aggregation of biomacromolecules to form precipitates.

**TABLE 1:** Encapsulation of phytochemicals.

Encapsulation materials	Concentration of carriers	Encapsulated phytochemicals	Concentration of phytochemicals	Refs.
**Nanoparticle**				
Zein and chitosan	50.3 mg/mL	Resveratrol	0.3412 mg/mL	[249]
Zein and sodium alginate	5.2 mg/mL	Resveratrol	0.3681 mg/mL	[250]
Hydrogenated soybean lecithin, sorbitol and hydrogenated palm oil	60.5 mg/mL	Trans‐resveratrol	2.1035 mg/mL	[251]
Gelatin	50 mg/mL	Curcumin	4.85 mg/mL	[252]
Shea butter	76 mg/mL	Curcumin	0.5620 mg/mL	[253]
Poly(lactic‐*co*‐glycolic acid) and polyvinyl alcohol		Curcumin	150 mg/g nanoparticles	[254]
Polylactide–poly(ethylene glycol), cetyltrimethylammonium bromide		Curcumin	2.4575 mg/mL	[255]
Silk protein and pluronic F‐68 DMSO	6 mg/mL	Resveratrol	0.0758 mg/mL	[256]
Polyaspartic acid and chitosan	2 mg/mL	EGCG	0.25 mg/mL	[257]
1‐(palmitoyl)‐2‐(5‐keto‐6‐octene‐dioyl) phosphatidylcholines, (+)‐α‐tocopherol acetate, Kolliphor HS15 and soy phosphatidycholines	5.9276 mg/mL	EGCG	0.6935 mg/mL	[258]
poly(lactic‐*co*‐glycolic acid) and polyvinyl alcohol	12.5 mg/mL	Quercetin	241 mg/g nanoparticles	[259]
Inulin	20 mg/mL	Quercetin Vanillin	4.44 mg/g spray‐dried powders 13.5 mg/g spray‐dried powders	[260]
Chitosan	6 mg/mL	Rosmarinic acid Procatechuic acid 2,5‐dihydrobenzoic acid	4.74 mg/mL 2.70 mg/mL 1.83 mg/mL	[261]
Sugar beet pectin and whey protein isolate	41.67 mg/mL	Anthocyanin‐rich extract	0.312 mg/mL	[262]
Fine shellac powder and xanthan gum	7.75 mg/mL	Cinnamon bark powder	148.5 mg/mL	[263]
Corn starch, sodium alginate, and CaCl_2_	20, 20, and 5.549 mg/mL	Yerba mate extract	19.5 CA mg/g dry capsules	[264]
Monoolein, poloxamer 407, polyethylene glycol‐400, and Transcutol HP	1 mg/mL	Berberine	1 mg/mL	[265]
Poly (lactic‐*co*‐glycolic) (PLGA)	30 mg/mL	BBR dodecyl sulfate salt (BBR‐S)	34.2 mg/mL;	[266]
		BBR laurate salt (BBR‐L)	28.9 mg/mL	
Poly (lactic‐*co*‐glycolic) acid (PLGA)	—	Eugenol	88.8 mg/mL,	[267]
		Linalool	83.8 mg/mL	
		Geraniol	56.5 mg/mL	
**Nanocapsulate**				
Stearic acid and lecithin	12 mg/mL	Resveratrol	2.6176 mg/mL	[268]
Sorbitan monostearate, poly(ε‐caprolactone), grapeseed oil and polysorbate	56.2 mg/mL	Resveratrol Curcumin	0.49 mg/mL 0.49 mg/mL	[269]
Poly(ε‐caprolactone), sorbitan monoestearate, and polysorbate 80	56.2 mg/mL	Resveratrol Curcumin	0.49 mg/mL 0.50 mg/mL	[270]
2‐Hydroxypropyl β‐Cyclodextrin	11.168 mg/mL	Gallic acid 1‐hydrate Ferulic acid	1.5535 mg/mL 1.5105 mg/mL	[271]
Gelatin and ι‐Carrageenan	17.5 mg/mL	Gallic acid Catechin Chlorogenic Tannic acid	2.25 mg/mL	[272]
Sorbitan monostearate, poly(ε‐caprolactone) and capric/caprylic triglycerides	5.43 mg/mL	Caffeic acid Ferculic acid *р*‐Coumaric acid Sinapic acid	0.53 mg/mL 0.62 mg/mL 0.78 mg/mL 0.68 mg/mL	[273]
**Nanoliposome**				
Rapeseed lecithin	50 mg/mL	Apigenin	0.0322 mg/mL	[274]
Glycerol formal and soybean lecithin	150 mg/mL	Resveratrol	1.724 mg/mL	[275]
Egg yolk phosphatidylcholine	125 mg/mL	Curcumin and resveratrol	0.0049 mg/mL and 0.0006 mg/mL	[276]
l‐α‐phosphatidylcholine	5 mg/mL	Spent Coffee Grounds extract	21 CAE mg/g liposomes	[277]
Sunflower lecithin	10 mg/mL	Curcumin Quercetin hydrate Resveratrol	0.3632 mg/mL 0.4878 mg/mL 0.3378 mg/mL	[278]
Soy lecithin, cholesterol, and Tween‐80	14.2 mg/mL	Anthocyanins extracts	1.3770 mg/mL	[279]
Bovine milk phospholipid concentrate 700	100 mg/mL	Tea polyphenol	2.32 mg/mL	[280]
**Nanoemulsion**				
Whey protein isolate	10 mg/mL	Resveratrol	0.13 mg/mL	[281]
Soy lecithin, medium‐chain triglycerides oil		Curcumin	1.64 mg/g nanoemulsion	[282]
**Microgel**				
OSA‐modified corn starch *and shells microgels*	1 mg/mL	EGCG	0.1278 mg/mL	[283]
Starch beads	0.5 mg/mL	Proanthocyanidin EGCG Catechin Epicatechin	1.1058 mg/mL 0.6509 mg/mL 0.3774 mg/mL 0.3483 mg/mL	[284]
**Complexes**				
β‐cyclodextrin	0.1362 mg/mL	Catechin	0.0348 mg/mL	[285]
**Microparticle**				
Resistant maltodextrin	400 mg/mL	Narngin	24 mg/mL	[286]
Phosphatidylcholine and Tristearin	95.7 mg/mL	*Trans*‐resveratrol	12.1975 mg/mL	[287]
Chitosan	8.3 mg/mL	Olive leaf extract	4.083 GAE mg/mL	[288]
Inulin	9 mg/mL	Olive leaves extract	13.2 GAE mg/g powder	[289]
Sodium alginate and CaCl_2_	15 and 20 mg/mL	Stevia leaves extract Aronia pomace extract	2.586 GAE mg/g dry microparticles 1.017 GAE mg/g dry microparticles	[290]
**Microcapsulate**				
Chitosan	4.44 mg/mL	Olive stones extract	17.2359 GAE mg/g dry matter content	[291]
Carboxymethyl chitosan	15 mg/mL	*Cistus L*. extract	0.3 GAE mg/mL	[292]
**Powders**				
Fructans inulin‐type from Agave tequilana Weber var. azul (Agaven)	60 mg/mL	Blue corn grains extract	1.4917 GAE mg/g dry basis	[264]

**FIGURE 9:**
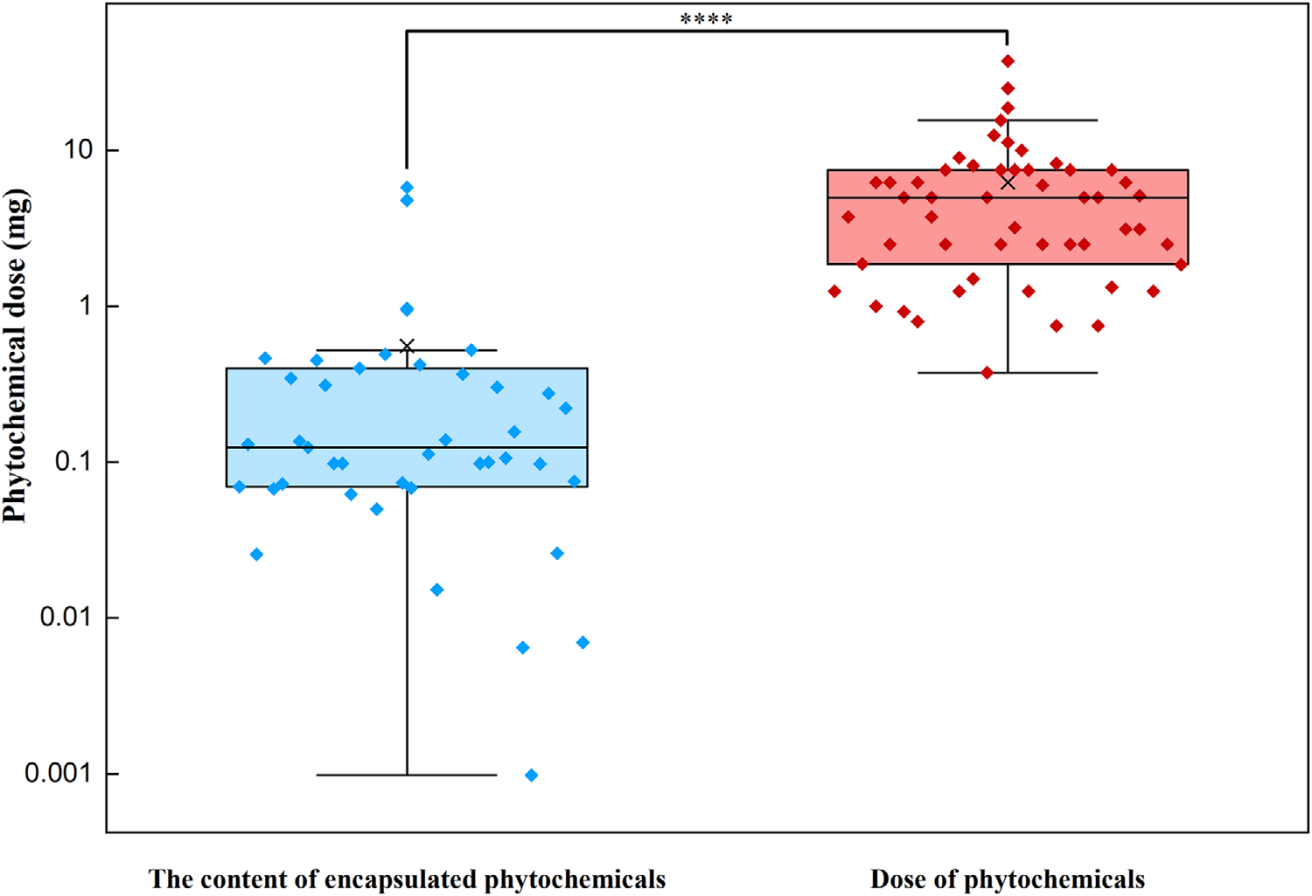
The content of phytochemicals in existing encapsulation technologies and the concentration of phytochemicals required to produce health‐promoting effects for alleviating chronic diseases (calculated based on an average mouse body weight of 25 g and a gavage dose of 200 µL). The data are presented as the means ± SDs. “*” represented significant differences (“****” *p* < 0.0001) as determined by *t*‐test.

The interaction between bioactive phytochemicals and the food biomacromolecules has been intensively investigated in the context of embedding the bioactive molecules into food‐grade matrixes with the length scales ranging from nanometer to micrometer. The phytochemicals such as resveratrol, curcumin, EGCG, quercetin, rosmarinic acid, protocatechuic acid, 2,5‐dihydrobenzoic acid, naringin, anthocyanins, and the polyphenol extracts were embedded and encapsulated with the polymeric matrixes including nanoparticles, nanocapsules, microparticles, microcapsules, and beads composed with food biomacromolecules (proteins and polysaccharides), or encapsulated with liposomes and emulsions (Table 1). The mechanisms involved in the interactions between the phytochemicals and proteins or polysaccharides have been well summarized referring to the recent review papers [17, 19, 168]. The content of phytochemicals in these encapsulation systems is generally lower than the dose required for the phytochemicals to exert the bioactivities (Table 2, Figure 9), which makes the development of templates with high‐phytochemical‐loading capacity even more essential.

**TABLE 2:** The health promotion effects of phytochemicals.

Type of phytochemicals	Animal model	Biological activity	Dose of phytochemicals	Refs.
**EGCG**	high‐fat diet (C57BL/6J male mice)	shift gut microbiota (promote intestinal bloom of *Akkermansia muciniphila*); regulate BA signaling	100 mg/kg	[293]
**EGCG**	nonalcoholic steatohepatitis (male C57BL/6J mice)	improve MCD‐diet‐derived gut microbiota dysbiosis; long‐chain‐fatty‐acid‐CoA ligase ACSBG played a distinct role in fatty acid metabolism and ferroptosis and was significantly negatively correlated with Bacteroides	50 mg/kg	[294]
**EGCG**	DSS‐induced colitis (male CF‐1 mice)	mitigate DSS‐induced colon shortening and spleen enlargement; colonic protein levels of IL‐1β, IL‐6, tumor necrosis factor‐α and colonic lipid peroxides↓; body weight↓	128 mg/kg	[295]
**resveratrol (RSV)**	high‐fat diet (C57BL/6J male mice)	blood cholesterol levels in a dose‐dependent manner↓(through enhancing TICE and limiting cholesterol absorption via selective activation of intestinal LXRα)	100, 500 mg/kg	[296]
**resveratrol (RSV)**	high‐fat diet (C57BL/6J male mice)	*Blautia* abundance↑; *Desulfovibrio* and *Lachnospiraceae_NK4A136_group* abundance↓; pathways linked to host metabolic disease↓; pathways involved in the generation of small metabolites↑	300 mg/kg	[297]
**resveratrol (RSV) and curcumin (Cur)**	high‐fat diet (C57BL/6J male mice)	Specific sulfur metabolism, SCFA metabolism and branched‐chain amino acids were mechanisms potentially responsible for the impact of resveratrol on glycemia. the Cur treatment was found to antagonize the action of RSV.	RSV: 60 mg/kg Cur: 30 mg/kg	[298]
**resveratrol (RSV)**	high‐fat diet (C57BL/6J male mice)	body weight and liver steatosis↓; improve insulin resistance; repair of intestinal mucosal morphology and increased the expression of physical barrier‐and physiochemical barrier‐related factors; abundance of harmful bacteria↓; abundance of short‐chain fatty acid (SCFA) ↑	300 mg/kg	[299]
**resveratrol (RSV)**	high‐fat diet (C57BL/6J male mice)	*Bacteroides*, *Lachnospiraceae_NK4A136_group*, *Blautia*, *Lachnoclostridium*, *Parabacteroides*, and *Ruminiclostridium_9*↑; RSV‐microbiota was able to modulate lipid metabolism, stimulate the development of beige adipocytes in WAT, reduce inflammation and improve intestinal barrier function.	300 mg/kg	[300]
**resveratrol (RSV)**	high‐fat diet (C57BL/6J male mice)	body weight gain, relative weight of liver and adipose tissue↓, alpha diversity of gut microbiota↑(Medium and high); prevent serum triglyceride, low density lipoprotein cholesterol, glucose, and endotoxemia; formation of malondialdehyde was prevented	50, 75, 100 mg/kg	[301]
**chlorogenic acid (CGA)**	high‐fat diet (male Wistar rats)	inflammation and fat deposition in the liver↓; plasma liver enzyme activities↓; diversity of gut microbiota↑	100 mg/kg	[302]
**theaflavin (TF)**	mice chronic toxicity test (female CD‐1 mice)	the capacity to remove the endogenous metabolic toxins through oxidation, amination, and MGO conjugation in the intestinal tract	200 mg/kg	[303]
**theabrownin**	high‐fat diet (C57BL/6J male mice)	the levels of ileal conjugated bile acids (BAs)↑; hepatic production and fecal excretion of BAs↑; hepatic cholesterol↓; lipogenesis↓	450 mg/kg (Pu‐erh tea)	[304]
**green tea polyphenols—Polyphenon E (PPE)**	mice chronic toxicity test (Female BKS db/db mice)	fasting blood glucose levels and mesenteric fat↓; the serum level of insulin↑	100 mg/kg	[41]
**Postfermented Pu‐erh tea (Polyphenols and caffeine (CAF))**	high‐fat diet (C57BL/6J male mice)	alter the gut microbial community structure and specific gut bacteria (*Akkermansia muciniphila* and *Faecalibacterium prausnitzii*); improve Diet‐Induced Metabolic Syndrome	750 mg/kg	[305]
**oil tea concentrated decoction**	diabetic C57BL/KsJ‐db/db (db/db) mice	the postprandial blood glucose elevation and the levels of FBG, total cholesterol, triglycerides, and LDL‐cholesterol↓; change the composition of gut microbiota (*Lachnospiraceae*↑)	4000 mg/kg	[42]
**Fuzhuan brick tea (FBT) extracts**	high‐fat diet (male C57BL/6Cnc mice)	ameliorate obesity, serum lipid parameters, blood glucose homeostasis, hepatic steatosis, adipocyte hypertrophy, and tissue inflammation; restore *Firmicutes/Bacteroidetes* ratio; abundances of beneficial bacteria *Clostridiaceae*, *Bacteroidales*, and *Lachnospiraceae*↑; harmful *Ruminococcaceae*, *Peptococcaceae*, *Peptostreptococcaceae*	100, 200, 400 mg/kg	[28]
**decaffeinated green tea (GT) and black tea (BT) polyphenols**	high‐fat diet (C57BL/6J male mice)	cecum Firmicutes↓; Bacteroidetes↑; induce hepatic 5′adenosylmonophosphate‐activated protein kinase (AMPK) phosphorylation↑	GT: 240 mg/kg BT: 320 mg/kg	[306]
**cornstarch‐tea powder**	diabetes model (female Kunming mice)	blood glucose↓; levels of *Coriobacteriaceae*, *Lactobacillaceae*, *Prevotellaceae* and *Bifidobacteriaceae*↑; *Bacteroidaceae*, *Ruminococcaceae*, *Helicobacteraceae* and *Enterobacteriaceae*↓;	330 mg/kg	[40]
**extracts of raw or ripe Pu‐erh teas**	high‐fat diet (male Wistar rats)	the relative abundances of *Firmicutes*↑; the relative abundances of *Bacteroidetes*↓; microbial diversity↑; change the composition of cecal microbiota	150, 400 mg/kg	[307]
**proanthocyanidins (PAs) from grape seed extract (GSE)**	high‐fat diet (female Wistar Kyoto rats)	many microbial metabolites↓; the formation of conjugated (epi)catechin metabolites	30 mg/kg	[29]
**concord grape polyphenols (GP) powder**	high‐fat diet (C57BL/6J male mice)	weight gain, adiposity, serum inflammatory markers and glucose intolerance↓; intestinal expression of genes involved in barrier function (occludin) and limiting triglyceride storage↑; intestinal gene expression of proglucagon↑; the growth of *Akkermansia muciniphila*↑; the proportion of *Firmicutes* to *Bacteroidetes*↓	1×10^4 ^mg/kg	[308]
**GP extract (GPE) or grape proanthocyanidins (PAC)**	high‐fat diet (C57BL/6J male mice)	induce a bloom of fecal and cecal A. muciniphila	360 mg/kg	[30]
**table grapes powder**	high‐fat diet (C57BL/6J male mice)	liver weights, triglyceride levels, and expression of glycerol‐3‐phosphate acyltransferase (Gpat1) ↓(5%); the intestinal abundance of sulfidogenic *Desulfobacter spp*, and the *Bilophila wadsworthia*‐specific dissimilatory sulfite reductase gene (dsrA‐Bw)↓; the abundance of the beneficial bacterium *Akkermansia muciniphila*↑(3%); attenuate the HF‐induced impairment in epithelial localization of the intestinal tight junction protein zonula occludens	3%: 500 mg/kg 5%: 1500 mg/kg	[309]
**grape seed proanthocyanindin extract (GSPE)**	high‐fat diet (C57BL/6J male mice)	plasma levels of inflammatory factors↓; ameliorate macrophage infiltration in epidydimal fat and liver tissues; epidydimal fat mass↓; insulin sensitivity↑; modulate the gut microbiota composition and certain bacteria	300 mg/kg	[36]
**blueberry proanthocyanidins and anthocyanins powder**	high‐fat diet (C57BL/6J male mice)	body weight↓; insulin sensitivity↑	300, 4200 mg/kg	[38]
**cranberry extract (CE)**	high‐fat diet (C57BL/6J male mice)	weight gain and visceral obesity↓; liver weight and triglyceride accumulation↓; improved insulin sensitivity; the proportion of the mucin‐degrading bacterium Akkermansia↑	200 mg/kg	[37]
**wild blueberry polyphenolic extract (anthocyanins (F1), PACs oligomers (F2), and PACs polymers (F3))**	high‐fat diet (C57BL/6J male mice)	proportion of *Adlercreutzia equolifaciens* and *Akkermansia muciniphila*↑; restore colonic epithelial mucus layer	F1: 32 mg/kg F2: 53 mg/kg F3: 37 mg/kg	[32]
**chokeberry (*Aronia melanocarpa* L.) extracts**	high‐fat diet (male Wistar rats)	*Firmicutes*/*Bacteroidetes* ratio↓; the relative abundance of *Bacteroides*, *Prevotella*, *Akkermansia* and other bacterial species↑; reduce the body weight of HFD‐fed rats by accelerating energy homeostasis and thermogenesis in iBAT; improved dyslipidemia	1000 mg/kg	[33]
**cranberry extract (CRX) in combination with isomalto‐oligosaccharides (IMOs)**	high‐fat diet (male Swiss albino mice)	cecal SCFAs↑; gut beneficial bacterial abundance, gut histology and related changes↑; prevent HFD‐induced systemic and tissue inflammation, glucose intolerance and systemic obesity‐associated metabolic changes in adipose tissue and liver	200 mg/kg (or in combination with IMOs (1 g/kg))	[31]
**blueberry polyphenol extract (PPE)**	high‐fat diet (C57BL/6J male mice)	inhibit body weight gain and returned lipid metabolism to normal; change the composition of the gut microbiota	200 mg/kg	[35]
**apple polyphenols extract (APE) and phloretin**	DSS‐induced UC (male C57BL/6 mice)	both: ameliorate DSS‐induced UC by inhibiting body weight loss, preventing colon shortening and mucosa damage; APE: inhibit activation of NF‐κB signaling; hyodeoxycholic acid level↓; abundance of *Verrucomicrobia* at phylum and *Bacteroides* and *Akkermansia* at genus↑; β‐muricholic acid level↑; Bacteroidetes abundance↓; lower disease activity index score, less body weight loss and lighter spleen phloretin: Firmicutes abundance↓	APE: 125 or 500 mg/kg phloretin: 100 mg/kg	[48]
**apple polyphenol extract (APE)**	high‐fat diet (C57BL/6J male mice)	total fecal BA contents↓; relative abundance of Akkermansia↑; relative abundance of Lactobacillus↓; might affect the reverse cholesterol transport in the ileum	125 and 500 mg/kg	[34]
**flavanol**	humanized gnotobiotic mice, Alzheimer's disease (AD) (flavanol‐rich preparation)	bioactivity toward interfering with α‐synuclein misfolding or inflammation	40 mg/kg	[310]
**gallotannins (GT)**	high‐fat diet (GF C57BL/6J mice)	GT and colonized *L. plantarum* reduce HFD‐induced inflammation, insulin resistance, and promote adipose tissue thermogenesis	50 mg/kg	[311]
**lonicera caerulea L. polyphenols (LCPs)**	high‐fat diet (male Sprague‐Dawley rats)	enhance intestinal permeability; levels of pro‐inflammatory cytokines↓; alleviates LPS‐induced liver injury by suppressing the nuclear translocation of NF‐κB p65 and activation of the MAPK signaling pathway; alter the composition of the intestinal microbiota	250 mg/kg	[312]
**pomegranate (Punica granatum L.) polyphenolics**	DSS‐induced colitis (Sprague–Dawley rats)	the expression of p70S6K1 and HIF1α↓; induce miR‐145 in a dose‐dependent manner	499.27 mg GAE/kg (expressed as mg ellagic acid equivalents/L)	[49]
**polyphenol of Chinese propolis (CP) and Brazilian propolis (BP)**	DSS‐induced colitis (Male Sprague Dawley rats)	colitis disease activity index relative↓; malonaldehyde levels↓; increased T‐AOC levels↑; IL‐1, IL‐6 and MCP‐1↓; populations of *Bacteroides* spp↓	300 mg/kg	[51]
**proanthocyanidins (GSPE)**	intestinal dysfunction induced with LPS (male Wistar rats)	normalization of OVA levels in vivo; MPO and COX‐2 activities↓; modulates the ileum inflammatory and permeability proteins gene expression; prevents increase of ROS levels	75 and 37 5 mg/kg	[55]
**nobiletin**	high‐fat diet (male Sprague‐Dawley rats)	the biotransformation activity was enhanced for both the host and gut microbiota; higher extent of demethylated metabolites in the plasma as well as in feces	100 mg/kg	[313]
**ouli fermented juice (OF)**	mice chronic toxicity test (male ICR mice)	improve host immunity and the intestinal mucosal barrier; serum immunoglobulin (Ig)A, IgG, and IgM levels and secreted IgA (sIgA) levels in the colon↑; the abundance of *Lachnospiracea*, *Roseburia*, *Akkermansia*, and butyric acid‐producing microbes↑; the levels of short chain fatty acids in the colon↑	625, 1250, and 2500 mg/kg	[314]
**polyphenol extracts from Lessonia trabeculate**	diabetic rats with high‐fat diet (male C57BL/6J rats)	blood glucose (FBG) levels, insulin levels↓; improve the lipid profile and antioxidant stress parameters; preserve the architecture and function of liver; recover short chain fatty acids (SCFAs) contents; regulate the dysbiosis of the microbial ecology	200 mg/kg	[44]
**polyphenol‐rich pomegranate peel extract (PPE)**	high‐fat diet (male Balb/c mice)	the serum level of cholesterol (total and LDL) induced by HF feeding↓; counteract the HF‐induced expression of inflammatory markers both in the colon and the visceral adipose tissue; cecal content weight and cecal pool of bifidobacteria↑	250 mg/kg	[315]
**polyphenol‐rich peach peel extract (PPE)**	high‐fat diet (female ICR mice)	inhibit lipid accumulation in HFD‐fed mice; regulate mRNA expression levels involved in lipid metabolism; the relative abundance of *Lactobacillus*, *Bacteroides, Lachnospiraceae, Prevotellaceae, Alloprevotella, Akkermansia, Roseburia*, and *Ruminococcus*↑	150, 300 mg/kg	[316]
**pea polyphenols**	high‐fat diet (female C57Bl/6 mice)	the hydrolyzed proanthocyanidin‐rich diet (hPA) reduce both weight gain and pathogen colonization; cooked pea seed coats have been shown to improve metabolic function and intestinal health compared to raw pea seed coat supplementation	74 mg/kg	[39]
**neohesperidin (Neo)**	high‐fat diet (C57BL/6J male mice)	restore gut barrier damage, metabolic endotoxemia, and systemic inflammation; the diversity of gut microbiota↑	50 mg/kg	[317]
**holly polyphenols (HP)**	a piglet model of lipopolysaccharide (LPS)‐induced intestinal injury	intestinal disaccharidase activities, mRNA expression of intestinal tight junction proteins↑; plasma proinflammatory cytokines↓; alter hindgut microbiota composition by enriching *Prevotella* and enhancing SCFA production following LPS challenge	250 mg/kg	[52]
**dicaffeoylquinic acids (DiCQAs) from Ilex kudingcha**	DSS‐induced colitis (male C57BL/6J mice)	alleviate colitis disease activity index, colon shortening, colonic tissue inflammation and pro‐inflammatory cytokines; the level of SCFAs↑; relative abundances of Odoribacter and Prevotella↑; relative abundances of Bacteroides, Parasutterella and Lachnospiraceae↓	400 mg/kg	[54]
**a. keiskei juice (AKJ)**	high‐fat diet (C57BL/6J male mice)	prevent weight gain, lowered fat accumulation, blood glucose, serum lipid levels, hepatic steatosis, and modulated the level of expression of genes involved in lipid metabolism in mice with obesity; ameliorate HFD‐dependent changes in the relative abundance of several taxa back to normal status	205 mg/kg	[318]
**pomegranate extract (PE) and its main microbiota‐derived metabolite urolithin‐A (UROA)**	DSS‐induced colitis (male Fisher rats)	both: inflammation markers (iNOS, cycloxygenase‐2, PTGES and PGE2in colonic mucosa); modulate favorably the gut microbiota; G_1_ to S cell cycle pathway was up‐regulated; PE: oxidative stress in plasma and colon mucosa↓; UROA: preserve colonic architecture	PE: 250 mg/kg UROA: 15 mg/kg	[50]
**trans‐resveratrol**	fecal samples from healthy volunteers	gut bacteria readily convert trans‐resveratrol; the gut microbiota can have a great effect on the bioconversion of dietary constituents	35 mg/d per man	[56]
**anthocyanins+ chlorogenic acid (CGA)**	randomized, double‐blind, placebo‐controlled crossover trial	bacterial strains from the genus *Bifidobacterium*↑	anthocyanins: 375 mg/d CGA: 127.5 mg/d	[58]
**grape pomace particle**	randomized, double‐blind, placebo‐controlled crossover trial	*Prevotella* and *Firmicutes* levels↓; miR‐222 levels↑	8000 mg/d per man	[319]
**juçara berry**	randomized, double‐blind, placebo‐controlled crossover trial	fecal acetate↑; abundance of *A. muciniphila*, *Bifidobacterium spp*., and *C. coccoides*↑	207.55 mg/d	[57]
**pomegranate extract (PE)**	randomized, double‐blind, placebo‐controlled crossover trial	plasma LBP↓; *Bacteroides*, *Faecalibacterium*, *Butyricicoccus*, *Odoribacter* and *Butyricimonas*↑; *Parvimonas*, *Methanobrevibacter* and *Methanosphaera*↓(PE‐D2)	D1: 450 mg/d PE (containing 164 mg phenolics) D2: 1800 mg/d PE (containing 656 mg phenolics)	[59]
**polyphenols from mango (Mangifera indica L.)**	IBD patient volunteers	the plasma levels of pro‐inflammatory cytokines↓; the abundance of *Lactobacillus* *spp, Lactobacillus. plantarum*, *Lactobacillus. reuteri* and *Lactobacillus. lactis*↑	95.18‐190.36 mg/d	[53]
**cranberry powder**	randomized, double‐blind, placebo‐controlled crossover trial	the abundance of *Firmicutes*↑; the abundance of Bacteroidetes↓; bacteria‐derived deoxycholic acid↑; acetate and butyrate in stool↓; attenuate the impact of the animal‐based diet on microbiota composition, bile acids, and SCFA	3×10^4^ mg/d per man	[320]
**strawberry and cranberry polyphenols (SCP)**	a parallel, double‐blind, controlled and randomized clinical trial	improve insulin sensitivity and prevent an increase in compensatory insulin secretion	36 mg/kg	[321]

Despite the considerable progress achieved by the above‐mentioned food‐grade delivery and encapsulation systems in improving the dispersibility, stability, and sensory compatibility of phytochemicals, these carrier‐based platforms are inherently constrained by limited loading capacity. In such systems, phytochemicals are typically incorporated as guest molecules through physical entrapment or adsorption within pre‐formed biopolymer or lipid matrices. Increasing phytochemical content often leads to disruption of the biopolymer network [169], phase separation, or uncontrolled aggregation [170], thereby compromising both structural stability and functional performance. Consequently, conventional delivery systems discussed in Section 3 generally struggle to achieve high phytochemical loading while maintaining desirable physicochemical properties.

In light of these limitations, an alternative delivery paradigm has recently emerged that departs from the traditional carrier–cargo model by exploiting the intrinsic molecular characteristics of phytochemicals to drive self‐assembly through noncovalent interactions [171]. In this approach, phytochemicals are no longer passive payloads but actively participate in the formation of ordered or semi‐ordered soft condensed structures, substantially increasing the fraction of bioactive components within the system.

### 4.1 Self‐assembly of Natural Small Molecular Phytochemicals to Dimers and Oligomers

**FIGURE 10:**
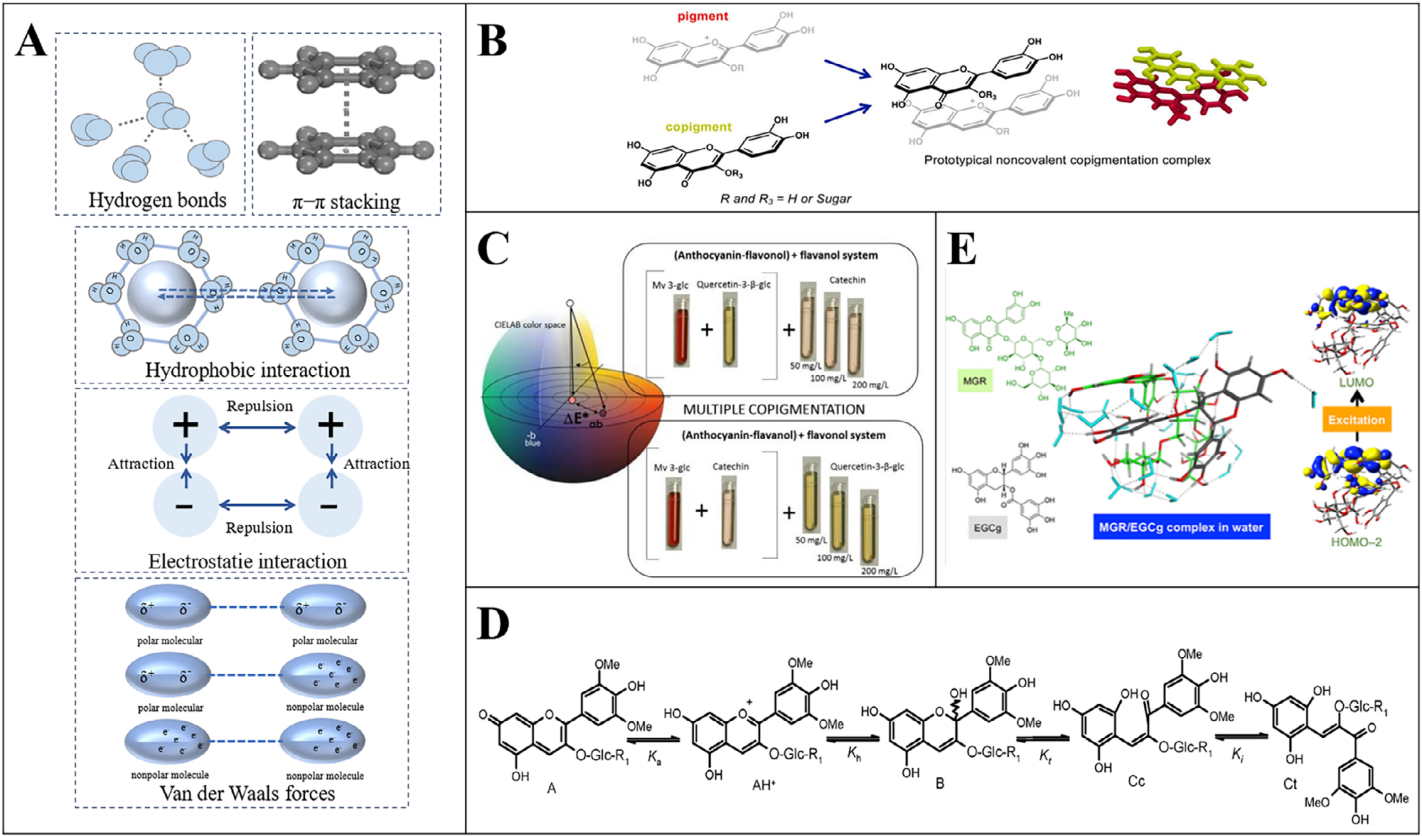
A) Self‐assembly mechanism of natural small molecular phytochemicals. B) Noncovalent association of a prototypical anthocyanin pigment and a prototypical flavonoid copigment; Reproduced with permission [173]. Copyright 2016, American Chemical Society. C) The combined effect of anthocyanin–flavanol–flavonol ternary interactions on the colorimetric and chemical stability of malvidin‐3‐glucoside; Reproduced with permission [176]. Copyright 2015, American Chemical Society. D) Network of the equilibrium forms of anthocyanins in the acidic medium; Reproduced with permission [177]. Copyright 2015, Royal Society of Chemistry. E) Complex structures of monoglucosylrutin (MGR) with *ent*‐gallocatechin‐3‐*O*‐gallate (*ent‐*GCg) and epigallocatechin‐3‐*O*‐gallate (EGCg) in aqueous solutions and the mechanism of color change induced by complexation; Reproduced with permission [180]. Copyright 2018, American Chemical Society.

Self‐assembly, which is common in nature, is defined as the spontaneous and reversible association of molecules or particles into organized structures by the weak interaction forces including hydrogen bonds, π–π stacking, hydrophobic forces, and electrostatic attraction (Figure 10A). The physical self‐assembly property of the phytochemical molecules exactly has been demonstrated to play important roles in the formation and protection of color in living plants as well as in the beverages originating from plants. The noncovalent complexation of phytochemicals can account for the subsequent changes in optical properties of the pigment, which is a well‐known color change phenomenon called copigmentation (Figure 10B) [172, 173]. Beautiful colors of many plants are greatly attributed to the phytochemical anthocyanins which change the color with varying pH. As it is hardly conceivable that flower cell sap could be alkaline, controversy about blue flower colors had lasted for a long period in history until the pigment–copigment assemblies were identified in blue and violet flowers, which is the manifestations of self‐assembly of small‐molecule phytochemical monomers into dimers [172, 173].

Not only in the color of plants, copigmentation has also been repeatedly confirmed in food beverages, with wine and tea by far the most studied ones. It is well‐known that the initial red purple color originating from the grapes is usually modified during winemaking and aging, in barrel or bottle. Anthocyanins serve as the core color carriers in wine, with their concentration and chemical transformations directly determining color intensity and hue [174]. From extraction and complexation during early fermentation to polymerization and stabilization during aging, anthocyanins consistently play a pivotal role. While total polyphenol content remains constant, the conversion of monomeric anthocyanins into polymeric pigments constitutes the intrinsic chemical mechanism underlying color evolution in aged wines [175]. All these compounds favor the occurrence of copigmentation at all stages of winemaking. Interestingly, a ternary copigmentation consisting of an anthocyanin (oenin) and two different flavonoids including (+)‐catechin and quercetin‐3‐β‐d‐glucoside has been suggested to be formed in wine when all three species are present at high concentrations (Figure 10C) [176]. Although they are only minor pigments in wine, acylated anthocyanins are more prone to self‐association than their nonacylated analogs (Figure 10D) [177].

Molecule self‐association similar to copigmentation but without the participation of anthocyanins were also reported in other beverages. Green tea catechins were found to form self‐assembled dimers in water, and gallate‐type catechins showed a greater tendency to self‐associate than nongallate‐type catechins. EGCG and epigallocatechin (EGC) were selected as the tea catechin representatives for investigating their complex formation with the other catechins [178]. EGCG showed rather strong binding affinities for other catechins; in contrast, the non‐gallate‐type catechin EGC exhibited generally low binding affinity for other catechins. Moreover, the higher binding abilities of gallate‐type catechins are further found to be due to providing multiple intermolecular interactions that remain effective in an aqueous environment, such as aromatic/aromatic or CH/π interactions [179]. It is interesting that the complex of monoglucosylrutin with *ent*‐gallocatechin‐3‑*O*‑gallate and epigallocatechin‐3‑*O*‑gallate in aqueous solution resulted in weakening the color of the solution, being opposite to copigmentation (Figure 10E) [180].

The self‐association of small molecular phytochemicals to dimers and oligomers was also found in other phytochemicals. Doxorubicin (DOX) is a representative quinone compound characterized by a hydrophilic amino sugar ring and a hydrophobic planar anthraquinone group. Containing ionizable amine groups, DOX molecules can form dimers or oligomers, which makes it ideal building blocks of nanostructures [181]. Folic acid (vitamin B9), used as a dietary supplement, is composed of pterin, *p*‐aminobenzoic acid, and glutamic acid [182]. Folic acid can treat disease by targeting folate receptors overexpressed on the surface of tumor cells rather than on the surface of normal cells [183]. At a low concentration of 0.1%, folic acid ions could self‐assemble in aqueous media, which was realized via aromatic ring‐driven stacking [184]. Moreover, the hydrophilic group of folic acid is crucial for the formation of chiral columns in solvation.

### 4.2 Self‐assembly of Natural Small Molecular Phytochemical to Nanostructures

Inspired by the decoction process in traditional Chinese medicine, where medicinal efficacy is hypothesized to arise from the dissolution and spontaneous assembly of small‐molecule phytochemicals into nanoscale aggregates [185], increasing attention has been paid to the direct self‐assembly behavior of natural phytochemicals. A growing body of evidence suggests that these molecules can spontaneously organize into well‐defined nanostructures without the aid of exogenous carriers, thereby enhancing their biological activities.

Among various phytochemicals, alkaloids—characterized by the presence of basic nitrogen atoms—are one of the most extensively studied classes due to their strong intermolecular interaction capabilities [186, 187]. These compounds have been shown to interact with other phytochemicals to form diverse nanostructures. In particular, berberine (BBR), the principal antibacterial component of *Coptis chinensis* Franch, has attracted considerable interest because of its propensity to self‐assemble. This behavior is mainly attributed to its polyaromatic ring system and quaternary ammonium structure, which facilitate π–π stacking and electrostatic interactions [188]. BBR and baicalin are the active components of Huanglian Jiedu Decoction (consisting traditional herb pair *Coptidis rhizoma* and *Scutellariae radix*) [189]. The physical mixture of BBR and baicalin can self‐assemble into the form of nanofibers, which can transform to nanospheres by the co‐decocting (Figure 10A) [190]. The morphological changes from nanofibers to nanospheres can enhance antibacterial activity and the effect of biofilm clearance [191]. Similar phenomena have been observed in another classic herbal pair, *Rhei radix et rhizoma* and *Coptidis rhizoma* [185]. During co‐decoction of the herbal, the main components Rhein (Rhe) and coptisine could self‐assemble into nanofibers, while emodin and coptisine could self‐assemble into nanoparticles, both of which were induced by electrostatic attraction, hydrogen bonding, and π–π stacking [185].

Beyond herbal pair systems, binary self‐assembled nanoparticles formed by BBR and cinnamic acid (CA) exhibit markedly enhanced inhibition of multidrug resistance compared with free BBR (Figure 10B), underscoring the critical role of nanostructure formation in improving antibacterial efficacy [192]. Likewise, BBR and hesperetin can directly co‐assemble into carrier‐free binary nanoparticles through noncovalent interactions, including electrostatic forces, π–π stacking, and hydrogen bonding [193]. These nanoparticles exhibit significantly better alleviating effects on ulcerative colitis and inhibitory effects on inflammation than the individual phytochemicals [193].

Rhe, an anthraquinone derivative isolated from *Rheum*, possesses a hydrophobic planar anthraquinone unit and multiple hydroxyl and carboxyl groups, making it particularly amenable to supramolecular assembly. Rhe has been shown to co‐assemble with BBR into nanoparticles, in which Rhe molecules formed a layered framework stabilized by hydrogen bonding, while BBR molecules were embedded within this framework via π–π stacking and electrostatic interactions [194]. These Rhe‐BBR nanoparticles showed improved antimicrobial activity against *Staphylococcus aureus* compared with free BBR, primarily due to the strong inhibitory effects on *S. aureus* biofilm formation [194].

**FIGURE 11:**
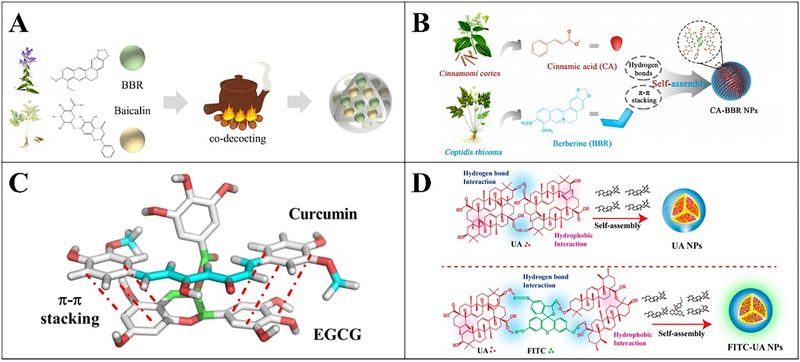
Self‐assembly of natural small molecular phytochemical to nanostructures. A) Schematic diagram of berberine (BBR) and baicalin forming nanospheres through co‐decoction. B) Berberine (BBR) and cinnamic acid self‐assemble to form nanoparticles; Reproduced with permission [192]. Copyright 2020, American Chemical Society. C) Simulation image of curcumin and EGCG interactions; Reproduced with permission [195]. Copyright 2024, Elsevier. D) Ursolic acid (UA) achieves molecular self‐assembly through electrostatic and hydrophobic interactions, forming nanoparticles; Reproduced with permission [198]. Copyright 2018, American Chemical Society.

Importantly, the self‐assembly of small‐molecule phytochemicals themselves—rather than their encapsulation within external delivery systems—has emerged as a central theme in contemporary nanomedicine research [19]. The self‐assembled systems not only preserve intrinsic pharmacological activity but also enable self‐transport and improved bioavailability. For instance, curcumin and EGCG can co‐assemble into nanoparticles with a size range of 100–150 nm (Figure 11C) [195]. The observed redshift in the UV–visible absorption spectrum (from 425 to 435 nm) indicated the formation of π–π stacking (Figure 11C). This nanoparticle system not only increased the dispersibility of curcumin in aqueous solutions but also exhibited enhanced stability against various environmental factors such as temperature, ionic strength, and pH [195].

In addition to polyphenols and alkaloids, certain steroids and terpenoids have been demonstrated to spontaneously self‐assemble to form nanostructures in solution. Cyclopentane‐poly(hydrophenanthrene), consisting of a rigid hydrophobic backbone, flexible alkyl side chains, and multiple chiral centers, is recognized as the nucleus of steroids molecules. Terpenoids and steroids are biosynthetically related natural products [196], while steroids generally exhibit higher conformational rigidity owing to their conserved fused‐ring framework, whereas terpenoids display greater structural diversity [197]. These structural features, combined with weak intermolecular forces, facilitate their self‐assembly into nanoaggregates. Ursolic acid (UA), a pentacyclic triterpenoid abundantly found in nature, can self‐assemble into nanoparticles with an average diameter of approximately 150 nm through electrostatic and hydrophobic interactions (Figure 11D) [198]. The self‐assembly behavior of triterpenoids is believed to be tremendously influenced by the rigid skeleton and the disposition of the substituent groups. Notably, UA nanoparticles showed the potential for immunotherapy with higher antiproliferative activity and increased the immunostimulatory activity compared with free UA, in both the in vitro and in vivo studies [198].

Collectively, these studies demonstrate that small‐molecule phytochemicals can spontaneously organize into discrete nanostructures—such as nanoparticles, nanofibers, and nanospheres—through noncovalent interactions including π–π stacking, hydrogen bonding, electrostatic attraction, and hydrophobic forces [199]. At this level, self‐assembly predominantly occurs at the molecular‐to‐nanoscale, leading to finite, dispersed nanostructures that function as carrier‐free nanomedicines with improved solubility, stability, and bioactivity [200]. Importantly, these assemblies remain colloidally dispersed and do not yet exhibit long‐range ordering or macroscopic continuity. In contrast, when such nanostructures further undergo hierarchical organization, phase separation, or network interconnection, they can give rise to macroscopic soft matter with emergent mechanical and rheological properties. This transition from discrete nanostructures to continuous soft materials constitutes a higher level of self‐assembly, which will be discussed in the following section.

### 4.3 Self‐assembly of Natural Small Molecular Phytochemical to Macroscopic Soft Matter

Distinct from the formation of discrete nanostructures discussed in Section 4.2, the emergence of macroscopic soft matter from small‐molecule phytochemicals requires long‐range ordering, hierarchical stacking, and network formation of primary assemblies. Such processes involve not only local intermolecular interactions but also collective behaviors, including phase separation [201], percolation [202], and interactions with surrounding solvent or polymeric environments [203]. These higher‐order assemblies give rise to soft matter systems—such as hydrogels, supramolecular films, and fibrous networks—that bridge the gap between molecular assemblies and bulk materials [203].

Soft matter refers to materials whose mechanical properties lie between those of simple liquids and crystalline solids, including colloids, hydrogels, polymers, and films. Unlike disordered aggregation, the intrinsic self‐assembly propensity of certain small‐molecule phytochemicals enables them to act as the building blocks for spontaneous formation of soft matter products with emergent mechanical strength, responsiveness, and biological functionality via ordered stacking.

**FIGURE 12:**
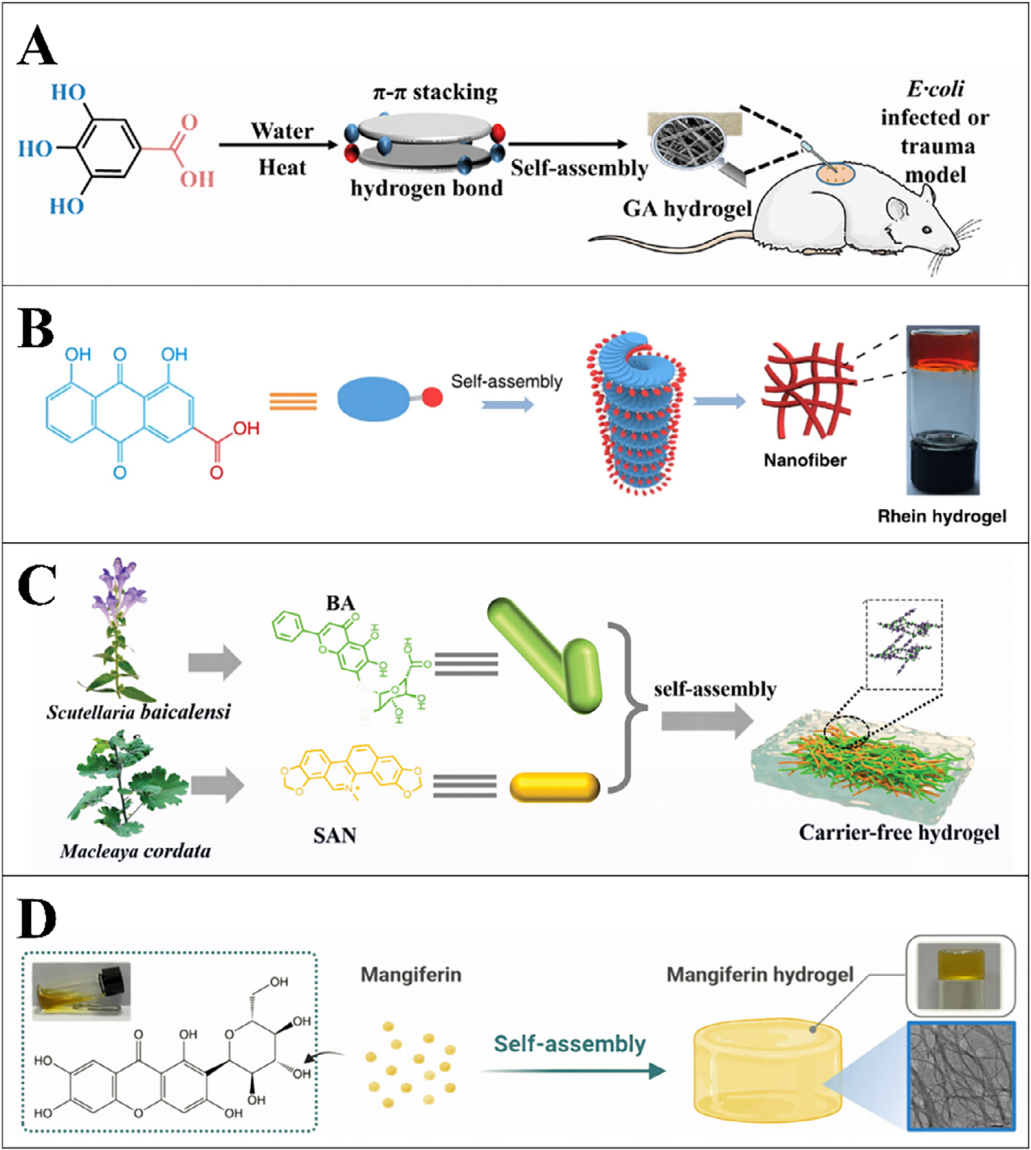
Self‐assembly of natural small molecular phytochemical to macroscopic soft matter. A) Gallic acid can uniquely form supramolecular hydrogels through π–π stacking and hydrogen bonds; Reproduced with permission [204]. Copyright 2022, Wiley. B) Self‐assembled of Rhein by noncovalent interaction to form nanofibers with left‐handed, and further crosslinked to form 3D network structure; Reproduced with permission [210]. Copyright 2019, Springer Nature. C) Self‐assembly illustration of carrier‐free baicalin‐sanguinarine (BA‐SAN) hydrogel; Reproduced with permission [211]. Copyright 2023, Wiley. D) Schematic illustration of the fabrication for Mangiferin hydrogel. Reproduced with permission [213]. Copyright 2024, American Chemical Society.

Gallic acid represented an example of structure‐specific soft matter formation. Gallic acid uniquely formed supramolecular hydrogels through π–π stacking and hydrogen bonds, exhibiting rod‐like structures (10–100 µm) with antibacterial properties and wound‐healing efficacy (Figure 12A) [204]. Notably, none of structurally related analogues formed hydrogels under the same condition of GA, demonstrating that molecular groups and structures played an important role in the formation of GA hydrogel [204].

Triterpenoids constitute another important class of phytochemicals capable of macroscopic self‐assembly [205]. Betulinic acid, a triterpenoid isolated from *Ziziphus jujuba*, forms strong gels in 19 organic solvents and alcohol‐water mixtures due to its pentacyclic rigid skeleton (1.31 nm) combined with terminal hydroxyl and carboxyl groups, which promote directional intermolecular interactions and nanofibril formation [206]. Similarly, glycyrrhizic acid self‐assembles into right‐handed twisted nanofibrils through ordered arrangement of its hydrophobic triterpenoid aglycon and hydrophilic diglucuronic acid moieties, ultimately generating stable supramolecular networks [207]. Notably, co‐assembly with phenolic compounds such as rosmarinic acid [208] or encapsulation into microspheres [209] has been shown to modulate hydrogel strength and stability, thereby enabling sustained release properties and enhanced functional performance.

Rhein further exemplifies pH‐regulated hierarchical assembly. Within the pH range of 6.8–8.0, Rhe molecules polymerize into nanofibrils via π–π stacking and hydrophilic interactions, while electrostatic repulsion between deprotonated carboxyl groups prevents uncontrolled aggregation. ultimately forming hydrogels with sustained drug release and enhanced anti‐neuroinflammatory activity (Figure 12B) [210]. This process highlights how a delicate balance between attractive and repulsive forces governs macroscopic material formation.

Beyond single‐component systems, multicomponent phytochemical assemblies can also yield functional soft matter. An example is the carrier‐free baicalin–sanguinarine binary hydrogel self‐assembles, which forms through electrostatic interactions, π–π stacking, and hydrogen bonding, exhibiting synergistic antibacterial and anti‐inflammatory activities along with favorable biocompatibility (Figure 12C) [211]. Similarly, thymol‐glycyrrhizin micelles undergo a pH‐triggered transition to hydrogels at pH∼4.9, showing antimicrobial potential [212].

Some polyphenols can directly form hydrogels without chemical modification. Mangiferin directly forms biocompatible hydrogels through π–π interactions and hydrogen bonds without structural modifications (Figure 12D) [213]. Furthermore, catechins exhibit distinct caffeine‐binding behaviors: EGCG and ECG form complexes with caffeine at the molecular ratios of 2:2 and 2:4, respectively, through multisite π–π stacking (A/B/B′ rings) and hydrogen bonds, leading to precipitation, whereas EC forms a complex (A‐ring only) with caffein at molecular ratio of 1:1 without precipitation due to lower hydrophobicity [214, 215, 216]. These differences highlight how aromatic ring interactions govern self‐assembly outcomes. These studies collectively demonstrate that small‐molecular phytochemicals can achieve ordered self‐assembly through intermolecular interactions, forming functional nanofibers and hydrogels, where structural specificity directly governs their assembly behavior and application efficacy.

Taken together, these studies demonstrate that unlike the discrete nanostructures described in Section 4.2, small‐molecule phytochemicals are capable of undergoing hierarchical self‐assembly beyond the nanoscale, forming continuous nanofibrillar networks and macroscopic soft matter.

## 5 Self‐assembly of Phytochemicals to Well‐Defined Soft Matter from Occasional to Guaranteed via Induced by Amyloid Template

**FIGURE 13:**
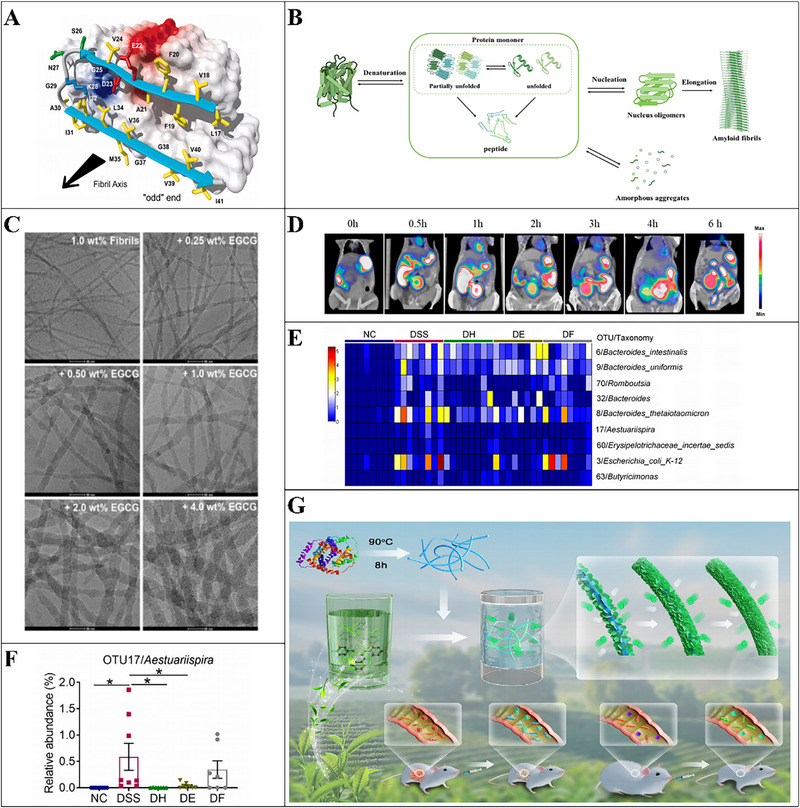
Self‐assembly of phytochemicals by amyloid template. A) The β‐sheets are indicated by cyan arrows in the 3D structure of a ^35Mox^ Alzheimer's amyloid‐β (1–42) fibril; Reproduced with permission [217]. Copyright 2005, National Academy of Sciences. B) Hydrolysis of proteins to form protein amyloid‐like fibrils; Reproduced with permission [218]. Copyright 2023, Elsevier; C) Cryo‐EM images of the amyloid fibril‐EGCG samples with different EGCG levels and the fibril concentration of 1.0 wt% at pH 3 [236]. Copyright 2020, American Chemical Society; D) Micro PET‐CT images of the amyloid–polyphenol hybrid nanofilament hydrogels loaded with ^18^F‐FDG (EGCG content of 1.5 wt% and lysozyme content of 1.0 wt%) in the gastrointestinal tract of a mouse at different time points after oral administration: 0, 0.5, 1, 2, 3, 4, and 6 h; Reproduced with permission [23]. Copyright 2020, American Chemical Society. E) The key OTUs contributing to separation of the gut microbial communities of different mice groups treated by different solution and amyloid–EGCG hydrogel shown in heat maps. The relative abundances of the OTUs in each group are shown by the spot colors, which were normalized and log‐transformed. The complete linkage method was used to cluster OTUs. On the right, each OTU's taxonomic level is shown. (NC: normal control group, DSS: induction of colitis by 2 wt% DSS followed by oral treatment with water, DH: the amyloid–EGCG hydrogels group, DE: EGCG solution group, DF: amyloid fibrils solution group); Reproduced with permission [23]. Copyright 2020, American Chemical Society. F) Relative abundances of *Aestuariispira* (OTU17); Reproduced with permission [23]. Copyright 2020, American Chemical Society. G) Polyphenols originating from teas can deposit on the surface of protein amyloid fibril, which effectively improves intestinal microbiota dysbiosis in mice with colitis and high‐fat models of the disease, thus alleviating related diseases.

The development of functional soft materials through the self‐assembly of small molecular phytochemicals represents a promising yet challenging frontier in biomaterial science. Phytochemicals, such as polyphenols, possess intrinsic bioactivities but face limitations in practical applications due to their poor stability, low solubility, and tendency toward disordered aggregation. Conventional self‐assembly strategies relying on weak intermolecular interactions such as hydrogen bonds, π–π stacking, and hydrophobic forces, often result in unpredictable structural outcomes and low yields, exacerbated by environmental sensitivities to solvents, temperature, and pH. Furthermore, the use of toxic solvents during fabrication and potential adverse biological effects of phytochemicals complicate their translational potential. To address these challenges, recent studies have explored food protein amyloid fibrils—a proteinaceous nanostructure with ordered β‐sheet configurations (Figure 13A) [217]—as universal templates to guide the hierarchical assembly of phytochemicals into macroscopic functional materials.

Protein is among the self‐assemblers ubiquitous in nature and any protein under certain suitable conditions is suggested to form amyloid or amyloid‐like fibrils [218, 219], including *in vitro de novo* synthesis via hydrolysis and re‐assembly of food‐grade proteins (Figure 13B)—a process induced by factors such as pH, metal ions, organic reagents, and temperature [220, 221]. Amyloid fibrils, initially linked to neurodegenerative diseases [222], are now recognized for their structural versatility and biocompatibility [223, 224]. The safety of amyloid fibrils have been investigated and confirmed [225]. A comprehensive safety assessment of amyloid fibrils derived from food proteins, represented by β‐lactoglobulin and lysozyme, revealed that these amyloid fibrils can be effectively degraded into randomly coiled oligopeptides and amino acids [225]. In certain cases, such as lysozyme, the degree of digestion even surpasses that of native protein monomers. The in vivo results proved that no signs of cytotoxicity of digested fibrils were observed in *C. elegans* or mouse models, nor cross‐seeding events from digested fibrils could be detected in the two animal models. The β‐lg and lysozyme fibrils could be digested into the same oligopeptides as their monomers. Any unabsorbed digested oligopeptides of fibrils do not enter the bloodstream and instead were further metabolized and excreted out of the body together with feces in mice models. No cross‐seeding of disease‐related protein aggregates (polyQ35) was observed with digested fibrils and no amyloid plaque formation was detected in the brain or other major organs. Based on multiple in vitro and in vivo evidences, this study concludes that digested food protein amyloid fibrils are at least as safe as digested native protein monomers. The demonstrated safety profile of food protein amyloid fibrils positions these biomaterials as viable dietary ingredients, unveiling their transformative potential for innovative applications across nutritional science and functional food development [225]. Positive roles played by amyloid motifs in many different biological contexts were found recently, not only structural scaffolds in various cellular tasks [226, 227] and storage and transport of peptide hormones [228], but also inducing immune regulatory pathway [229], attenuating inflammation [230], and antibacterial activities [231]. In addition, protein amyloid fibrils can be formed through the self‐assembly of repurposed proteins from food waste, thereby achieving sustainable development goals on the UN 2030 agenda and offering new possibilities for sustainable technology portfolios in protein utilization [232].

These fibrils exhibit a cross‐β architecture stabilized by hydrophobic interactions, hydrogen bonds, and van der Waals forces, endowing them with robust templating capabilities that enable sequence‐independent self‐assembly and hierarchical organization [233]. This generic structural motif confers exceptional thermodynamic stability and mechanical strength, often exceeding that of conventional biopolymer scaffolds. In addition, the high‐aspect‐ratio morphology and periodic β‐sheet arrangement provide regularly spaced binding sites, facilitating nanoscale templating and surface functionalization. Importantly, these properties allow amyloid fibrils to function as adaptable scaffolds, while retaining tunability through sequence design and environmental control. For instance, β‐lactoglobulin‐derived amyloid fibrils have been employed to stabilize iron nanoparticles, forming colloids with enhanced bioavailability equivalent to commercial ferrous sulfate meanwhile preventing oxidative aggregation [234]. Similarly, fibril‐guided nucleation of gold and silver nanoparticles enabled the fabrication of ultralight aerogels (20 kt purity) with metal‐like stiffness through controlled biosilicification [235]. Despite these advances, applications remained confined to inorganic systems until the recent discovery of amyloid fibrils as mediators for phytochemical assembly.

Recently, it was demonstrated that amyloid fibrils successfully bridged the length scales between small molecular phytochemicals of less than 1 nm and macroscopic hydrogels based on amyloid‐polyphenol templates [236, 237]. Polyphenols were demonstrated to attach and stack on the surface of amyloid fibrils originating from lysozyme to form the protein‐polyphenol “core–shell” hybrid super‐molecule filaments with enlarged diameter as the content of polyphenol increased (Figure 13C) [236, 237]. The protein‐polyphenol “core–shell” hybrid filaments further assembled to form the hydrogels spontaneously, in which amyloid fibrils and polyphenols acted both as the building blocks and functional materials with biological activities. Here, distinct from the previous researches, the formation of the hydrogels is ascribed to the molecular self‐assemble property of polyphenols rather than the crosslinking function. Furthermore, and more importantly, the properties and functionalities of single polyphenols were reinforced by these hybrids, in particular by allowing the use of high concentrations with chemical stability, which would not be possible with polyphenols alone. The highest content of polyphenols in the hydrogels reached as high as 4.0 wt% [236], which, to the best of our known, is the highest polyphenol loading level among delivery systems ever reported in the literature, and shows potentials for real‐world applications in the biomedical and functional food industries. The turbidity, flocculation, and precipitate inclined to be caused by the polyphenol‐induced unordered aggregation of protein monomers was overcame. The stability of polyphenols during storage and thermal treatment was very significantly improved after encapsulation in the hybrid hydrogels.

In fact, it was surprising to obtain this result as polyphenols had been well known to inhibit the formation of amyloid fibrils from amyloid peptides or cut the formed mature amyloid fibrils [238, 239]. Moreover, the findings were convinced to be a general scientific phenomenon as 14 different polyphenols were mixed with lysozyme amyloid fibrils to form the hybrid hydrogels and the amyloid fibrils fabricated from lysostaphin M23 endopeptidase were induced by polyphenol to form hydrogels [23]. The gallate ester moiety in the polyphenol molecules was revealed to play essential roles in the interaction with protein amyloid fibrils: the affinity between polyphenols and the amyloid fibrils was enhanced with the increased number of the gallate ester moiety in the molecules [23, 237]. It implied that the intermolecular π–π stacking could be the major driven force in the interaction between polyphenols and amyloid fibrils. The polyphenols with higher hydrophobicity showed stronger affinity to the amyloid fibrils [23].

The self‐assembly of phytochemicals onto the protein/peptide fibrils hinges on the molecular complementarity between their structure and the assembly driving forces and surface chemistry of the protein/peptide fibrils. Different classes of phytochemicals bind to amyloid fibrils through distinct physicochemical mechanisms, thereby achieving “diverse assembly”. This primarily relies on the interaction forces such as π–π stacking, hydrophobic forces, hydrogen bonds, and electrostatic interactions [240]. Currently, computational studies, particularly molecular dynamics simulations, have demonstrated that polyphenolic phytochemicals such as EGCG [241] and curcumin [242, 243] can stably associate with amyloid protofibrils through a combination of hydrophobic interactions, π–π stacking, and hydrogen bonding, preferentially targeting hydrophobic surface grooves or fibril growth ends. Amentoflavone (AMF), a natural biflavonoid compound, was found preferentially bind to the ^16^KLVFFAEDV [24] segment in amyloid‐β peptide by molecular dynamics (REMD) and molecular mechanics/Poisson–Boltzmann surface area (MM/PBSA) method [244]. The gallic acid ester group has been demonstrated to play an important role in the self‐assembly of flavonoids induced by the protein fibrils [23, 245]. Future studies may integrate computational modeling and other techniques to predict the binding potential of diverse plant compound categories with amyloid fibrils by forecasting binding affinities and optimizing structural modifications.

The unique stability of the polyphenol‐amyloid fibrils hybrid hydrogels against high thermal treatment is impressive [23, 245], indicating their potentials to resist the thermal sterilization processing in real‐world applications [246, 247]. The hybrid hydrogels are shear thinning and reversible, allowing them to transport through the GI tract and maintain in gut for several hours before excreting as feces, during which the hydrogels flowed maintaining the bulk gel property (Figure 13D). Oral delivery of drugs or pharmaceutics through the GI tract to gut is a big challenge, and the payloads are difficult to arrive at gut (cecum, colon, rectum) and further stay within for a long time. Only when the drugs or pharmaceutics are plentifully delivered across the GI tract to the gut can they exert the effect on modulating the gut microbiota. This adaptable hydrogel network formed through the assembly of polyphenols and amyloid fibrils are supposed to be desirable and promising as a platform for treatment of diseases highly associated with gut, attributing to their water‐rich nature, changeable viscoelasticity, surface charge, and interaction with mucus layer on epithelial surface. Study demonstrated that oral administration of the polyphenol‐amyloid fibril hydrogels alleviated colitis in the mice model induced by DSS with the recovered clinical symptoms (Figure 13E) [236]. It is important to point out that the alleviation effect of the polyphenol‐amyloid fibril hydrogels on mouse colitis is dependent on the polyphenol contents embedded in the hybrid hydrogels. The hydrogels composed with 0.5 and 1.0 wt% polyphenols stacking on the surface of amyloid fibrils did not show obvious alleviation effects on the DSS‐induced colitis [236]. Similarly, flavonoid‐amyloid fibril hybrid hydrogel inhibits high‐fat diet‐induced obesity in mice by significantly increasing flavonoid loading in the hydrogel due to the adhesion and deposition of flavonoid molecules on the surface of amyloid fibrils [237]. These results indicated the high enough loading contents of phytochemicals for the soft matter is essential to exert the corresponding biological activities, which could only be realized definitely via molecule self‐assembly of the phytochemicals templated by amyloid fibrils.

Oral administration with the polyphenol‐amyloid fibril hydrogels also enriched the α diversity of the gut microbiota and recovered the diverging of gut microbiota caused by DSS treatment, thus modifying the microbiota structure significantly [236]. oral treatment of the colitis mice with the amyloid–EGCG hydrogels inhibited the enrichment of these phylotypes in the gut microbiota (Figure 13E) [236]. In particular, the enrichment of *Aestuariispira* (OTU17) was significantly inhibited by oral treatment with the amyloid–EGCG hydrogels (Figure 13F). On the other hand, the level of gut microbiota metabolites SCFAs was elevated after oral administration of the hydrogels [236]. In the in vitro antibacterial assay, incubation with the polyphenol‐binding amyloid fibrils induced bacterial agglomeration and immobilization on both Gram‐positive and Gram‐negative bacteria [23]. The antibacterial mechanism of the lysozyme amyloid fibril hydrogels was initiated by membrane disintegration as demonstrated in *Escherichia coli* K12 [23]. For human colonic epithelial cells, incubation with these hybrid supramolecules was demonstrated to show lack of cytotoxicity [23]. In addition, by transplanting the gut microbiota educated by flavonoid‐amyloid fibril hydrogel to germ‐free mice, it was found to form a unique microbiota structure against high‐fat diet (HFD)‐induced structural differentiation of the microbiota [237]. This suggests that hybrid hydrogels can inhibit the molecular link between gut microbes and host intestinal lipid absorption, providing a general concept for designing precise clinical approaches to obesity prevention by targeting the host‐microbiota crosstalk (Figure 13G). Recently, a study showed that the geometric mean absorption of iron in iron‐deficient women, a key target group for iron fortification, was 46.2% when the hybrids carrying ultrasmall iron nanoparticles by oat protein nanofibrils (OatNF‐SA‐Fe) were administered with water, achieving very high values of bioavailability corresponding to 176% of FeSO_4_ [248]. This is the first study to propose and measure iron absorption from protein–iron nanoparticle hybrid systems in humans. These studies established a generalizable framework for designing biofunctional soft materials through the integration of amyloid fibrils’ molecular recognition capabilities with phytochemicals, providing an effective research foundation for their applications in targeted therapy, functional foods, and precision nutritional interventions.

## 6 Conclusion and Prospective

Unarguably, the nutritional functions of edible bioactive phytochemicals have been proven to alleviate various chronic diseases through the action of intestinal microorganisms. This article reviews the various encapsulation technologies currently available for phytochemicals and their application effects. How to increase the concentration of phytochemicals in food matrices without readily inducing disordered aggregation of biomolecules into precipitates remains a challenge to be addressed. Through self‐assembly templated by amyloid fibrils, phytochemicals demonstrate transformative potential in precision nutrition by modulating gut microbiota and alleviating chronic diseases via high‐capacity, ordered encapsulation that overcomes traditional delivery limitations. These systems leverage non‐covalent interactions to stabilize bioactive compounds, enhance gut‐targeted release, and restore microbial balance.

**FIGURE 14:**
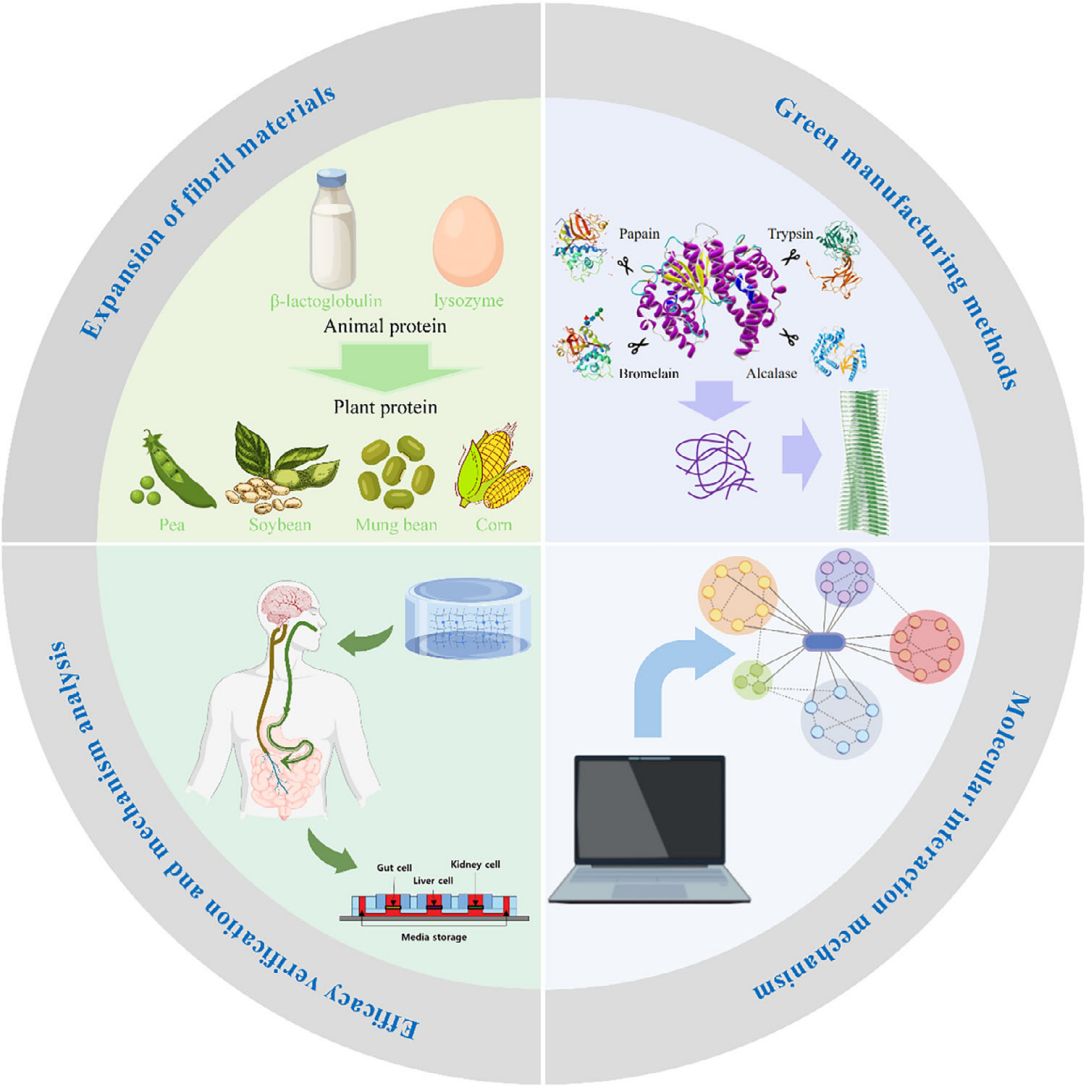
Possible future directions for amyloid fibrils in precision nutrition.

However, significant challenges remain, including the reliance on animal‐derived fibrils, such as lysozyme, and the fact that insufficient control of fibril‐phytochemical interactions renders the self‐assembly process largely stochastic. Future advancements require diversifying protein sources and developing green manufacturing methods for the large‐scale production of food‐grade amyloid fibrils such as enzymatic fibrillation, which is a critical next step for commercial translation. In addition, expanding this platform to efficiently load hydrophobic phytochemicals and understanding the templating mechanism for non‐polyphenol compounds is a key research frontier. Comprehensive toxicological studies, including long‐term feeding trials across multiple model systems, are required to firmly establish the safety of various food‐protein amyloid fibrils for human consumption. Meanwhile, with the continuous advancement and application potential of human organoid technology, validating preventive and alleviating effect in biomimetic human gut models will be essential to transform serendipitous assembly into scalable, precision‐targeted therapies for chronic disease management. From a future perspective, the integration of computational simulation and modeling techniques is expected to enable the rational assembly of small‐molecule phytochemicals on amyloid fibril templates. Such approaches may provide predictive control over intermolecular interactions and assembly pathways, thereby facilitating the design of phytochemical–amyloid hybrid systems with tailored structures and functions. Naturally, as a novel food with immense application potential, its regulation will also be a key focus in the future, thereby enabling its more refined implementation (Figure 14).

## Conflicts of Interest

The authors declare that they have no conflicts of interest.

## Data Availability

The authors have nothing to report.

## References

[1] P. J. Stover and J. C. King , “More Nutrition Precision, Better Decisions for the Health of Our Nation,” The Journal of Nutrition 150, no. 12 (2020): 3058–3060, 10.1093/jn/nxaa280. 33025003 PMC7919333

[2] D. D. Wang , Y. Li , A. Afshin , et al., “Global Improvement in Dietary Quality Could Lead to Substantial Reduction in Premature Death,” The Journal of Nutrition 149, no. 6 (2019): 1065–1074, 10.1093/jn/nxz010. 31049577 PMC6543201

[3] L. Abarca‐Gómez , Z. A. Abdeen , Z. A. Hamid , et al., “Worldwide Trends in Body‐Mass Index, Underweight, Overweight, and Obesity from 1975 to 2016: a Pooled Analysis of 2416 Population‐Based Measurement Studies in 128·9 Million Children, Adolescents, and Adults,” The Lancet 390, no. 10113 (2017): 2627–2642, 10.1016/S0140-6736(17)32129-3. PMC5735219 29029897

[4] D. G. Gadhave , V. V. Sugandhi , S. K. Jha , et al., “Neurodegenerative Disorders: Mechanisms of Degeneration and Therapeutic Approaches with Their Clinical Relevance,” Ageing Research Reviews 99 (2024): 102357, 10.1016/j.arr.2024.102357. 38830548

[5] C. Petersen , R. Bell , K. A. Klag , et al., “T Cell–mediated Regulation of the Microbiota Protects against Obesity,” Science 365, no. 6451 (2019): aat9351, 10.1126/science.aat9351. PMC7294966 31346040

[6] J. Lloyd‐Price , C. Arze , A. N. Ananthakrishnan , et al., “Multi‐Omics of the Gut Microbial Ecosystem in Inflammatory Bowel Diseases,” Nature 569, no. 7758 (2019): 655–662, 10.1038/s41586-019-1237-9. 31142855 PMC6650278

[7] B. Bayram , A. González‐Sarrías , G. Istas , et al., “Breakthroughs in the Health Effects of Plant Food Bioactives: a Perspective on Microbiomics, Nutri(Epi)Genomics, and Metabolomics,” Journal of Agricultural and Food Chemistry 66, no. 41 (2018): 10686–10692, 10.1021/acs.jafc.8b03385. 30208704

[8] L. Brennan and B. De Roos , “Nutrigenomics: Lessons Learned and Future Perspectives,” The American Journal of Clinical Nutrition 113, no. 3 (2021): 503–516, 10.1093/ajcn/nqaa366. 33515029

[9] D. D. Wang and F. B. Hu , “Precision Nutrition for Prevention and Management of Type 2 Diabetes,” The Lancet Diabetes & Endocrinology 6, no. 5 (2018): 416–426, 10.1016/S2213-8587(18)30037-8. 29433995

[10] S. Andraos , M. Wake , R. Saffery , D. Burgner , M. Kussmann , and J. O'Sullivan , “Perspective: Advancing Understanding of Population Nutrient‐Health Relations via Metabolomics and Precision Phenotypes,” Advances in Nutrition 10, no. 6 (2019): 944–952, 10.1093/advances/nmz045. 31098626 PMC6855971

[11] R. E. Ley , P. J. Turnbaugh , S. Klein , and J. I. Gordon , “Human Gut Microbes Associated with Obesity,” Nature 444, no. 7122 (2006): 1022–1023, 10.1038/4441022a. 17183309

[12] B. Chassaing , O. Koren , J. K. Goodrich , et al., “Dietary Emulsifiers Impact the Mouse Gut Microbiota Promoting Colitis and Metabolic Syndrome,” Nature 519, no. 7541 (2015): 92–96, 10.1038/nature14232. 25731162 PMC4910713

[13] Z. Goktas , Y. Zu , M. Abbasi , et al., “Recent Advances in Nanoencapsulation of Phytochemicals to Combat Obesity and Its Comorbidities,” Journal of Agricultural and Food Chemistry 68, no. 31 (2020): 8119–8131, 10.1021/acs.jafc.0c00131. 32633507 PMC8507418

[14] S. Caballero , Y. O. Li , D. J. McClements , and G. Davidov‐Pardo , “Encapsulation and Delivery of Bioactive Citrus Pomace Polyphenols: a Review,” Critical Reviews in Food Science and Nutrition 62, no. 29 (2022): 8028–8044, 10.1080/10408398.2021.1922873. 33983085

[15] M. Peanparkdee and S. Iwamoto , “Encapsulation for Improving In Vitro Gastrointestinal Digestion of Plant Polyphenols and Their Applications in Food Products,” Food Reviews International 38, no. 4 (2022): 335–353, 10.1080/87559129.2020.1733595.

[16] K. Liang , J. E. Chung , S. J. Gao , N. Yongvongsoontorn , and M. Kurisawa , “Highly Augmented Drug Loading and Stability of Micellar Nanocomplexes Composed of Doxorubicin and Poly(Ethylene Glycol)‐Green Tea Catechin Conjugate for Cancer Therapy,” Advanced Materials 30, no. 14 (2018): 1706963, 10.1002/adma.201706963. 29473233

[17] C. Yan , X. Zhu , B. Chen , H. Guan , K. Gu , and H. Liu , “Protein/Peptide‐Polyphenol Interactions: Molecular Mechanisms, Functional Synergy, and Emerging Applications,” Trends in Food Science & Technology 163 (2025): 105194, 10.1016/j.tifs.2025.105194.

[18] Z. Yi , X. Chen , G. Chen , et al., “General Nanomedicine Platform by Solvent‐Mediated Disassembly/Reassembly of Scalable Natural Polyphenol Colloidal Spheres,” ACS Applied Materials & Interfaces 12, no. 34 (2020): 37914–37928, 10.1021/acsami.0c11650. 32805962

[19] J. Zhu , Z. Zhang , R. Wang , et al., “Review of Natural Phytochemical‐Based Self‐Assembled Nanostructures for Applications in Medicine,” ACS Applied Nano Materials 5, no. 3 (2022): 3146–3169, 10.1021/acsanm.2c00056.

[20] N. Stephanopoulos , “Hybrid Nanostructures from the Self‐Assembly of Proteins and DNA,” Chem 6, no. 2 (2020): 364–405, 10.1016/j.chempr.2020.01.012.

[21] M. Diener , J. Adamcik , A. Sánchez‐Ferrer , F. Jaedig , L. Schefer , and R. Mezzenga , “Primary, Secondary, Tertiary and Quaternary Structure Levels in Linear Polysaccharides: From Random Coil, to Single Helix to Supramolecular Assembly,” Biomacromolecules 20, no. 4 (2019): 1731–1739, 10.1021/acs.biomac.9b00087. 30816699

[22] N. Singh , K. Patel , A. Navalkar , et al., “Amyloid Fibril‐Based Thixotropic Hydrogels for Modeling of Tumor Spheroids In Vitro,” Biomaterials 295 (2023): 122032, 10.1016/j.biomaterials.2023.122032. 36791521

[23] B. Hu , Y. Shen , J. Adamcik , et al., “Polyphenol‐Binding Amyloid Fibrils Self‐Assemble into Reversible Hydrogels with Antibacterial Activity,” ACS nano 12, no. 4 (2018): 3385–3396, 10.1021/acsnano.7b08969. 29553709

[24] L. Dong , Y. Zhang , Y. Li , et al., “The Binding Mechanism of Oat Phenolic Acid to Whey Protein and Its Inhibition Mechanism against AGEs as Revealed Using Spectroscopy, Chromatography and Molecular Docking,” Food & Function 14, no. 22 (2023): 10221–10231, 10.1039/D3FO02474A. 37916290

[25] B. R. Da Silva , L. Brennan , M. A. Horst , D. S. Wishart , and C. M. Prado , “Advancing Precision Nutrition: Bridging Mechanistic Insight and Clinical Implementation,” Nature Reviews Endocrinology 21, no. 9 (2025): 515–517, 10.1038/s41574-025-01141-9. 40523984

[26] M. D. Sakhawot Hossain , M. D. Abdul Wazed , S. Asha , M. D. Ruhul Amin , and I. Md Shimul , “Dietary Phytochemicals in Health and Disease: Mechanisms, Clinical Evidence, and Applications—A Comprehensive Review,” Food Science & Nutrition 13, no. 3 (2025): 70101, 10.1002/fsn3.70101. PMC11922683 40115248

[27] J. Wu , K. Wang , X. Wang , Y. Pang , and C. Jiang , “The Role of the Gut Microbiome and Its Metabolites in Metabolic Diseases,” Protein & Cell 12, no. 5 (2021): 360–373, 10.1007/s13238-020-00814-7. 33346905 PMC8106557

[28] D. Liu , J. Huang , Y. Luo , et al., “Fuzhuan Brick Tea Attenuates High‐Fat Diet‐Induced Obesity and Associated Metabolic Disorders by Shaping Gut Microbiota,” Journal of Agricultural and Food Chemistry 67, no. 49 (2019): 13589–13604, 10.1021/acs.jafc.9b05833. 31735025

[29] E. Molinar‐Toribio , E. Fuguet , S. Ramos‐Romero , et al., “A High‐Fat High‐Sucrose Diet Affects the Long‐Term Metabolic Fate of Grape Proanthocyanidins in Rats,” European Journal of Nutrition 57, no. 1 (2018): 339–349, 10.1007/s00394-016-1323-9. 27730364

[30] L. Zhang , R. N. Carmody , H. M. Kalariya , et al., “Grape Proanthocyanidin‐Induced Intestinal Bloom of Akkermansia Muciniphila Is Dependent on Its Baseline Abundance and Precedes Activation of Host Genes Related to Metabolic Health,” The Journal of Nutritional Biochemistry 56 (2018): 142–151, 10.1016/j.jnutbio.2018.02.009. 29571008 PMC5971143

[31] D. P. Singh , S. Singh , V. Bijalwan , et al., “Co‐Supplementation of Isomalto‐Oligosaccharides Potentiates Metabolic Health Benefits of Polyphenol‐Rich Cranberry Extract in High Fat Diet‐Fed Mice via Enhanced Gut Butyrate Production,” European Journal of Nutrition 57, no. 8 (2018): 2897–2911, 10.1007/s00394-017-1561-5. 29127476

[32] M.‐C. Rodríguez‐Daza , L. Daoust , L. Boutkrabt , et al., “Wild Blueberry Proanthocyanidins Shape Distinct Gut Microbiota Profile and Influence Glucose Homeostasis and Intestinal Phenotypes in High‐Fat High‐Sucrose Fed Mice,” Scientific Reports 10, no. 1 (2020): 2217, 10.1038/s41598-020-58863-1. 32041991 PMC7010699

[33] Y. Zhu , J.‐Y. Zhang , Y.‐L. Wei , et al., “The Polyphenol‐Rich Extract from Chokeberry (Aronia Melanocarpa L.) Modulates Gut Microbiota and Improves Lipid Metabolism in Diet‐Induced Obese Rats,” Nutrition & Metabolism 17, no. 1 (2020): 54, 10.1186/s12986-020-00473-9. 32655675 PMC7339576

[34] D. Li , Y. Cui , X. Wang , F. Liu , and X. Li , “Apple Polyphenol Extract Improves High‐Fat Diet‐Induced Hepatic Steatosis by Regulating Bile Acid Synthesis and Gut Microbiota in C57BL/6 Male Mice,” Journal of Agricultural and Food Chemistry 69, no. 24 (2021): 6829–6841, 10.1021/acs.jafc.1c02532. 34124904

[35] X. Jiao , Y. Wang , Y. Lin , et al., “Blueberry Polyphenols Extract as a Potential Prebiotic with Anti‐Obesity Effects on C57BL/6 J Mice by Modulating the Gut Microbiota,” The Journal of Nutritional Biochemistry 64 (2019): 88–100, 10.1016/j.jnutbio.2018.07.008. 30471564

[36] W. Liu , S. Zhao , J. Wang , et al., “Grape Seed Proanthocyanidin Extract Ameliorates Inflammation and Adiposity by Modulating Gut Microbiota in High‐Fat Diet Mice,” Molecular Nutrition & Food Research 61, no. 9 (2017), 10.1002/mnfr.201601082. 28500724

[37] F. F. Anhê , D. Roy , G. Pilon , et al., “A Polyphenol‐Rich Cranberry Extract Protects from Diet‐Induced Obesity, Insulin Resistance and Intestinal Inflammation in Association with Increased Akkermansia Spp. Population in the Gut Microbiota of Mice,” Gut microbiota 64, no. 6 (2015): 872–883, 10.1136/gutjnl-2014-307142. 25080446

[38] A. Morissette , C. Kropp , J.‐P. Songpadith , et al., “Blueberry Proanthocyanidins and Anthocyanins Improve Metabolic Health through a Gut Microbiota‐Dependent Mechanism in Diet‐Induced Obese Mice,” American Journal of Physiology‐Endocrinology and Metabolism 318, no. 6 (2020): E965–E980, 10.1152/ajpendo.00560.2019. 32228321

[39] A. J. Forgie , Y. Gao , T. Ju , et al., “Pea Polyphenolics and Hydrolysis Processing Alter Microbial Community Structure and Early Pathogen Colonization in Mice,” The Journal of Nutritional Biochemistry 67 (2019): 101–110, 10.1016/j.jnutbio.2019.01.012. 30877891

[40] H.‐H. Zhang , J. Liu , Y.‐J. Lv , et al., “Changes in Intestinal Microbiota of Type 2 Diabetes in Mice in Response to Dietary Supplementation with Instant Tea or Matcha,” Canadian Journal of Diabetes 44, no. 1 (2020): 44–52, 10.1016/j.jcjd.2019.04.021. 31378691

[41] T. Chen , A. B. Liu , S. Sun , et al., “Green Tea Polyphenols Modify the Gut Microbiome in db/db Mice as Co‐Abundance Groups Correlating with the Blood Glucose Lowering Effect,” Molecular Nutrition & Food Research 63, no. 8 (2019): 1801064, 10.1002/mnfr.201801064. PMC6494111 30667580

[42] R. Lin , X. He , H. Chen , et al., “Oil Tea Improves Glucose and Lipid Levels and Alters Gut Microbiota in Type 2 Diabetic Mice,” Nutrition Research 57 (2018): 67–77, 10.1016/j.nutres.2018.05.004. 30122197

[43] J.‐M. Park , Y. Shin , S. H. Kim , M. Jin , and J. J. Choi , “Dietary Epigallocatechin‐3‐Gallate Alters the Gut Microbiota of Obese Diabetic Db/Db Mice: Lactobacillus Is a Putative Target,” Journal of Medicinal Food 23, no. 10 (2020): 1033–1042, 10.1089/jmf.2020.4700. 33054538

[44] Y. Yuan , Y. Zheng , J. Zhou , et al., “Polyphenol‐Rich Extracts from Brown Macroalgae Lessonia Trabeculate Attenuate Hyperglycemia and Modulate Gut Microbiota in High‐Fat Diet and Streptozotocin‐Induced Diabetic Rats,” Journal of Agricultural and Food Chemistry 67, no. 45 (2019): 12472–12480, 10.1021/acs.jafc.9b05118. 31642672

[45] R. Caruso , B. C. Lo , and G. Núñez , “Host–Microbiota Interactions in Inflammatory Bowel Disease,” Nature Reviews Immunology 20, no. 7 (2020): 411–426, 10.1038/s41577-019-0268-7. 32005980

[46] D. N. Frank , A. L. St Amand , R. A. Feldman , E. C. Boedeker , N. Harpaz , and N. R. Pace , “Molecular‐Phylogenetic Characterization of Microbial Community Imbalances in Human Inflammatory Bowel Diseases,” Proceedings of the National Academy of Sciences 104 (2007): 13780–13785, 10.1073/pnas.0706625104. PMC1959459 17699621

[47] J. Ni , G. D. Wu , L. Albenberg , and V. T. Tomov , “Gut Microbiota and IBD: Causation or Correlation?,” Nature Reviews Gastroenterology & Hepatology 14, no. 10 (2017): 573–584, 10.1038/nrgastro.2017.88. 28743984 PMC5880536

[48] F. Liu , X. Wang , D. Li , Y. Cui , and X. Li , “Apple Polyphenols Extract Alleviated Dextran Sulfate Sodium‐Induced Ulcerative Colitis in C57BL /6 Male Mice by Restoring Bile Acid Metabolism Disorder and Gut Microbiota Dysbiosis,” Phytotherapy Research 35, no. 3 (2021): 1468–1485, 10.1002/ptr.6910. 33215776

[49] H. Kim , N. Banerjee , M. A. Sirven , et al., “Pomegranate Polyphenolics Reduce Inflammation and Ulceration in Intestinal Colitis—Involvement of the miR‐145/p70S6K1/HIF1α Axis In Vivo and In Vitro,” The Journal of Nutritional Biochemistry 43 (2017): 107–115, 10.1016/j.jnutbio.2017.02.005. 28282584

[50] M. Larrosa , A. González‐Sarrías , M. J. Yáñez‐Gascón , et al., “Anti‐inflammatory Properties of a Pomegranate Extract and Its Metabolite Urolithin‐A in a Colitis Rat Model and the Effect of Colon Inflammation on Phenolic Metabolism,” The Journal of Nutritional Biochemistry 21, no. 8 (2010): 717–725, 10.1016/j.jnutbio.2009.04.012. 19616930

[51] K. Wang , X. Jin , Q. Li , et al., “Propolis from Different Geographic Origins Decreases Intestinal Inflammation and Bacteroides spp. Populations in a Model of DSS‐Induced Colitis,” Molecular Nutrition & Food Research 62, no. 17 (2018): 1800080, 10.1002/mnfr.201800080. 29889351

[52] X. Xu , H. Hua , L. Wang , et al., “Holly Polyphenols Alleviate Intestinal Inflammation and Alter Microbiota Composition in Lipopolysaccharide‐Challenged Pigs,” British Journal of Nutrition 123, no. 8 (2020): 881–891, 10.1017/S0007114520000082. 31928547

[53] H. Kim , V. P. Venancio , C. Fang , A. W. Dupont , S. T. Talcott , and S. U. Mertens‐Talcott , “Mango (*Mangifera indica* L.) Polyphenols Reduce IL‐8, GRO, and GM‐SCF Plasma Levels and Increase Lactobacillus Species in a Pilot Study in Patients with Inflammatory Bowel Disease,” Nutrition Research 75 (2020): 85–94, 10.1016/j.nutres.2020.01.002. 32109839

[54] P. Wan , Y. Peng , G. Chen , et al., “Dicaffeoylquinic Acids from *Ilex Kudingcha* Attenuate Dextran Sulfate Sodium‐Induced Colitis in C57BL/6 Mice in Association with the Modulation of Gut Microbiota,” Journal of Functional Foods 61 (2019): 103468, 10.1016/j.jff.2019.103468.

[55] K. Gil‐Cardoso , R. Comitato , I. Ginés , et al., “Protective Effect of Proanthocyanidins in a Rat Model of Mild Intestinal Inflammation and Impaired Intestinal Permeability Induced by LPS,” Molecular Nutrition & Food Research 63, no. 8 (2019): 1800720, 10.1002/mnfr.201800720. 30656830

[56] L. M. Bode , D. Bunzel , M. Huch , et al., “In Vivo and In Vitro Metabolism of Trans‐Resveratrol by Human Gut Microbiota,” The American Journal of Clinical Nutrition 97, no. 2 (2013): 295–309, 10.3945/ajcn.112.049379. 23283496

[57] G. Jamar , A. B. Santamarina , B. P. Casagrande , et al., “Prebiotic Potential of Juçara Berry on Changes in Gut Bacteria and Acetate of Individuals with Obesity,” European Journal of Nutrition 59, no. 8 (2020): 3767–3778, 10.1007/s00394-020-02208-1. 32108262

[58] S. Vendrame , S. Guglielmetti , P. Riso , S. Arioli , D. Klimis‐Zacas , and M. Porrini , “Six‐Week Consumption of a Wild Blueberry Powder Drink Increases Bifidobacteria in the Human Gut,” Journal of Agricultural and Food Chemistry 59, no. 24 (2011): 12815–12820, 10.1021/jf2028686. 22060186

[59] A. González‐Sarrías , M. Romo‐Vaquero , R. García‐Villalba , A. Cortés‐Martín , M. V. Selma , and J. C. Espín , “The Endotoxemia Marker Lipopolysaccharide‐Binding Protein Is Reduced in Overweight‐Obese Subjects Consuming Pomegranate Extract by Modulating the Gut Microbiota: a Randomized Clinical Trial,” Molecular Nutrition & Food Research 62, no. 11 (2018): 1800160, 10.1002/mnfr.201800160. 29665619

[60] M. Silva , C. Cueva , C. Alba , et al., “Gut Microbiome‐Modulating Properties of a Polyphenol‐Enriched Dietary Supplement Comprised of Hibiscus and Lemon Verbena Extracts. Monitoring of Phenolic Metabolites,” Journal of Functional Foods 91 (2022): 105016, 10.1016/j.jff.2022.105016.

[61] J. Park , Q. Wang , Q. Wu , Y. Mao‐Draayer , and C. H. Kim , “Bidirectional Regulatory Potentials of Short‐Chain Fatty Acids and Their G‐Protein‐Coupled Receptors in Autoimmune Neuroinflammation,” Scientific Reports 9, no. 1 (2019): 8837, 10.1038/s41598-019-45311-y. 31222050 PMC6586800

[62] L. Ho , K. Ono , M. Tsuji , P. Mazzola , R. Singh , and G. M. Pasinetti , “Protective Roles of Intestinal Microbiota Derived Short Chain Fatty Acids in Alzheimer's Disease‐Type Beta‐Amyloid Neuropathological Mechanisms,” Expert Review of Neurotherapeutics 18, no. 1 (2018): 83–90, 10.1080/14737175.2018.1400909. 29095058 PMC5958896

[63] Z. Wu , S. Huang , T. Li , et al., “Gut Microbiota from Green Tea Polyphenol‐Dosed Mice Improves Intestinal Epithelial Homeostasis and Ameliorates Experimental Colitis,” Microbiome 9, no. 1 (2021): 184, 10.1186/s40168-021-01115-9. 34493333 PMC8424887

[64] E. Barrett , R. P. Ross , P. W. O'Toole , G. F. Fitzgerald , and C. Stanton , “γ‐Aminobutyric Acid Production by Culturable Bacteria from the human Intestine,” Journal of Applied Microbiology 113, no. 2 (2012): 411–417, 10.1111/j.1365-2672.2012.05344.x. 22612585

[65] D. Ağagündüz , B. Kocaadam‐Bozkurt , O. Bozkurt , et al., “Microbiota Alteration and Modulation in Alzheimer's Disease by Gerobiotics: the Gut‐Health Axis for a Good Mind,” Biomedicine & Pharmacotherapy 153 (2022): 113430, 10.1016/j.biopha.2022.113430. 36076486

[66] R. Yaghoubfar , A. Behrouzi , F. Ashrafian , et al., “Modulation of Serotonin Signaling/Metabolism by Akkermansia Muciniphila and Its Extracellular Vesicles through the Gut‐Brain Axis in Mice,” Scientific Reports 10, no. 1 (2020): 22119, 10.1038/s41598-020-79171-8. 33335202 PMC7747642

[67] K. R. Jaberi , V. Alamdari‐palangi , A. Savardashtaki , et al., “Modulatory Effects of Phytochemicals on Gut–Brain Axis: Therapeutic Implication,” Current Developments in Nutrition 8, no. 6 (2024): 103785, 10.1016/j.cdnut.2024.103785. 38939650 PMC11208951

[68] Z.‐Z. Sun , X.‐Y. Li , S. Wang , L. Shen , and H.‐F. Ji , “Bidirectional Interactions between Curcumin and Gut Microbiota in Transgenic Mice with Alzheimer's Disease,” Applied Microbiology and Biotechnology 104, no. 8 (2020): 3507–3515, 10.1007/s00253-020-10461-x. 32095862

[69] J. Y. Chung , J.‐H. Jeong , and J. Song , “Resveratrol Modulates the Gut‐Brain Axis: Focus on Glucagon‐Like Peptide‐1, 5‐HT, and Gut Microbiota,” Frontiers in Aging Neuroscience 12 (2020): 588044, 10.3389/fnagi.2020.588044. 33328965 PMC7732484

[70] Y. Hu , Q. Lin , H. Zhao , et al., “Bioaccessibility and Bioavailability of Phytochemicals: Influencing Factors, Improvements, and Evaluations,” Food Hydrocolloids 135 (2023): 108165, 10.1016/j.foodhyd.2022.108165.

[71] C. Tan and D. J. McClements , “Application of Advanced Emulsion Technology in the Food Industry: a Review and Critical Evaluation,” Foods 10, no. 4 (2021): 812, 10.3390/foods10040812. 33918596 PMC8068840

[72] M. Manzoor , P. Sharma , M. Murtaza , A. K. Jaiswal , and S. Jaglan , “Fabrication, Characterization, and Interventions of Protein, Polysaccharide and Lipid‐Based Nanoemulsions in Food and Nutraceutical Delivery Applications: A Review,” International Journal of Biological Macromolecules 241 (2023): 124485, 10.1016/j.ijbiomac.2023.124485. 37076071

[73] Y. Hu , Q. Lin , H. Zhao , et al., “Bioaccessibility and Bioavailability of Phytochemicals: Influencing Factors, Improvements, and Evaluations,” Food Hydrocolloids 135 (2023): 108165, 10.1016/j.foodhyd.2022.108165.

[74] X.‐M. Li , X. Li , Z. Wu , et al., “Chitosan Hydrochloride/Carboxymethyl Starch Complex Nanogels Stabilized Pickering Emulsions for Oral Delivery of β‐Carotene: Protection Effect and in Vitro Digestion Study,” Food Chemistry 315 (2020): 126288, 10.1016/j.foodchem.2020.126288. 32032833

[75] M. Matos , G. Gutiérrez , L. Martínez‐Rey , O. Iglesias , and C. Pazos , “Encapsulation of Resveratrol Using Food‐Grade Concentrated Double Emulsions: Emulsion Characterization and Rheological Behaviour,” Journal of Food Engineering 226 (2018): 73–81, 10.1016/j.jfoodeng.2018.01.007.

[76] K. Yao , D. J. McClements , C. Yan , et al., “In Vitro and in Vivo Study of the Enhancement of Carotenoid Bioavailability in Vegetables Using Excipient Nanoemulsions: Impact of Lipid Content,” Food Research International 141 (2021): 110162, 10.1016/j.foodres.2021.110162. 33642022

[77] C. Tan and D. J. McClements , “Application of Advanced Emulsion Technology in the Food Industry: a Review and Critical Evaluation,” Foods 10, no. 4 (2021): 812, 10.3390/foods10040812. 33918596 PMC8068840

[78] H. Gao , L. Ma , C. Cheng , et al., “Review of Recent Advances in the Preparation, Properties, and Applications of High Internal Phase Emulsions,” Trends in Food Science & Technology 112 (2021): 36–49, 10.1016/j.tifs.2021.03.041.

[79] X. Leng , S. Cheng , H. Wu , Y. Nian , X. Zeng , and B. Hu , “High Internal Phase Emulsions Stabilized with Polyphenol‐Amyloid Fibril Supramolecules for Encapsulation and Protection of Lutein,” Journal of Agricultural and Food Chemistry 70, no. 7 (2022): 2328–2338, 10.1021/acs.jafc.1c04615. 35133823

[80] H. Wu , Y. Nian , Y. Liu , Y. Zhang , and B. Hu , “Formation of Pea Protein Amyloid Fibrils to Stabilize High Internal Phase Emulsions for Encapsulation of Lutein,” Journal of Functional Foods 94 (2022): 105110, 10.1016/j.jff.2022.105110.

[81] W. Li , Y. Nian , Y. Huang , X. Zeng , Q. Chen , and B. Hu , “High Loading Contents, Distribution and Stability of β‐Carotene Encapsulated in High Internal Phase Emulsions,” Food Hydrocolloids 96 (2019): 300–309, 10.1016/j.foodhyd.2019.05.038.

[82] Y.‐T. Xu , C.‐H. Tang , T.‐X. Liu , and R. Liu , “Ovalbumin as an Outstanding Pickering Nanostabilizer for High Internal Phase Emulsions,” Journal of Agricultural and Food Chemistry 66, no. 33 (2018): 8795–8804, 10.1021/acs.jafc.8b02183. 30044922

[83] H. Tan , L. Zhao , S. Tian , H. Wen , X. Gou , and T. Ngai , “Gelatin Particle‐Stabilized High‐Internal Phase Emulsions for Use in Oral Delivery Systems: Protection Effect and in Vitro Digestion Study,” Journal of Agricultural and Food Chemistry 65, no. 4 (2017): 900–907, 10.1021/acs.jafc.6b04705. 28064487

[84] J. Yi , L. Gao , G. Zhong , and Y. Fan , “Fabrication of High Internal Phase Pickering Emulsions with Calcium‐Crosslinked Whey Protein Nanoparticles for β‐Carotene Stabilization and Delivery,” Food & Function 11, no. 1 (2020): 768–778, 10.1039/c9fo02434d. 31917381

[85] X.‐N. Huang , F.‐Z. Zhou , T. Yang , S.‐W. Yin , C.‐H. Tang , and X.‐Q. Yang , “Fabrication and Characterization of Pickering High Internal Phase Emulsions (HIPEs) Stabilized by Chitosan‐Caseinophosphopeptides Nanocomplexes as Oral Delivery Vehicles,” Food Hydrocolloids 93 (2019): 34–45, 10.1016/j.foodhyd.2019.02.005.

[86] Z. Wei and Q. Huang , “Development of High Internal Phase Pickering Emulsions Stabilised by Ovotransferrin–Gum Arabic Particles as Curcumin Delivery Vehicles,” International Journal of Food Science & Technology 55, no. 5 (2020): 1891–1899, 10.1111/ijfs.14340.

[87] W. Wijaya , H. Zheng , T. Zheng , et al., “Improved Bioaccessibility of Polymethoxyflavones Loaded into High Internal Phase Emulsions Stabilized by Biopolymeric Complexes: a Dynamic Digestion Study via TNO's Gastrointestinal Model,” Current Research in Food Science 2 (2020): 11–19, 10.1016/j.crfs.2019.11.007. 32914106 PMC7473367

[88] W. Wijaya , H. Zheng , A. R. Patel , P. Van der Meeren , and Q. Huang , “Crystallization of Polymethoxyflavones in High Internal Phase Emulsions Stabilized Using Biopolymeric Complexes: Implications for Microstructure and *in Vitro* Digestion Properties,” Food Bioscience 40 (2021): 100876, 10.1016/j.fbio.2021.100876.

[89] S.‐K. Shen , Y.‐W. Chen , W.‐T. Yu , et al., “High Internal Phase Pickering Emulsions Stabilized by Modified Sturgeon Myofibrillar Protein for Quercetin Delivery,” Food Hydrocolloids 144 (2023): 108926, 10.1016/j.foodhyd.2023.108926.

[90] L. Salvia‐Trujillo , R. Soliva‐Fortuny , M. A. Rojas‐Graü , D. J. McClements , and O. Martín‐Belloso , “Edible Nanoemulsions as Carriers of Active Ingredients: a Review,” Annual Review of Food Science and Technology 8 (2017): 439–466, 10.1146/annurev-food-030216-025908. 28125342

[91] D. J. McClements , “Edible Nanoemulsions: Fabrication, Properties, and Functional Performance,” Soft Matter 7, no. 6 (2011): 2297–2316, 10.1039/C0SM00549E.

[92] R. Zhang , Z. Zhang , and D. J. McClements , “Nanoemulsions: an Emerging Platform for Increasing the Efficacy of Nutraceuticals in Foods,” Colloids and Surfaces B: Biointerfaces 194 (2020): 111202, 10.1016/j.colsurfb.2020.111202. 32585537

[93] X. Wang , Y. Nian , Z. Zhang , Q. Chen , X. Zeng , and B. Hu , “High Internal Phase Emulsions Stabilized with Amyloid Fibrils and Their Polysaccharide Complexes for Encapsulation and Protection of β‐Carotene,” Colloids and Surfaces B: Biointerfaces 183 (2019): 110459, 10.1016/j.colsurfb.2019.110459. 31499452

[94] C. Zhao , L. Wei , B. Yin , et al., “Encapsulation of Lycopene within Oil‐in‐Water Nanoemulsions Using Lactoferrin: Impact of Carrier Oils on Physicochemical Stability and Bioaccessibility,” International Journal of Biological Macromolecules 153 (2020): 912–920, 10.1016/j.ijbiomac.2020.03.063. 32169453

[95] Y.‐A. Zhu , P. Sun , C. Duan , et al., “Improving Stability and Bioavailability of Curcumin by Quaternized Chitosan Coated Nanoemulsion,” Food Research International 174, no. Pt 1 (2023): 113634, 10.1016/j.foodres.2023.113634. 37986538

[96] P. Zhu , J. He , S. Huang , L. Han , C. Chang , and W. Zhang , “Encapsulation of Resveratrol in Zein‐Polyglycerol Conjugate Stabilized O/W Nanoemulsions: Chemical Stability, In Vitro Gastrointestinal Digestion, and Antioxidant Activity,” LWT 149 (2021): 112049, 10.1016/j.lwt.2021.112049.

[97] A. S. Kadappan , C. Guo , C. E. Gumus , et al., “The Efficacy of Nanoemulsion‐Based Delivery to Improve Vitamin D Absorption: Comparison of In Vitro and In Vivo Studies,” Molecular Nutrition & Food Research 62, no. 4 (2018), 10.1002/mnfr.201700836. 29266712

[98] Y. Gao , X. Qi , Y. Zheng , et al., “Nanoemulsion Enhances α‐Tocopherol Succinate Bioavailability in Rats,” International Journal of Pharmaceutics 515, no. 1–2 (2016): 506–514, 10.1016/j.ijpharm.2016.10.026. 27746330

[99] Z. Niu , A. Acevedo‐Fani , A. McDowell , A. Barnett , S. M. Loveday , and H. Singh , “Nanoemulsion Structure and Food Matrix Determine the Gastrointestinal Fate and In Vivo Bioavailability of Coenzyme Q10,” Journal of Controlled Release 327 (2020): 444–455, 10.1016/j.jconrel.2020.08.025. 32853729

[100] G. Øye , S. Simon , T. Rustad , and K. Paso , “Trends in Food Emulsion Technology: Pickering, Nano‐, and Double Emulsions,” in Current Opinion in Food Science (2023), 101003, 10.1016/j.cofs.2023.101003.

[101] Y. Bai , Y. Sun , X. Li , et al., “Phycocyanin/Lysozyme Nanocomplexes to Stabilize Pickering Emulsions for Fucoxanthin Encapsulation,” Food Research International 173, no. Pt 2 (2023): 113386, 10.1016/j.foodres.2023.113386. 37803725

[102] J. Xiao , C. Li , and Q. Huang , “Kafirin Nanoparticle‐Stabilized Pickering Emulsions as Oral Delivery Vehicles: Physicochemical Stability and in Vitro Digestion Profile,” Journal of Agricultural and Food Chemistry 63, no. 47 (2015): 10263–10270, 10.1021/acs.jafc.5b04385. 26539628

[103] L. Bai , S. Huan , O. J. Rojas , and D. J. McClements , “Recent Innovations in Emulsion Science and Technology for Food Applications,” Journal of Agricultural and Food Chemistry 69, no. 32 (2021): 8944–8963, 10.1021/acs.jafc.1c01877. 33982568

[104] C. Chai and J. Park , “Food Liposomes: Structures, Components, Preparations, and Applications,” Food Chemistry 432 (2024): 137228, 10.1016/j.foodchem.2023.137228. 37633138

[105] Y. Dutt , R. P. Pandey , M. Dutt , et al., “Liposomes and Phytosomes: Nanocarrier Systems and Their Applications for the Delivery of Phytoconstituents,” Coordination Chemistry Reviews 491 (2023): 215251, 10.1016/j.ccr.2023.215251.

[106] M. R. Islam Shishir , N. Karim , V. Gowd , X. Zheng , and W. Chen , “Liposomal Delivery of Natural Product: A Promising Approach in Health Research,” Trends in Food Science & Technology 85 (2019): 177–200, 10.1016/j.tifs.2019.01.013.

[107] K. K. Ajeeshkumar , P. A. Aneesh , N. Raju , M. Suseela , C. N. Ravishankar , and S. Benjakul , “Advancements in Liposome Technology: Preparation Techniques and Applications in Food, Functional Foods, and Bioactive Delivery: a Review,” Comprehensive Reviews in Food Science and Food Safety 20, no. 2 (2021): 1280–1306, 10.1111/1541-4337.12725. 33665991

[108] Y. Ji , Z. Wang , X. Ju , F. Deng , F. Yang , and R. He , “Co‐encapsulation of Rutinoside and β‐carotene in Liposomes Modified by Rhamnolipid: Antioxidant Activity, Antibacterial Activity, Storage Stability, and In Vitro Gastrointestinal Digestion,” Journal of Food Science 88, no. 5 (2023): 2064–2077, 10.1111/1750-3841.16548. 37013971

[109] T. K. Giri , P. Mukherjee , T. K. Barman , and S. Maity , “Nano‐encapsulation of Capsaicin on Lipid Vesicle and Evaluation of Their Hepatocellular Protective Effect,” International Journal of Biological Macromolecules 88 (2016): 236–243, 10.1016/j.ijbiomac.2016.03.056. 27032489

[110] M. Marsanasco and S. D. V. Alonso , “Stability of Bioactive Compounds in Liposomes after Pasteurisation and Storage of Functional Chocolate Milk,” International Journal of Food Science & Technology 57, no. 1 (2022): 361–369, 10.1111/ijfs.15420.

[111] J. Hao , B. Guo , S. Yu , et al., “Encapsulation of the Flavonoid Quercetin with Chitosan‐Coated Nano‐liposomes,” LWT—Food Science and Technology 85 (2017): 37–44, 10.1016/j.lwt.2017.06.048.

[112] B. S. Pattni , V. V. Chupin , and V. P. Torchilin , “New Developments in Liposomal Drug Delivery,” Chemical Reviews 115, no. 19 (2015): 10938–10966, 10.1021/acs.chemrev.5b00046. 26010257

[113] S. Hua , “Lipid‐Based Nano‐Delivery Systems for Skin Delivery of Drugs and Bioactives,” Frontiers in Pharmacology 6 (2015): 219, 10.3389/fphar.2015.00219. 26483690 PMC4588690

[114] C. Chai and J. Park , “Food Liposomes: Structures, Components, Preparations, and Applications,” Food Chemistry 432 (2024): 137228, 10.1016/j.foodchem.2023.137228. 37633138

[115] M. Akanda , M. S. H. Mithu , and D. Douroumis , “Solid Lipid Nanoparticles: an Effective Lipid‐Based Technology for Cancer Treatment,” Journal of Drug Delivery Science and Technology 86 (2023): 104709, 10.1016/j.jddst.2023.104709.

[116] A. Zoabi , E. Touitou , and K. Margulis , “Recent Advances in Nanomaterials for Dermal and Transdermal Applications,” Colloids and Interfaces 5, no. 1 (2021): 18, 10.3390/colloids5010018.

[117] L. A. S. Bahari and H. Hamishehkar , “The Impact of Variables on Particle Size of Solid Lipid Nanoparticles and Nanostructured Lipid Carriers; A Comparative Literature Review,” Advanced Pharmaceutical Bulletin 6, no. 2 (2016), 10.15171/apb.2016.021. PMC4961971 27478775

[118] A. K. Mahor , P. P. Singh , R. Gupta , et al., “Nanostructured Lipid Carriers for Improved Delivery of Therapeutics via the Oral Route,” Journal of Nanotechnology 2023 (2023): 1–35, 10.1155/2023/4687959.

[119] M. Fathi , M. R. Mozafari , and M. Mohebbi , “Nanoencapsulation of Food Ingredients Using Lipid Based Delivery Systems,” Trends in Food Science & Technology 23, no. 1 (2012): 13–27, 10.1016/j.tifs.2011.08.003.

[120] M. Rohmah , A. Rahmadi , and S. Raharjo , “Bioaccessibility and Antioxidant Activity of β‐Carotene Loaded Nanostructured Lipid Carrier (NLC) from Binary Mixtures of Palm Stearin and Palm Olein,” Heliyon 8, no. 2 (2022): e08913, 10.1016/j.heliyon.2022.e08913. 35243052 PMC8857418

[121] C. Yang , H. Yan , X. Jiang , H. Xu , R. Tsao , and L. Zhang , “Preparation of 9Z‐β‐Carotene and 9Z‐β‐Carotene High‐Loaded Nanostructured Lipid Carriers: Characterization and Storage Stability,” Journal of Agricultural and Food Chemistry 68, no. 47 (2020): 13844–13853, 10.1021/acs.jafc.0c02342. 33164495

[122] S. Okonogi and P. Riangjanapatee , “Physicochemical Characterization of Lycopene‐Loaded Nanostructured Lipid Carrier Formulations for Topical Administration,” International Journal of Pharmaceutics 478, no. 2 (2015): 726–735, 10.1016/j.ijpharm.2014.12.002. 25479097

[123] X. Shu , J. Liu , L. Mao , F. Yuan , and Y. Gao , “Composite Hydrogels Filled with Rhamnolipid‐Based Nanoemulsion, Nanostructured Lipid Carrier, or Solid Lipid Nanoparticle: a Comparative Study on Gel Properties and the Delivery of Lutein,” Food Hydrocolloids 146 (2024): 109264, 10.1016/j.foodhyd.2023.109264.

[124] R. F. S. Gonçalves , J. T. Martins , L. Abrunhosa , et al., “Lipid‐Based Nanostructures as a Strategy to Enhance Curcumin Bioaccessibility: Behavior under Digestion and Cytotoxicity Assessment,” Food Research International 143 (2021): 110278, 10.1016/j.foodres.2021.110278. 33992378

[125] M. Gunawan and V. Boonkanokwong , “Current Applications of Solid Lipid Nanoparticles and Nanostructured Lipid Carriers as Vehicles in Oral Delivery Systems for Antioxidant Nutraceuticals: A Review,” Colloids and Surfaces B: Biointerfaces 233 (2024): 113608, 10.1016/j.colsurfb.2023.113608. 37925866

[126] R. Osanlou , M. Emtyazjoo , A. Banaei , M. A. Hesarinejad , and F. Ashrafi , “Preparation of Solid Lipid Nanoparticles and Nanostructured Lipid Carriers Containing Zeaxanthin and Evaluation of Physicochemical Properties,” Colloids and Surfaces A: Physicochemical and Engineering Aspects 641 (2022): 128588, 10.1016/j.colsurfa.2022.128588.

[127] D. J. McClements , “Advances in Nanoparticle and Microparticle Delivery Systems for Increasing the Dispersibility, Stability, and Bioactivity of Phytochemicals,” Biotechnology Advances 38 (2020): 107287, 10.1016/j.biotechadv.2018.08.004. 30086329

[128] S. Gao and D. J. McClements , “Formation and Stability of Solid Lipid Nanoparticles Fabricated Using Phase Inversion Temperature Method,” Colloids and Surfaces A: Physicochemical and Engineering Aspects 499 (2016): 79–87, 10.1016/j.colsurfa.2016.03.065.

[129] V. da Silva Santos , A. P. Badan Ribeiro , and M. H. Andrade Santana , “Solid Lipid Nanoparticles as Carriers for Lipophilic Compounds for Applications in Foods,” Food Research International 122 (2019): 610–626, 10.1016/j.foodres.2019.01.032. 31229120

[130] A. Babu , R. Shams , K. K. Dash , A. M. Shaikh , and B. Kovács , “Protein‐Polysaccharide Complexes and Conjugates: Structural Modifications and Interactions under Diverse Treatments,” Journal of Agriculture and Food Research 18 (2024): 101510, 10.1016/j.jafr.2024.101510.

[131] Y. Yang , X. Wang , G. Chen , et al., “SAXS Characterization of the Interactions among Digested Food Compounds and the Anti‐Oxidant and Anti‐Inflammatory Activities of the Formed Nanocomplexes,” Food & Function 9, no. 6 (2018): 3408–3418, 10.1039/c8fo00563j. 29873345

[132] Q. Ge , S. Rong , C. Yin , et al., “Calcium Ions Induced ι‐Carrageenan‐Based Gel‐Coating Deposited on Zein Nanoparticles for Encapsulating the Curcumin,” Food Chemistry 434 (2024): 137488, 10.1016/j.foodchem.2023.137488. 37741234

[133] J. Hao , J. Xu , W. Zhang , et al., “The Improvement of the Physicochemical Properties and Bioaccessibility of Lutein Microparticles by Electrostatic Complexation,” Food Hydrocolloids 125 (2022): 107381, 10.1016/j.foodhyd.2021.107381.

[134] D. J. McClements , “Designing Biopolymer Microgels to Encapsulate, Protect and Deliver Bioactive Components: Physicochemical Aspects,” Advances in Colloid and Interface Science 240 (2017): 31–59, 10.1016/j.cis.2016.12.005. 28034309

[135] T. Ramdhan , S. Hung Ching , S. Prakash , and B. Bhandari , “Evaluation of Alginate‐Biopolymers (Protein, Hydrocolloid, Starch) Composite Microgels Prepared by the Spray Aerosol Technique as a Carrier for Green Tea Polyphenols,” Food Chemistry 371 (2022): 131382, 10.1016/j.foodchem.2021.131382. 34808775

[136] W. Wang , W. Liu , J. Wu , et al., “Preparation and Characterization of Particle‐Filled Microgels by Chemical Cross‐Linking Based on Zein and Carboxymethyl Starch for Delivering the Quercetin,” Carbohydrate Polymers 323 (2024): 121375, 10.1016/j.carbpol.2023.121375. 37940242

[137] A. Cid‐Samamed , J. Rakmai , J. C. Mejuto , J. Simal‐Gandara , and G. Astray , “Cyclodextrins Inclusion Complex: Preparation Methods, Analytical Techniques and Food Industry Applications,” Food Chemistry 384 (2022): 132467, 10.1016/j.foodchem.2022.132467. 35219231

[138] C. Wang , X. Chen , and S. Liu , “Encapsulation of Tangeretin into Debranched‐Starch Inclusion Complexes: Structure, Properties and Stability,” Food Hydrocolloids 100 (2020): 105409, 10.1016/j.foodhyd.2019.105409.

[139] C. Muñoz‐Shugulí , C. P. Vidal , P. Cantero‐López , and J. Lopez‐Polo , “Encapsulation of Plant Extract Compounds Using Cyclodextrin Inclusion Complexes, Liposomes, Electrospinning and Their Combinations for Food Purposes,” Trends in Food Science & Technology 108 (2021): 177–186, 10.1016/j.tifs.2020.12.020.

[140] M. P. Kapoor , M. Moriwaki , K. Uguri , K. Kito , D. Timm , and A. Abe , “Improved Bioavailability of Hesperetin 7‐O‐Glucoside Inclusion Complex with β‐Cyclodextrin in Sprague‐Dawley Rats and Healthy Humans,” Journal of Functional Foods 107 (2023): 105708, 10.1016/j.jff.2023.105708.

[141] N. Li , B. Feng , Y. Bi , F. Kong , Z. Wang , and S. Tan , “Sulfobutyl Ether Cyclodextrin Inclusion Complexes Containing Tea Polyphenols: Preparation, Characterization, Antioxidant Activity, α‐Glucosidase Inhibition, and *in Vitro* Release Property,” Journal of Molecular Structure 1295 (2024): 136686, 10.1016/j.molstruc.2023.136686.

[142] E. M. Ahmed , “Hydrogel: Preparation, Characterization, and Applications: a Review,” Journal of Advanced Research 6, no. 2 (2015): 105–121, 10.1016/j.jare.2013.07.006. 25750745 PMC4348459

[143] N. Micale , A. Citarella , M. S. Molonia , et al., “Hydrogels for the Delivery of Plant‐Derived (Poly)Phenols,” Molecules (Basel, Switzerland) 25, no. 14 (2020): 3254, 10.3390/molecules25143254. 32708833 PMC7397257

[144] J. Zhao , R. Chen , D. Cheng , et al., “Extremely Ultrahigh Stretchable Starch‐Based Hydrogels with Continuous Hydrogen Bonding,” Advanced Functional Materials 35, no. 8 (2025): 2415530, 10.1002/adfm.202415530.

[145] C. Cui , Y. Jia , Q. Sun , et al., “Recent Advances in the Preparation, Characterization, and Food Application of Starch‐Based Hydrogels,” Carbohydrate Polymers 291 (2022): 119624, 10.1016/j.carbpol.2022.119624. 35698350

[146] F. Wang , R. Ma , J. Zhu , J. Zhan , J. Li , and Y. Tian , “Physicochemical Properties, *In Vitro* Digestibility, and pH‐Dependent Release Behavior of Starch–Steviol Glycoside Composite Hydrogels,” Food Chemistry 434 (2024): 137420, 10.1016/j.foodchem.2023.137420. 37696154

[147] J. Kang , Y.‐H. Kim , S.‐J. Choi , S.‐J. Rho , and Y.‐R. Kim , “Improving the Stability and Curcumin Retention Rate of Curcumin‐Loaded Filled Hydrogel Prepared Using 4αGTase‐Treated Rice Starch,” Foods 10, no. 1 (2021): 150, 10.3390/foods10010150. 33450818 PMC7828239

[148] Y. Zhang , L. Dong , L. Liu , Z. Wu , D. Pan , and L. Liu , “Recent Advances of Stimuli‐Responsive Polysaccharide Hydrogels in Delivery Systems: A Review,” Journal of Agricultural and Food Chemistry 70, no. 21 (2022): 6300–6316, 10.1021/acs.jafc.2c01080. 35578738

[149] L. Yan , R. Wang , H. Wang , et al., “Formulation and Characterization of Chitosan Hydrochloride and Carboxymethyl Chitosan Encapsulated Quercetin Nanoparticles for Controlled Applications in Foods System and Simulated Gastrointestinal Condition,” Food Hydrocolloids 84 (2018): 450–457, 10.1016/j.foodhyd.2018.06.025.

[150] R. Rodríguez‐Rodríguez , C. Carreón‐Álvarez , C. A. Cruz‐Medina , et al., “A Review of pH‐Responsive Chitosan‐Based Hydrogels for Drug Delivery Applications,” European Polymer Journal 237 (2025): 114173, 10.1016/j.eurpolymj.2025.114173.

[151] L. G. Gómez‐Mascaraque , C. Soler , and A. Lopez‐Rubio , “Stability and Bioaccessibility of EGCG within Edible Micro‐Hydrogels. Chitosan vs. Gelatin, A Comparative Study,” Food Hydrocolloids 61 (2016): 128–138, 10.1016/j.foodhyd.2016.05.009.

[152] Y. Fan , X. Zeng , J. Yi , and Y. Zhang , “Fabrication of Pea Protein Nanoparticles with Calcium‐Induced Cross‐Linking for the Stabilization and Delivery of Antioxidative Resveratrol,” International Journal of Biological Macromolecules 152 (2020): 189–198, 10.1016/j.ijbiomac.2020.02.248. 32105693

[153] X. Li , X. Cheng , G. Fan , and C. Wu , “Physicochemical and Sustained‐Release Properties of Double‐Crosslinked Apricot Polysaccharides Hydrogels Encapsulating Blueberry Anthocyanins,” Food Bioscience 69 (2025): 106647, 10.1016/j.fbio.2025.106647.

[154] H. Li , Y. Yuan , J. Zhu , T. Wang , D. Wang , and Y. Xu , “Zein/Soluble Soybean Polysaccharide Composite Nanoparticles for Encapsulation and Oral Delivery of Lutein,” Food Hydrocolloids 103 (2020): 105715, 10.1016/j.foodhyd.2020.105715.

[155] T. Wang , Y. Yang , W. Feng , R. Wang , and Z. Chen , “Co‐Folding of Hydrophobic Rice Proteins and Shellac in Hydrophilic Binary Microstructures for Cellular Uptake of Apigenin,” Food Chemistry 309 (2020): 125695, 10.1016/j.foodchem.2019.125695. 31704070

[156] K. Liu , Y.‐Y. Chen , X.‐Q. Zha , Q.‐M. Li , L.‐H. Pan , and J.‐P. Luo , “Research Progress on Polysaccharide/Protein Hydrogels: Preparation Method, Functional Property and Application as Delivery Systems for Bioactive Ingredients,” Food Research International 147 (2021): 110542, 10.1016/j.foodres.2021.110542. 34399519

[157] S. Noore , S. Pathania , P. Fuciños , C. P. O'Donnell , and B. K. Tiwari , Nanocarriers for Controlled Release and Target Delivery of Bioactive Compounds (Springer Nature Switzerland, 2024), 10.1007/978-3-031-57488-7.

[158] H. Chen , X. Tan , X. Han , et al., “Ferritin Nanocage Based Delivery Vehicles: from Single‐, Co‐ to Compartmentalized‐ Encapsulation of Bioactive or Nutraceutical Compounds,” Biotechnology Advances 61 (2022): 108037, 10.1016/j.biotechadv.2022.108037. 36152892

[159] R. Yang , L. Chen , T. Zhang , S. Yang , X. Leng , and G. Zhao , “Self‐Assembly of Ferritin Nanocages into Linear Chains Induced by Poly(α, L‐Lysine),” Chemical Communications 50, no. 4 (2013): 481–483, 10.1039/C3CC47847E. 24263180

[160] J. Zang , H. Chen , G. Zhao , F. Wang , and F. Ren , “Ferritin Cage for Encapsulation and Delivery of Bioactive Nutrients: from Structure, Property to Applications,” Critical Reviews in Food Science and Nutrition 57, no. 17 (2017): 3673–3683, 10.1080/10408398.2016.1149690. 26980693

[161] K. K. Kim , R. Kim , and S.‐H. Kim , “Crystal Structure of a Small Heat‐Shock Protein,” Nature 394, no. 6693 (1998): 595–599, 10.1038/29106. 9707123

[162] T. Suzuki , S. Kobayashi , K. Miyahira , M. Sugiyama , K. Katsuki , and M. Ishikawa , “DNA‐Binding Protein from Starvation Cells Traps Intracellular Free‐Divalent Iron and Plays an Important Role in Oxidative Stress Resistance in Acetobacter Pasteurianus NBRC 3283,” Journal of Bioscience and Bioengineering 131, no. 3 (2021): 256–263, 10.1016/j.jbiosc.2020.10.005. 33218820

[163] T. Zhang , C. Lv , L. Chen , G. Bai , G. Zhao , and C. Xu , “Encapsulation of Anthocyanin Molecules within a Ferritin Nanocage Increases Their Stability and Cell Uptake Efficiency,” Food Research International 62 (2014): 183–192, 10.1016/j.foodres.2014.02.041.

[164] X. Tan , Y. Liu , J. Zang , T. Zhang , and G. Zhao , “Hyperthermostability of Prawn Ferritin Nanocage Facilitates Its Application as a Robust Nanovehicle for Nutraceuticals,” International Journal of Biological Macromolecules 191 (2021): 152–160, 10.1016/j.ijbiomac.2021.09.067. 34547309

[165] C. Zhang , X. Zhang , and G. Zhao , “Ferritin Nanocage: a Versatile Nanocarrier Utilized in the Field of Food, Nutrition, and Medicine,” Nanomaterials 10, no. 9 (2020): 1894, 10.3390/nano10091894. 32971961 PMC7557750

[166] C. Gu , T. Zhang , C. Lv , Y. Liu , Y. Wang , and G. Zhao , “His‐Mediated Reversible Self‐Assembly of Ferritin Nanocages through Two Different Switches for Encapsulation of Cargo Molecules,” ACS nano 14, no. 12 (2020): 17080–17090, 10.1021/acsnano.0c06670. 33197176

[167] R. Mezzenga , P. Schurtenberger , A. Burbidge , and M. Michel , “Understanding Foods as Soft Materials,” Nature Materials 4, no. 10 (2005): 729–740, 10.1038/nmat1496. 16195765

[168] Y. Li , Y. Luo , X. Song , et al., “Enhancing Water Solubility of Phytosterols through Co‐Amorphization with Food‐Grade Coformers,” Current Research in Food Science 10 (2025): 100984, 10.1016/j.crfs.2025.100984. 39911602 PMC11795070

[169] V. K. N , S. M , R. K , L. G , and S. M , “Plant‐Extract‐Infused Edible Films as Natural Antimicrobial and Antioxidant Packaging for Chicken Meat,” (2026), 10.1039/D5FB00553A.

[170] A. Nicolescu , M. Babota , L. Barros , et al., “Bioaccessibility and Bioactive Potential of Different Phytochemical Classes from Nutraceuticals and Functional Foods,” Frontiers in Nutrition 10 (2023), 10.3389/fnut.2023.1184535. PMC10415696 37575331

[171] Y. Sun , C. Lei , R. Qiao , and C. Li , “Recent Advances in Carrier‐Free Natural Small Molecule Self‐Assembly for Drug Delivery,” Biomaterials Science 12, no. 24 (2024): 6237–6252, 10.1039/D4BM01153H. 39513256

[172] T. Goto and T. Kondo , “Structure and Molecular Stacking of Anthocyanins—Flower Color Variation,” Angewandte Chemie International Edition in English 30, no. 1 (1991): 17–33, 10.1002/anie.199100171.

[173] P. Trouillas , J. C. Sancho‐García , G. J. De FreitasV , M. Otyepka , and O. Dangles , “Stabilizing and Modulating Color by Copigmentation: Insights from Theory and Experiment,” Chemical Reviews 116, no. 9 (2016): 4937–4982, 10.1021/acs.chemrev.5b00507. 26959943

[174] H. Chen , M. Wang , L. Zhang , et al., “Anthocyanin Profiles and Color Parameters of Fourteen Grapes and Wines from the Eastern Foot of Helan Mountain in Ningxia,” Food Chemistry: X 24 (2024): 102034, 10.1016/j.fochx.2024.102034. 39659684 PMC11629261

[175] A. Bimpilas , D. Tsimogiannis , K. Balta‐Brouma , T. Lymperopoulou , and V. Oreopoulou , “Evolution of Phenolic Compounds and Metal Content of Wine during Alcoholic Fermentation and Storage,” Food Chemistry 178 (2015): 164–171, 10.1016/j.foodchem.2015.01.090. 25704697

[176] B. Gordillo , F. J. Rodríguez‐Pulido , M. L. González‐Miret , et al., “Application of Differential Colorimetry To Evaluate Anthocyanin–Flavonol–Flavanol Ternary Copigmentation Interactions in Model Solutions,” Journal of Agricultural and Food Chemistry 63, no. 35 (2015): 7645–7653, 10.1021/acs.jafc.5b00181. 25817598

[177] A. Fernandes , N. F. Brás , N. Mateus , and V. D. Freitas , “A Study of Anthocyanin Self‐Association by NMR Spectroscopy,” New Journal of Chemistry 39, no. 4 (2015): 2602–2611, 10.1039/C4NJ02339K.

[178] T. Ujihara and N. Hayashi , “Association of Catechin Molecules in Water: Quantitative Binding Study and Complex Structure Analysis,” Journal of Natural Products 79, no. 1 (2016): 66–73, 10.1021/acs.jnatprod.5b00658. 26720794

[179] N. Hayashi and T. Ujihara , “Conformations of Flavan‐3‐Ols in Water: Analysis Using Density Functional Theory,” Journal of Natural Products 80, no. 2 (2017): 319–327, 10.1021/acs.jnatprod.6b00704. 28124903

[180] T. Ujihara and N. Hayashi , “Complex Structures of Monoglucosylrutin with Ent‐Gallocatechin‐3‐ O‐Gallate and Epigallocatechin‐3‐ O‐Gallate in Aqueous Solutions and the Mechanism of Color Change Induced by Complexation,” Journal of Natural Products 82, no. 1 (2019): 2–8, 10.1021/acs.jnatprod.7b00817. 30589259

[181] W. Ma , A. G. Cheetham , and H. Cui , “Building Nanostructures with Drugs,” Nano Today 11, no. 1 (2016): 13–30, 10.1016/j.nantod.2015.11.003. 27066106 PMC4821422

[182] A. M. Gazzali , M. Lobry , L. Colombeau , et al., “Stability of Folic Acid under Several Parameters,” European Journal of Pharmaceutical Sciences 93 (2016): 419–430, 10.1016/j.ejps.2016.08.045. 27575880

[183] B. Frigerio , C. Bizzoni , G. Jansen , et al., “Folate Receptors and Transporters: Biological Role and Diagnostic/Therapeutic Targets in Cancer and Other Diseases,” Journal of experimental & clinical cancer research 38, no. 1 (2019): 125, 10.1186/s13046-019-1123-1. 30867007 PMC6417013

[184] O. Patil and S. Mohanty , “Why Folates Self‐Assemble: A Simulation‐Based Study,” Molecular Simulation 40, no. 14 (2014): 1147–1156, 10.1080/08927022.2013.854890.

[185] X. Lin , X. Huang , X. Tian , et al., “Natural Small‐Molecule‐Based Carrier‐Free Self‐Assembly Library Originated from Traditional Chinese Herbal Medicine,” ACS omega 7, no. 48 (2022): 43510–43521, 10.1021/acsomega.2c04098. 36506183 PMC9730315

[186] J. Wu , Y. Yu , Y. Wang , et al., “Four Yellow Monoterpenoid Quinoline Alkaloids from the Stem of *Tabernaemontana bovina* ,” Organic Letters 21, no. 12 (2019): 4554–4558, 10.1021/acs.orglett.9b01453. 31179705

[187] J. Hu , Z.‐X. Niu , and J.‐F. Wang , “Recent Advances in the Total Synthesis of Spirotryprostatin Alkaloids,” Molecules (Basel, Switzerland) 29, no. 7 (2024): 1655, 10.3390/molecules29071655. 38611934 PMC11013222

[188] M. Yin , J. Mou , L. Sun , Y. Deng , and X. Ren , “Insight on Structural Modification, Cytotoxic or Anti‐Proliferative Activity, Structure‐Activity Relationship of Berberine Derivatives,” Medicinal Chemistry 19, no. 9 (2023): 823–837, 10.2174/1573406419666230403120956. 37016520

[189] M. Chen , P. Wang , T. Li , et al., “Comprehensive Analysis of Huanglian Jiedu Decoction: Revealing the Presence of a Self‐Assembled Phytochemical Complex in Its Naturally‐Occurring Precipitate,” Journal of Pharmaceutical and Biomedical Analysis 195 (2021): 113820, 10.1016/j.jpba.2020.113820. 33303266

[190] T. Li , P. Wang , W. Guo , et al., “Natural Berberine‐Based Chinese Herb Medicine Assembled Nanostructures with Modified Antibacterial Application,” ACS nano 13, no. 6 (2019): 6770–6781, 10.1021/acsnano.9b01346. 31135129

[191] X. Huang , X. Liu , X. Lin , et al., “Thermodynamics Driving Phytochemical Self‐Assembly Morphological Change and Efficacy Enhancement Originated from Single and Co‐Decoction of Traditional Chinese Medicine,” Journal of Nanobiotechnology 20, no. 1 (2022): 527, 10.1186/s12951-022-01734-w. 36510210 PMC9743513

[192] X. Huang , P. Wang , T. Li , et al., “Self‐Assemblies Based on Traditional Medicine Berberine and Cinnamic Acid for Adhesion‐Induced Inhibition Multidrug‐Resistant *Staphylococcus aureus* ,” ACS applied materials & interfaces 12, no. 1 (2020): 227–237, 10.1021/acsami.9b17722. 31829617

[193] S. Gao , H. Zheng , S. Xu , et al., “Novel Natural Carrier‐Free Self‐Assembled Nanoparticles for Treatment of Ulcerative Colitis by Balancing Immune Microenvironment and Intestinal Barrier,” Advanced Healthcare Materials 12, no. 31 (2023): 2301826, 10.1002/adhm.202301826. 37681364

[194] X. Tian , P. Wang , T. Li , et al., “Self‐Assembled Natural Phytochemicals for Synergistically Antibacterial Application from the Enlightenment of Traditional Chinese Medicine Combination,” Acta Pharmaceutica Sinica B 10, no. 9 (2020): 1784–1795, 10.1016/j.apsb.2019.12.014. 33088696 PMC7564035

[195] R. Lin , Y. Wang , H. Cheng , X. Ye , S. Chen , and H. Pan , “Epigallocatechin‐3‐Gallate Stabilizes Aqueous Curcumin by Generating Nanoparticles and Its Application in Beverages,” Food Chemistry 444 (2024): 138655, 10.1016/j.foodchem.2024.138655. 38330612

[196] S. E. Hakim , S. Liu , R. Herzog , et al., “Expansion of the Stereochemical Space of Triterpenes by Mining Noncanonical Oxidosqualene Cyclases across the Diversity of Green Plants,” Journal of the American Chemical Society 147, no. 12 (2025): 10320–10330, 10.1021/jacs.4c16956. 40083114 PMC11951148

[197] D. W. Christianson , “Structural and Chemical Biology of Terpenoid Cyclases,” Chemical Reviews 117, no. 17 (2017): 11570–11648, 10.1021/acs.chemrev.7b00287. 28841019 PMC5599884

[198] L. Fan , B. Zhang , A. Xu , et al., “Carrier‐Free, Pure Nanodrug Formed by the Self‐Assembly of an Anticancer Drug for Cancer Immune Therapy,” Molecular Pharmaceutics 15, no. 6 (2018): 2466–2478, 10.1021/acs.molpharmaceut.8b00444. 29727577

[199] X. Guo , W. Luo , L. Wu , et al., “Natural Products from Herbal Medicine Self‐Assemble into Advanced Bioactive Materials,” Advanced Science 11 (2024): 2403388, 10.1002/advs.202403388. 39033533 PMC11425287

[200] Y. Sun , C. Lei , R. Qiao , and C. Li , “Recent Advances in Carrier‐Free Natural Small Molecule Self‐Assembly for Drug Delivery,” Biomaterials Science 12, no. 24 (2024): 6237–6252, 10.1039/d4bm01153h. 39513256

[201] M. H. P. H. Kloots , S. K. Schoustra , J. A. Dijksman , and M. M. J. Smulders , “Phase Separation in Supramolecular and Covalent Adaptable Networks,” Soft Matter 19, no. 16 (2023): 2857–2877, 10.1039/D3SM00047H. 37060135 PMC10131172

[202] V. Adibnia and R. J. Hill , “Universal Aspects of Hydrogel Gelation Kinetics, Percolation and Viscoelasticity from PA‐Hydrogel Rheology,” Journal of Rheology 60, no. 4 (2016): 541–548, 10.1122/1.4948428.

[203] X. Du , J. Zhou , J. Shi , and B. Xu , “Supramolecular Hydrogelators and Hydrogels: from Soft Matter to Molecular Biomaterials,” Chemical Reviews 115, no. 24 (2015): 13165–13307, 10.1021/acs.chemrev.5b00299. 26646318 PMC4936198

[204] H. Huang , W. Gong , X. Wang , W. He , Y. Hou , and J. Hu , “Self‐Assembly of Naturally Small Molecules into Supramolecular Fibrillar Networks for Wound Healing,” Advanced Healthcare Materials 11, no. 12 (2022): 2102476, 10.1002/adhm.202102476. 35306757

[205] J. Zhong , J. C. Mareque‐Rivas , X. Lan , and Y.‐X. Su , “Supramolecular Assembly of Triterpenoids: Current State and Biomedical Perspectives,” Aggregate 6, no. 8 (2025): 70081, 10.1002/agt2.70081.

[206] B. G. Bag and S. S. Dash , “First Self‐assembly Study of Betulinic Acid, a Renewable Nano‐sized, 6‐6‐6‐6‐5 Pentacyclic Monohydroxy Triterpenic Acid, 6‐6‐6‐6‐5 Pentacyclic Monohydroxy Triterpenic Acid,” Nanoscale 3, no. 11 (2011): 4564–4566, 10.1039/c1nr10886g. 21947431

[207] A. Saha , J. Adamcik , S. Bolisetty , S. Handschin , and R. Mezzenga , “Fibrillar Networks of Glycyrrhizic Acid for Hybrid Nanomaterials with Catalytic Features,” Angewandte Chemie International Edition 54, no. 18 (2015): 5408–5412, 10.1002/anie.201411875. 25759108

[208] X. Jia , Z. Yang , J. Lu , et al., “A Natural Self‐Assembled Hydrogel Alleviates Inflammatory Bowel Disease by Targeting Mitochondrial Dysfunction and NLRP3/Caspase‐1/GSDMD‐Mediated Pyroptosis,” Advanced Functional Materials (2025): 24160, 10.1002/adfm.202524160.

[209] Y. Hong , X. Liu , W. Mao , et al., “Injectable Glycyrrhizic Acid Hydrogel Microspheres with Synergistic Anti‐Senescence and Osteogenic Effects for Osteoporosis Therapy,” Journal of Biomedical Materials Research Part A 113, no. 11 (2025): 38016, 10.1002/jbm.a.38016. 41216672

[210] J. Zheng , R. Fan , H. Wu , et al., “Directed Self‐Assembly of Herbal Small Molecules into Sustained Release Hydrogels for Treating Neural Inflammation,” Nature Communications 10, no. 1 (2019): 1604, 10.1038/s41467-019-09601-3. PMC6453967 30962431

[211] Z. Wang , J. Lu , Z. Yuan , et al., “Natural Carrier‐Free Binary Small Molecule Self‐Assembled Hydrogel Synergize Antibacterial Effects and Promote Wound Healing by Inhibiting Virulence Factors and Alleviating the Inflammatory Response,” Small 19, no. 5 (2023): 2205528, 10.1002/smll.202205528. 36446719

[212] Z. Cui , X. Zhang , L. Zhou , et al., “A Carrier‐Free Injectable Hydrogel Self‐Assembled Using Natural Thymol and Glycyrrhizin for MRSA‐Infected Wound Healing in Rats,” Chemical Engineering Journal 489 (2024): 151418, 10.1016/j.cej.2024.151418.

[213] M. Hao , S. Wei , S. Su , Z. Tang , and Y. Wang , “A Multifunctional Hydrogel Fabricated by Direct Self‐Assembly of Natural Herbal Small Molecule Mangiferin for Treating Diabetic Wounds,” ACS Applied Materials & Interfaces 16, no. 19 (2024): 24221–24234, 10.1021/acsami.4c01265. 38709623

[214] T. Ishizu , H. Tsutsumi , and T. Sato , “Mechanism of Creaming down Based on Chemical Characterization of a Complex of Caffeine and Tea Catechins,” Chemical & Pharmaceutical Bulletin 64, no. 7 (2016): 676–686, 10.1248/cpb.c16-00131. 27373623

[215] T. Sato , Y. Kinoshita , H. Tsutsumi , H. Yamamoto , and T. Ishizu , “Characterization of Creaming Precipitate of Tea Catechins and Caffeine in Aqueous Solution,” Chemical and Pharmaceutical Bulletin 60, no. 9 (2012): 1182–1187, 10.1248/cpb.c12-00433. 22976328

[216] T. Ishizu , H. Tsutsumi , Y. Kinoshita , H. Mukaida , T. Sato , and S. Kajitani , “Properties of Precipitate of Creaming down by (−)‐Epigallocatechin‐3‐*O*‐gallate and Caffeine,” Chemical and Pharmaceutical Bulletin 62, no. 6 (2014): 552–558, 10.1248/cpb.c14-00045. 24881661

[217] T. Lührs , C. Ritter , M. Adrian , et al., “3D Structure of Alzheimer's Amyloid‐β(1–42) Fibrils,” Proceedings of the National Academy of Sciences 102 (2005): 17342–17347, 10.1073/pnas.0506723102. PMC1297669 16293696

[218] Y. Xu , C.‐M. Ma , Y. Yang , et al., “Food‐Derived Protein Amyloid‐Like Fibrils: Fibrillation Mechanism, Structure, and Recent Advances for the Stabilization of Emulsions,” Food Hydrocolloids 145 (2023): 109146, 10.1016/j.foodhyd.2023.109146.

[219] C. M. Dobson , “Protein Misfolding, Evolution and Disease,” Trends in Biochemical Sciences 24, no. 9 (1999): 329–332, 10.1016/s0968-0004(99)01445-0. 10470028

[220] Y. Cao and R. Mezzenga , “Food Protein Amyloid Fibrils: Origin, Structure, Formation, Characterization, Applications and Health Implications,” Advances in Colloid and Interface Science 269 (2019): 334–356, 10.1016/j.cis.2019.05.002. 31128463

[221] G. Wei , Z. Su , N. P. Reynolds , et al., “Self‐Assembling Peptide and Protein Amyloids: from Structure to Tailored Function in Nanotechnology,” Chemical Society Reviews 46, no. 15 (2017): 4661–4708, 10.1039/C6CS00542J. 28530745 PMC6364806

[222] P. C. Ke , R. Zhou , L. C. Serpell , et al., “Half a Century of Amyloids: Past, Present and Future,” Chemical Society Reviews 49, no. 15 (2020): 5473–5509, 10.1039/C9CS00199A. 32632432 PMC7445747

[223] C. M. Dobson , “Protein‐misfolding Diseases: Getting out of Shape,” Nature 418, no. 6899 (2002): 729–730, 10.1038/418729a. 12181546

[224] K. Herrup , “The Case for Rejecting the Amyloid Cascade Hypothesis,” Nature Neuroscience 18, no. 6 (2015): 794–799, 10.1038/nn.4017. 26007212

[225] D. Xu , J. Zhou , W. L. Soon , et al., “Food Amyloid Fibrils Are Safe Nutrition Ingredients Based on in‐Vitro and In‐Vivo Assessment,” Nature Communications 14, no. 1 (2023): 6806, 10.1038/s41467-023-42486-x. PMC10603083 37884488

[226] R. Hervas , M. J. Rau , Y. Park , et al., “Cryo‐EM Structure of a Neuronal Functional Amyloid Implicated in Memory Persistence in Drosophila,” Science 367, no. 6483 (2020): 1230–1234, 10.1126/science.aba3526. 32165583 PMC7182444

[227] D. Wu , J. Zhou , Y. Shen , et al., “Highly Adhesive Amyloid–Polyphenol Hydrogels for Cell Scaffolding,” Biomacromolecules 24, no. 1 (2023): 471–480, 10.1021/acs.biomac.2c01311. 36548941

[228] R. Riek and D. S. Eisenberg , “The Activities of Amyloids from a Structural Perspective,” Nature 539, no. 7628 (2016): 227–235, 10.1038/nature20416. 27830791

[229] J. B. Rothbard , J. J. Rothbard , L. Soares , C. G. Fathman , and L. Steinman , “Identification of a Common Immune Regulatory Pathway Induced by Small Heat Shock Proteins, Amyloid Fibrils, and Nicotine,” Proceedings of the National Academy of Sciences 115, no. 27 (2018): 7081–7086, 10.1073/pnas.1804599115. PMC6142248 29915045

[230] M. P. Kurnellas , C. M. Adams , R. A. Sobel , L. Steinman , and J. B. Rothbard , “Amyloid Fibrils Composed of Hexameric Peptides Attenuate Neuroinflammation,” Science Translational Medicine 5, no. 179 (2013): 179ra42, 10.1126/scitranslmed.3005681. PMC3684024 23552370

[231] D. K. V. Kumar , S. H. Choi , K. J. Washicosky , et al., “Amyloid‐β Peptide Protects against Microbial Infection in Mouse and Worm Models of Alzheimer's Disease,” Science Translational Medicine 8, no. 340 (2016): 340ra72, 10.1126/scitranslmed.aaf1059. PMC5505565 27225182

[232] M. Peydayesh , M. Bagnani , W. L. Soon , and R. Mezzenga , “Turning Food Protein Waste into Sustainable Technologies,” Chemical Reviews 123, no. 5 (2023): 2112–2154, 10.1021/acs.chemrev.2c00236. 35772093 PMC9999431

[233] M. R. Sawaya , S. Sambashivan , R. Nelson , et al., “Atomic Structures of Amyloid Cross‐β Spines Reveal Varied Steric Zippers,” Nature 447, no. 7143 (2007): 453–457, 10.1038/nature05695. 17468747

[234] Y. Shen , L. Posavec , S. Bolisetty , et al., “Amyloid Fibril Systems Reduce, Stabilize and Deliver Bioavailable Nanosized Iron,” Nature Nanotechnology 12, no. 7 (2017): 642–647, 10.1038/nnano.2017.58. 28436960

[235] G. Nyström , M. P. Fernández‐Ronco , S. Bolisetty , M. Mazzotti , and R. Mezzenga , “Amyloid Templated Gold Aerogels,” Advanced Materials 28, no. 3 (2016): 472–478, 10.1002/adma.201503465. 26592185

[236] B. Hu , S. Yu , C. Shi , et al., “Amyloid–Polyphenol Hybrid Nanofilaments Mitigate Colitis and Regulate Gut Microbial Dysbiosis,” ACS nano 14, no. 3 (2020): 2760–2776, 10.1021/acsnano.9b09125. 31961657

[237] B. Hu , M. Li , X. He , et al., “Flavonoid–amyloid Fibril Hybrid Hydrogels for Obesity Control via the Construction of Gut Microbiota,” Biomaterials Science 10, no. 13 (2022): 3597–3611, 10.1039/d2bm00366j. 35642606

[238] J. Bieschke , M. Herbst , T. Wiglenda , et al., “Small‐molecule Conversion of Toxic Oligomers to Nontoxic β‐sheet–rich Amyloid Fibrils,” Nature Chemical Biology 8, no. 1 (2011): 93–101, 10.1038/nchembio.719. 22101602

[239] J. Bieschke , J. Russ , R. P. Friedrich , et al., “EGCG Remodels Mature α‐synuclein and Amyloid‐β Fibrils and Reduces Cellular Toxicity,” Proceedings of the National Academy of Sciences 107, no. 17 (2010): 7710–7715, 10.1073/pnas.0910723107. PMC2867908 20385841

[240] Y. Nian , Y. Zhang , C. Ruan , and B. Hu , “Update of the Interaction between Polyphenols and Amyloid Fibrils,” Current Opinion in Food Science 43 (2022): 99–106, 10.1016/j.cofs.2021.11.005.

[241] A. Kakinen , J. Adamcik , B. Wang , et al., “Nanoscale Inhibition of Polymorphic and Ambidextrous IAPP Amyloid Aggregation with Small Molecules,” Nano Research 11, no. 7 (2018): 3636–3647, 10.1007/s12274-017-1930-7. 30275931 PMC6162064

[242] J. M. Jakubowski , A. A. Orr , D. A. Le , and P. Tamamis , “Interactions between Curcumin Derivatives and Amyloid‐β Fibrils: Insights from Molecular Dynamics Simulations,” Journal of Chemical Information and Modeling 60, no. 1 (2020): 289–305, 10.1021/acs.jcim.9b00561. 31809572 PMC7732148

[243] T. D. Martin , A. J. Malagodi , E. Y. Chi , and D. G. Evans , “Computational Study of the Driving Forces and Dynamics of Curcumin Binding to Amyloid‐β Protofibrils,” The Journal of Physical Chemistry B 123, no. 3 (2019): 551–560, 10.1021/acs.jpcb.8b09185. 30571122

[244] S. Wu , C. Liu , Y. Li , et al., “Inhibitory Mechanisms of Amentoflavone on Amyloid‐β Peptide Aggregation Revealed by Replica Exchange Molecular Dynamics,” Scientific Reports 15, no. 1 (2025): 24352, 10.1038/s41598-025-10623-9. 40628933 PMC12238462

[245] M. D. Arifur Rahim , S. L. Kristufek , S. Pan , J. J. Richardson , and F. Caruso , “Phenolic Building Blocks for the Assembly of Functional Materials,” Angewandte Chemie International Edition 58, no. 7 (2019): 1904–1927, 10.1002/anie.201807804. 30221440

[246] Y. Zeng , S. Mao , B. Huang , X. Ye , and J. Tian , “Formation of Tannic Acid‐Binding Ovalbumin Amyloid Fibril Hydrogels: Enhanced Antibacterial and Antioxidant Properties,” Food Hydrocolloids 156 (2024): 110333, 10.1016/j.foodhyd.2024.110333.

[247] Q. Chen , Y. Liu , Y. Li , et al., “Interaction and Binding Mechanism of Ovalbumin with Cereal Phenolic Acids: Improved Structure, Antioxidant Activity, Emulsifying and Digestion Properties for Potential Targeted Delivery Systems,” Food Research International 175 (2024): 113726, 10.1016/j.foodres.2023.113726. 38128987

[248] J. Zhou , S. Gowachirapant , C. Zeder , et al., “Oat Protein Nanofibril‐Iron Hybrids as a Stable, High‐Absorption Iron Delivery Platform for Human Nutrition,” (2025), 10.1101/2025.01.24.25321072. PMC12717008 41214296

[249] M. A. Khan , L. Chen , and L. Liang , “Improvement in Storage Stability and Resveratrol Retention by Fabrication of Hollow Zein‐Chitosan Composite Particles,” Food Hydrocolloids 113 (2021): 106477, 10.1016/j.foodhyd.2020.106477.

[250] M. A. Khan , C. Yue , Z. Fang , et al., “Alginate/Chitosan‐Coated Zein Nanoparticles for the Delivery of Resveratrol,” Journal of Food Engineering 258 (2019): 45–53, 10.1016/j.jfoodeng.2019.04.010.

[251] R. Mohseni , Z. ArabSadeghabadi , N. Ziamajidi , R. Abbasalipourkabir , and A. RezaeiFarimani , “Oral Administration of Resveratrol‐Loaded Solid Lipid Nanoparticle Improves Insulin Resistance through Targeting Expression of SNARE Proteins in Adipose and Muscle Tissue in Rats with Type 2 Diabetes,” Nanoscale Research Letters 14, no. 1 (2019): 227, 10.1186/s11671-019-3042-7. 31290033 PMC6616559

[252] J. Gómez‐Estaca , M. P. Balaguer , G. López‐Carballo , R. Gavara , and P. Hernández‐Muñoz , “Improving Antioxidant and Antimicrobial Properties of Curcumin by Means of Encapsulation in Gelatin through Electrohydrodynamic Atomization,” Food Hydrocolloids 70 (2017): 313–320, 10.1016/j.foodhyd.2017.04.019.

[253] F. Michaux , P. Durand , J. Jasniewski , and M. Linder , “Butter Solid Nanoparticles for Curcumin Encapsulation: Influence of Nanoparticles Size on Drug Loading,” European Journal of Lipid Science and Technology 118, no. 8 (2016): 1168–1178, 10.1002/ejlt.201500348.

[254] K. Nakagawa , T. Harigae , T. Miyazawa , et al., “Metabolic Fate of Poly‐(lactic‐*co*‐glycolic acid)‐Based Curcumin Nanoparticles Following Oral Administration,” International Journal of Nanomedicine 11 (2016): 3009–3022, 10.2147/IJN.S107442. 27418823 PMC4935090

[255] M. E. El‐Naggar , F. Al‐Joufi , M. Anwar , M. F. Attia , and M. A. El‐Bana , “Curcumin‐Loaded PLA‐PEG Copolymer Nanoparticles for Treatment of Liver Inflammation in Streptozotocin‐Induced Diabetic Rats,” Colloids and Surfaces B: Biointerfaces 177 (2019): 389–398, 10.1016/j.colsurfb.2019.02.024. 30785036

[256] K. Suktham , T. Koobkokkruad , T. Wutikhun , and S. Surassmo , “Efficiency of Resveratrol‐Loaded Sericin Nanoparticles: Promising Bionanocarriers for Drug Delivery,” International Journal of Pharmaceutics 537, no. 1–2 (2018): 48–56, 10.1016/j.ijpharm.2017.12.015. 29229512

[257] Z. Hong , Y. Xu , J.‐F. Yin , J. Jin , Y. Jiang , and Q. Du , “Improving the Effectiveness of (−)‐Epigallocatechin Gallate (EGCG) against Rabbit Atherosclerosis by EGCG‐Loaded Nanoparticles Prepared from Chitosan and Polyaspartic Acid,” Journal of Agricultural and Food Chemistry 62, no. 52 (2014): 12603–12609, 10.1021/jf504603n. 25483592

[258] J. Zhang , S. Nie , Y. Zu , et al., “Anti‐Atherogenic Effects of CD36‐Targeted Epigallocatechin Gallate‐Loaded Nanoparticles,” Journal of Controlled Release 303 (2019): 263–273, 10.1016/j.jconrel.2019.04.018. 30999008 PMC6579691

[259] A. MdK , A. L.‐M. MA , S. Jamil , R. Al‐Shdefat , M. N. Ansari , and F. Shakeel , “Development and Evaluation of PLGA Polymer Based Nanoparticles of Quercetin,” International Journal of Biological Macromolecules 92 (2016): 213–219, 10.1016/j.ijbiomac.2016.07.002. 27381585

[260] D. Sun‐Waterhouse , S. S. Wadhwa , and G. I. N. Waterhouse , “Spray‐Drying Microencapsulation of Polyphenol Bioactives: a Comparative Study Using Different Natural Fibre Polymers as Encapsulants,” Food and Bioprocess Technology 6, no. 9 (2013): 2376–2388, 10.1007/s11947-012-0946-y.

[261] A. R. Madureira , A. Pereira , P. M. Castro , and M. Pintado , “Production of Antimicrobial Chitosan Nanoparticles against Food Pathogens,” Journal of Food Engineering 167 (2015): 210–216, 10.1016/j.jfoodeng.2015.06.010.

[262] I. J. Arroyo‐Maya and D. J. McClements , “Biopolymer Nanoparticles as Potential Delivery Systems for Anthocyanins: Fabrication and Properties,” Food Research International 69 (2015): 1–8, 10.1016/j.foodres.2014.12.005.

[263] D. R. A. Muhammad , A. Sedaghat Doost , V. Gupta , et al., “Stability and Functionality of Xanthan Gum–Shellac Nanoparticles for the Encapsulation of Cinnamon Bark Extract,” Food Hydrocolloids 100 (2020): 105377, 10.1016/j.foodhyd.2019.105377.

[264] A. López‐Córdoba , L. Deladino , and M. Martino , “Release of Yerba Mate Antioxidants from Corn Starch–Alginate Capsules as Affected by Structure,” Carbohydrate Polymers 99 (2014): 150–157, 10.1016/j.carbpol.2013.08.026. 24274491

[265] Y. S. Loo , T. Madheswaran , R. Rajendran , and R. J. C. Bose , “Encapsulation of Berberine into Liquid Crystalline Nanoparticles to Enhance Its Solubility and Anticancer Activity in MCF7 Human Breast Cancer Cells,” Journal of Drug Delivery Science and Technology 57 (2020): 101756, 10.1016/j.jddst.2020.101756.

[266] S. Comincini , F. Manai , M. Sorrenti , et al., “Development of Berberine‐Loaded Nanoparticles for Astrocytoma Cells Administration and Photodynamic Therapy Stimulation,” Pharmaceutics 15, no. 4 (2023): 1078, 10.3390/pharmaceutics15041078. 37111564 PMC10146331

[267] M. A. Helal , A. M. Abdel‐Gawad , O. M. Kandil , et al., “Microfluidic‐Based Formulation of Essential Oils‐Loaded Chitosan Coated PLGA Particles Enhances Their Bioavailability and Nematocidal Activity,” Pharmaceutics 14, no. 10 (2022): 2030, 10.3390/pharmaceutics14102030. 36297465 PMC9608619

[268] D. Pandita , S. Kumar , N. Poonia , and V. Lather , “Solid Lipid Nanoparticles Enhance Oral Bioavailability of Resveratrol, a Natural Polyphenol,” Food Research International 62 (2014): 1165–1174, 10.1016/j.foodres.2014.05.059.

[269] R. B. Friedrich , B. Kann , K. Coradini , H. L. Offerhaus , R. C. R. Beck , and M. Windbergs , “Skin Penetration Behavior of Lipid‐Core Nanocapsules for Simultaneous Delivery of Resveratrol and Curcumin,” European Journal of Pharmaceutical Sciences 78 (2015): 204–213, 10.1016/j.ejps.2015.07.018. 26215463

[270] K. Coradini , F. O. Lima , C. M. Oliveira , et al., “Co‐Encapsulation of Resveratrol and Curcumin in Lipid‐Core Nanocapsules Improves Their in Vitro Antioxidant Effects,” European Journal of Pharmaceutics and Biopharmaceutics 88, no. 1 (2014): 178–185, 10.1016/j.ejpb.2014.04.009. 24780440

[271] G. Olga , C. Styliani , and R. G. Ioannis , “Coencapsulation of Ferulic and Gallic Acid in Hp‐b‐Cyclodextrin,” Food Chemistry 185 (2015): 33–40, 10.1016/j.foodchem.2015.03.058. 25952838

[272] L. G. Gómez‐Mascaraque , M. Martínez‐Sanz , M. J. Fabra , and A. López‐Rubio , “Development of Gelatin‐Coated ι‐Carrageenan Hydrogel Capsules by Electric Field‐Aided Extrusion. Impact of Phenolic Compounds on Their Performance,” Food Hydrocolloids 90 (2019): 523–533, 10.1016/j.foodhyd.2018.12.017.

[273] G. Granata , G. M. L. Consoli , R. Lo Nigro , and C. Geraci , “Hydroxycinnamic Acids Loaded in Lipid‐Core Nanocapsules,” Food Chemistry 245 (2018): 551–556, 10.1016/j.foodchem.2017.10.106. 29287408

[274] M. Paini , S. R. Daly , B. Aliakbarian , et al., “An Efficient Liposome Based Method for Antioxidants Encapsulation,” Colloids and Surfaces B: Biointerfaces 136 (2015): 1067–1072, 10.1016/j.colsurfb.2015.10.038. 26590900

[275] C.‐F. Hung , J.‐K. Chen , M.‐H. Liao , H.‐M. Lo , and J.‐Y. Fang , “Development and Evaluation of Emulsion‐Liposome Blends for Resveratrol Delivery,” Journal of Nanoscience and Nanotechnology 6, no. 9 (2006): 2950–2958, 10.1166/jnn.2006.420. 17048503

[276] M. Huang , C. Liang , C. Tan , et al., “Liposome Co‐Encapsulation as a Strategy for the Delivery of Curcumin and Resveratrol,” Food & Function 10, no. 10 (2019): 6447–6458, 10.1039/C9FO01338E. 31524893

[277] M. Pettinato , P. Trucillo , R. Campardelli , P. Perego , and E. Reverchon , “Bioactives Extraction from Spent Coffee Grounds and Liposome Encapsulation by a Combination of Green Technologies,” Chemical Engineering and Processing—Process Intensification 151 (2020): 107911, 10.1016/j.cep.2020.107911.

[278] S. Peng , L. Zou , W. Zhou , W. Liu , C. Liu , and D. J. McClements , “Encapsulation of Lipophilic Polyphenols into Nanoliposomes Using pH‐Driven Method: Advantages and Disadvantages,” Journal of Agricultural and Food Chemistry 67, no. 26 (2019): 7506–7511, 10.1021/acs.jafc.9b01602. 31184879

[279] Y. Sun , J. Chi , X. Ye , et al., “Nanoliposomes as Delivery System for Anthocyanins: Physicochemical Characterization, Cellular Uptake, and Antioxidant Properties,” LWT 139 (2021): 110554, 10.1016/j.lwt.2020.110554.

[280] I. Gülseren and M. Corredig , “Storage Stability and Physical Characteristics of Tea‐Polyphenol‐Bearing Nanoliposomes Prepared with Milk Fat Globule Membrane Phospholipids,” Journal of Agricultural and Food Chemistry 61, no. 13 (2013): 3242–3251, 10.1021/jf3045439. 23473473

[281] Q. Fan , L. Wang , Y. Song , Z. Fang , M. Subirade , and L. Liang , “Partition and Stability of Resveratrol in Whey Protein Isolate Oil‐in‐Water Emulsion: Impact of Protein and Calcium Concentrations,” International Dairy Journal 73 (2017): 128–135, 10.1016/j.idairyj.2017.06.002.

[282] J. Teixé‐Roig , G. Oms‐Oliu , M. Artiga‐Artigas , I. Odriozola‐Serrano , and O. Martín‐Belloso , “Enhanced In Vivo Absorption and Biodistribution of Curcumin Loaded into Emulsions with High Medium‐Chain Triglyceride Content,” Food Research International 174 (2023): 113595, 10.1016/j.foodres.2023.113595. 37986458

[283] W. Gao , J. Zhu , P. Liu , B. Cui , and A. M. Abd El‐Aty , “Preparation and Characterization of Octenyl Succinylated Starch Microgels via a Water‐in‐Oil (W/O) Inverse Microemulsion Process for Loading and Releasing Epigallocatechin Gallate,” Food Chemistry 355 (2021): 129661, 10.1016/j.foodchem.2021.129661. 33848937

[284] M. Wang , N. Ji , M. Li , et al., “Fabrication and Characterization of Starch Beads Formed by a Dispersion‐Inverse Gelation Process for Loading Polyphenols with Improved Antioxidation,” Food Hydrocolloids 101 (2020): 105565, 10.1016/j.foodhyd.2019.105565.

[285] X. Luo , R. Guan , X. Chen , M. Tao , J. Ma , and J. Zhao , “Optimization on Condition of Epigallocatechin‐3‐Gallate (EGCG) Nanoliposomes by Response Surface Methodology and Cellular Uptake Studies in Caco‐2 Cells,” Nanoscale Research Letters 9, no. 1 (2014): 291, 10.1186/1556-276X-9-291. 24959109 PMC4059483

[286] D. A. Pai , V. R. Vangala , J. W. Ng , W. K. Ng , and R. B. H. Tan , “Resistant Maltodextrin as a Shell Material for Encapsulation of Naringin: Production and Physicochemical Characterization,” Journal of Food Engineering 161 (2015): 68–74, 10.1016/j.jfoodeng.2015.03.037.

[287] S. Scalia , M. R. Zampino , V. Trotta , and A. Bianchi , “Enhancement of Trans‐Resveratrol Photostability by Encapsulation in Lipid Microparticles: in Vitro and in Vivo Studies,” , no. 4 (2017): 200–204, 10.1691/ph.2017.6180. 29441987

[288] N. Acosta , E. Sánchez , L. Calderón , et al., “Physical Stability Studies of Semi‐Solid Formulations from Natural Compounds Loaded with Chitosan Microspheres,” Marine Drugs 13, no. 9 (2015): 5901–5919, 10.3390/md13095901. 26389926 PMC4584360

[289] C. Urzúa , E. González , V. Dueik , P. Bouchon , B. Giménez , and P. Robert , “Olive Leaves Extract Encapsulated by Spray‐Drying in Vacuum Fried Starch–Gluten Doughs,” Food and Bioproducts Processing 106 (2017): 171–180, 10.1016/j.fbp.2017.10.001.

[290] N. D. Aceval Arriola , P. M. de Medeiros , E. S. Prudencio , C. M. O. Müller , and R. D. de Mello Castanho Amboni , “Encapsulation of Aqueous Leaf Extract of *Stevia rebaudiana* Bertoni with Sodium Alginate and Its Impact on Phenolic Content,” Food Bioscience 13 (2016): 32–40, 10.1016/j.fbio.2015.12.001.

[291] E. Nakilcioğlu‐Taş and S. Ötleş , “Polyphenols from Olive Stones: Extraction with a Pilot Scale Pressurized Water Extractor, Microencapsulation by Spray‐Dryer and Storage Stability Evaluation,” Journal of Food Measurement and Characterization 14, no. 2 (2020): 849–861, 10.1007/s11694-019-00333-y.

[292] M. Ammendola , M. Haponska , K. Balik , et al., “Stability and Anti‐Proliferative Properties of Biologically Active Compounds Extracted from Cistus L. After Sterilization Treatments,” Scientific Reports 10, no. 1 (2020): 6521, 10.1038/s41598-020-63444-3. 32300137 PMC7162948

[293] L. Sheng , P. K. Jena , H.‐X. Liu , et al., “Obesity Treatment by Epigallocatechin‐3‐Gallate−Regulated Bile Acid Signaling and Its Enriched Akkermansia Muciniphila,” The FASEB Journal 32, no. 12 (2018): fj201800370R, 10.1096/fj.201800370R. PMC6219838 29882708

[294] K. Ning , K. Lu , Q. Chen , et al., “Epigallocatechin Gallate Protects Mice against Methionine–Choline‐Deficient‐Diet‐Induced Nonalcoholic Steatohepatitis by Improving Gut Microbiota to Attenuate Hepatic Injury and Regulate Metabolism,” ACS Omega 5, no. 33 (2020): 20800–20809, 10.1021/acsomega.0c01689. 32875214 PMC7450495

[295] Z. T. Bitzer , R. J. Elias , M. Vijay‐Kumar , and J. D. Lambert , “(‐)‐Epigallocatechin‐3‐Gallate Decreases Colonic Inflammation and Permeability in a Mouse Model of Colitis, but Reduces Macronutrient Digestion and Exacerbates Weight Loss,” Molecular Nutrition & Food Research 60, no. 10 (2016): 2267–2274, 10.1002/mnfr.201501042. 27218415 PMC13158975

[296] J. Pang , H. Xu , X. Wang , et al., “Resveratrol Enhances Trans‐Intestinal Cholesterol Excretion through Selective Activation of Intestinal Liver X Receptor Alpha,” Biochemical Pharmacology 186 (2021): 114481, 10.1016/j.bcp.2021.114481. 33631191

[297] P. Wang , J. Gao , W. Ke , et al., “Resveratrol Reduces Obesity in High‐Fat Diet‐Fed Mice via Modulating the Composition and Metabolic Function of the Gut Microbiota,” Free Radical Biology and Medicine 156 (2020): 83–98, 10.1016/j.freeradbiomed.2020.04.013. 32305646

[298] N. Sreng , S. Champion , J.‐C. Martin , et al., “Resveratrol‐Mediated Glycemic Regulation Is Blunted by Curcumin and Is Associated to Modulation of Gut Microbiota,” The Journal of Nutritional Biochemistry 72 (2019): 108218, 10.1016/j.jnutbio.2019.108218. 31473511

[299] P. Wang , J. Wang , D. Li , W. Ke , F. Chen , and X. Hu , “Targeting the Gut Microbiota with Resveratrol: A Demonstration of Novel Evidence for the Management of Hepatic Steatosis,” The Journal of Nutritional Biochemistry 81 (2020): 108363, 10.1016/j.jnutbio.2020.108363. 32388250

[300] P. Wang , D. Li , W. Ke , D. Liang , X. Hu , and F. Chen , “Resveratrol‐Induced Gut Microbiota Reduces Obesity in High‐Fat Diet‐Fed Mice,” International Journal of Obesity 44, no. 1 (2020): 213–225, 10.1038/s41366-019-0332-1. 30718820

[301] C. L. Campbell , R. Yu , F. Li , et al., “Modulation of Fat Metabolism and Gut Microbiota by Resveratrol on High‐Fat Diet‐Induced Obese Mice,” Diabetes, Metabolic Syndrome and Obesity: Targets and Therapy 12 (2019): 97–107, 10.2147/DMSO.S192228. 30655683 PMC6324607

[302] N. S. Bhandarkar , L. Brown , and S. K. Panchal , “Chlorogenic Acid Attenuates High‐Carbohydrate, High‐Fat Diet–Induced Cardiovascular, Liver, and Metabolic Changes in Rats,” Nutrition Research 62 (2019): 78–88, 10.1016/j.nutres.2018.11.002. 30803509

[303] S. Zhang , C. Ohland , C. Jobin , and S. Sang , “Black Tea Theaflavin Detoxifies Metabolic Toxins in the Intestinal Tract of Mice,” Molecular Nutrition & Food Research 65, no. 4 (2021): 2000887, 10.1002/mnfr.202000887. PMC7967262 33381889

[304] F. Huang , X. Zheng , X. Ma , et al., “Theabrownin from Pu‐Erh Tea Attenuates Hypercholesterolemia via Modulation of Gut Microbiota and Bile Acid Metabolism,” Nature Communications 10, no. 1 (2019): 4971, 10.1038/s41467-019-12896-x. PMC6823360 31672964

[305] X. Gao , Q. Xie , P. Kong , et al., “Polyphenol‐ and Caffeine‐Rich Postfermented Pu‐Erh Tea Improves Diet‐Induced Metabolic Syndrome by Remodeling Intestinal Homeostasis in Mice,” Infection and Immunity 86, no. 1 (2018): e00601, 10.1128/IAI.00601-17. 29061705 PMC5736808

[306] S. M. Henning , J. Yang , M. Hsu , et al., “Decaffeinated Green and Black Tea Polyphenols Decrease Weight Gain and Alter Microbiome Populations and Function in Diet‐Induced Obese Mice,” European Journal of Nutrition 57, no. 8 (2018): 2759–2769, 10.1007/s00394-017-1542-8. 28965248 PMC7367598

[307] Y. Xia , D. Tan , R. Akbary , J. Kong , R. Seviour , and Y. Kong , “Aqueous Raw and Ripe Pu‐Erh Tea Extracts Alleviate Obesity and Alter Cecal Microbiota Composition and Function in Diet‐Induced Obese Rats,” Applied Microbiology and Biotechnology 103, no. 4 (2019): 1823–1835, 10.1007/s00253-018-09581-2. 30610284

[308] D. E. Roopchand , R. N. Carmody , P. Kuhn , et al., “Dietary Polyphenols Promote Growth of the Gut Bacterium Akkermansia Muciniphila and Attenuate High‐Fat Diet–Induced Metabolic Syndrome,” Diabetes 64, no. 8 (2015): 2847–2858, 10.2337/db14-1916. 25845659 PMC4512228

[309] J. Baldwin , B. Collins , P. G. Wolf , et al., “Table Grape Consumption Reduces Adiposity and Markers of Hepatic Lipogenesis and Alters Gut Microbiota in Butter Fat‐Fed Mice,” The Journal of Nutritional Biochemistry 27 (2016): 123–135, 10.1016/j.jnutbio.2015.08.027. 26423887 PMC4933288

[310] L. Ho , D. Zhao , K. Ono , et al., “Heterogeneity in Gut Microbiota Drive Polyphenol Metabolism That Influences α‐Synuclein Misfolding and Toxicity,” The Journal of Nutritional Biochemistry 64 (2019): 170–181, 10.1016/j.jnutbio.2018.10.019. 30530257 PMC6363841

[311] C. Fang , H. Kim , L. Yanagisawa , et al., “Gallotannins and Lactobacillus Plantarum WCFS1 Mitigate High‐Fat Diet‐Induced Inflammation and Induce Biomarkers for Thermogenesis in Adipose Tissue in Gnotobiotic Mice,” Molecular Nutrition & Food Research 63, no. 9 (2019): 1800937, 10.1002/mnfr.201800937. 30908878

[312] B. Li , Z. Cheng , X. Sun , et al., “Lonicera caerulea L. Polyphenols Alleviate Oxidative Stress‐Induced Intestinal Environment Imbalance and Lipopolysaccharide‐Induced Liver Injury in HFD‐Fed Rats by Regulating the Nrf2/HO‐1/NQO1 and MAPK Pathways,” Molecular Nutrition & Food Research 64, no. 10 (2020): 1901315, 10.1002/mnfr.201901315. 32250024

[313] M. Zhang , Y. Xin , K. Feng , et al., “Comparative Analyses of Bioavailability, Biotransformation, and Excretion of Nobiletin in Lean and Obese Rats,” Journal of Agricultural and Food Chemistry 68, no. 39 (2020): 10709–10718, 10.1021/acs.jafc.0c04425. 32880448

[314] C.‐E. Guo , Q. Cui , J. Cheng , et al., “Probiotic‐Fermented Chinese Dwarf Cherry [*Cerasus humilis* (Bge.) Sok.] Juice Modulates the Intestinal Mucosal Barrier and Increases the Abundance of Akkermansia in the Gut in Association with Polyphenols,” Journal of Functional Foods 80 (2021): 104424, 10.1016/j.jff.2021.104424.

[315] A. M. Neyrinck , V. F. Van Hée , L. B. Bindels , F. De Backer , P. D. Cani , and N. M. Delzenne , “Polyphenol‐Rich Extract of Pomegranate Peel Alleviates Tissue Inflammation and Hypercholesterolaemia in High‐Fat Diet‐Induced Obese Mice: Potential Implication of the Gut Microbiota,” British Journal of Nutrition 109, no. 5 (2013): 802–809, 10.1017/S0007114512002206. 22676910

[316] J. Kan , C. Chen , T. Huo , et al., “Polyphenolic‐Enriched Peach Peels Extract Regulates Lipid Metabolism and Improves the Gut Microbiota Composition in High Fat Diet‐Fed Mice,” Journal of Functional Foods 72 (2020): 104082, 10.1016/j.jff.2020.104082.

[317] J. F. Lu , M. Q. Zhu , H. Zhang , et al., “Neohesperidin Attenuates Obesity by Altering the Composition of the Gut Microbiota in High‐Fat Diet‐Fed Mice,” The FASEB Journal 34, no. 9 (2020): 12053–12071, 10.1096/fj.201903102RR. 32729978

[318] C. Zhang , W. Wu , X. Li , X. Xin , and D. Liu , “Daily Supplementation with Fresh *Angelica keiskei* Juice Alleviates High‐Fat Diet‐Induced Obesity in Mice by Modulating Gut Microbiota Composition,” Molecular Nutrition & Food Research 63, no. 14 (2019): 1900248, 10.1002/mnfr.201900248. 31175701

[319] S. Ramos‐Romero , A. Léniz , D. Martínez‐Maqueda , et al., “Inter‐Individual Variability in Insulin Response after Grape Pomace Supplementation in Subjects at High Cardiometabolic Risk: Role of Microbiota and miRNA,” Molecular Nutrition & Food Research 65, no. 2 (2021): 2000113, 10.1002/mnfr.202000113. 33202108

[320] J. Rodríguez‐Morató , N. R. Matthan , J. Liu , R. de la Torre , and C.‐Y. O. Chen , “Cranberries Attenuate Animal‐Based Diet‐Induced Changes in Microbiota Composition and Functionality: a Randomized Crossover Controlled Feeding Trial,” The Journal of Nutritional Biochemistry 62 (2018): 76–86, 10.1016/j.jnutbio.2018.08.019. 30269035

[321] M. Paquette , A. S. Medina Larqué , S. J. Weisnagel , et al., “Strawberry and Cranberry Polyphenols Improve Insulin Sensitivity in Insulin‐Resistant, Non‐Diabetic Adults: a Parallel, Double‐Blind, Controlled and Randomised Clinical Trial,” British Journal of Nutrition 117, no. 4 (2017): 519–531, 10.1017/S0007114517000393. 28290272 PMC5426341

